# Chronic health effects associated with electronic cigarette use: A systematic review

**DOI:** 10.3389/fpubh.2022.959622

**Published:** 2022-10-06

**Authors:** Rania A. Wasfi, Felix Bang, Margaret de Groh, Andre Champagne, Arum Han, Justin J. Lang, Steven R. McFaull, Alexandria Melvin, Andrew Lawrence Pipe, Shika Saxena, Wendy Thompson, Emily Warner, Stephanie A. Prince

**Affiliations:** ^1^Applied Research Division, Centre for Surveillance and Applied Research, Health Promotion and Chronic Disease Prevention Branch, Public Health Agency of Canada, Ottawa, ON, Canada; ^2^Surveillance and Epidemiology Division, Centre for Immunization and Respiratory Infectious Diseases, Infectious Diseases Programs Branch, Public Health Agency of Canada, Ottawa, ON, Canada; ^3^Behaviours, Environments and Lifespan Division, Centre for Surveillance and Applied Research, Health Promotions and Chronic Disease Prevention Branch, Public Health Agency of Canada, Ottawa, ON, Canada; ^4^School of Epidemiology and Public Health, Faculty of Medicine, University of Ottawa, Ottawa, ON, Canada; ^5^Centre for Indigenous Statistics and Partnerships, Statistics Canada, Ottawa, ON, Canada; ^6^Faculty of Medicine, University of Ottawa, Ottawa, ON, Canada; ^7^Division of Cardiac Prevention & Rehabilitation, University of Ottawa Heart Institute, Ottawa, ON, Canada; ^8^Vaccine Safety, Vaccine Surveillance, Public Health Agency of Canada, Ottawa, ON, Canada

**Keywords:** vaping, E-cigarette (e-cig), electronic cigarette, health, chronic health effects

## Abstract

**Introduction:**

Over the last decade, e-cigarette use has been on the rise but with growing health concerns. The objective of this systematic review was to update findings for chronic health outcomes associated with e-cigarette use from the 2018 National Academies of Sciences, Engineering, and Medicine (NASEM) report.

**Methods:**

Three bibliographic databases were searched to identify studies comparing the chronic health effects of e-cigarette users (ECU) to non-smokers (NS), smokers, and/or dual users indexed between 31 August 2017 and 29 January 2021. Two independent reviewers screened abstracts and full texts. Data were extracted by one reviewer and verified by a second one. Outcomes were synthesized in a narrative manner using counts and based on statistical significance and direction of the association stratified by study design and exposure type. Risk of bias and certainty of evidence was assessed. The protocol was prospectively registered on Open Science Framework https://osf.io/u9btp.

**Results:**

A total of 180 articles were eligible. This review focused on 93 studies for the 11 most frequently reported outcomes and from which 59 reported on daily e-cigarette use. The certainty of evidence for all outcomes was very low because of study design (84% cross-sectional) and exposure type (27% reported on exclusive ECU, i.e., never smoked traditional cigarettes). Overall, the summary of results for nearly all outcomes, including inflammation, immune response, periodontal and peri-implant clinical parameters, lung function, respiratory symptoms, and cardiovascular disease, suggested either non-significant or mixed results when daily ECU was compared to NS. This was also observed when comparing exclusive ECU to NS. The only notable exception was related to oral health where most (11/14) studies reported significantly higher inflammation among daily ECU vs. NS. Compared to the smokers, the exclusive-ECUs had no statistically significant differences in inflammation orperiodontal clinical parameters but had mixed findings for peri-implant clinical parameters.

**Conclusions:**

This review provides an update to the 2018 NASEM report on chronic health effects of e-cigarette use. While the number of studies has grown, the certainty of evidence remains very low largely because of cross-sectional designs and lack of reporting on exclusive e-cigarette exposure. There remains a need for higher quality intervention and prospective studies to assess causality, with a focus on exclusive e-cigarette use.

## Introduction

Electronic cigarettes (e-cigarettes) were introduced in North America in 2006 (1). Since their introduction, e-cigarette use, also known as vaping, has been on the rise in Canada, the United States (US) (2–6), and European countries (7). Globally, estimation of the number of adult e-cigarette users has risen from 58.1 million in 2018 to 68 million in 2020 (8). Vaping is defined as the inhalation of vapor produced by heating liquids typically containing nicotine and flavoring elements, including heat-not-burn (HNB) tobacco products. E-cigarette use devices are battery-operated, contain a heating element, a mouthpiece, and a chamber to hold the liquid (1, 9). Vaping liquids generally contain propylene glycol, glycerin, and flavorings, and may contain nicotine (1, 10). E-cigarette use devices are not exclusively used to vape a liquid, they may also be used to vape tetrahydrocannabinol (THC) (11), dried cannabis, tobacco products, or psychoactive substances (12). A survey conducted among high school students in seven European countries from 2016 to 2017 showed that on average, 34% of the students had tried e-cigarettes (13). Among high school students in the US, e-cigarette use increased from 1.5% (220,000 individuals) in 2011 to 20.8% (3.05 million) in 2018 (6). In Canada, e-cigarette use among youth increased from 11% in 2013 to 16% in 2019 (14, 15). Similarly in Italy, youth e-cigarette users substantially increased from 0% in 2010 to 7.4% in 2014 and to 17.5% in 2018, and exclusive e-cigarette users recorded an almost three-fold significant increase from 2.9% in 2014 to 8.2% in 2018 (16). More recent data continue to show substantial increases in the proportion of youth who vape in Canada and the US (17).

Vaping has been associated with poisonings as a result of ingesting vaping liquids and burns associated with device malfunctions. Other acute health effects such as increased heart rate shortly after vaping, endothelial dysfunction, modulation of the sympathetic nervous system, and changes in pulmonary physiology have been documented (1, 11, 18–24). More recently, vaping has emerged as a significant public health issue because of rapid rise in the number of hospitalizations for what has been termed e-cigarette- or vaping use-associated lung injury (EVALI/ VALI) (25). As of February 2020, the Centers for Disease Control and Prevention in the US had identified 2,807 known cases of EVALI hospitalizations or deaths (26). Between 1 September 2019 and 31 December 2020, 20 cases of vaping-associated lung illness were detected by active surveillance in Canada (27, 28). These emerging vaping-related health issues have contributed to the need to better understand the broader health impacts of vaping.

To date, systematic reviews of peer-reviewed literature are largely categorized into two groups: those examining the health effects of vaping (10, 29–37) and those examining the efficacy of e-cigarettes as a smoking cessation aid (38, 39). Among those that have examined the health effects associated with vaping, findings have mostly been inconclusive. A number of study limitations have likely contributed to the equivocal results including limited sample size, cross-sectional design or lack of long-term follow-up, imprecision concerning exposure definitions, overall small number of available studies, and conflicts of interest where studies were funded or associated with e-cigarette manufacturers. It must be recognized that the emergence of significant health impacts of e-cigarette use may require significant longer-term exposure and review than is currently possible given their relatively recent introduction to the marketplace.

In 2018, the National Academies of Sciences, Engineering, and Medicine (NASEM) published the most comprehensive report to date addressing the health consequences of e-cigarette use. This report concluded that vaping was associated with some physiological effects on humans (e.g., acute changes in heart rate, possible increased risk of periodontal disease, increase in incident injuries, and poisoning). However, evidence of the long-term chronic health effects was not yet available, particularly of outcomes related to cardiovascular and respiratory health, immune biomarkers (e.g., change in oxidative stress), and pregnancy (19). Despite very limited evidence, the report suggested that vaping may be less harmful than smoking conventional cigarettes (19). Since the release of the NASEM report, new vaping devices and liquids have become available, and research on chronic health effects has grown considerably. The objective of this systematic review was to examine all relevant peer-reviewed literature published since the NASEM report and synthesize findings on vaping-related chronic health effects taking into consideration the volume and quality of evidence for key health outcomes.

## Methods

This review was prospectively registered on the Open Science Framework (https://osf.io/u9btp) (40) and adhered to the PRISMA statement (41). The review was initially designed to be a rapid one to provide a quick update on the NASEM report (19). However, it was converted to a full systematic review. Because of the sheer volume of research that focused on chronic health impacts of e-cigarette use, this review focuses on outcomes with the largest number of studies assessing the impact of daily e-cigarette use exposure, which was defined as daily vaping for at least 1 month.

### Study inclusion criteria

#### Population

All human *in-vivo* studies were eligible.

#### Exposure

The primary exposure was vaping. Vaping refers to e-cigarette use, including the use of e-liquids with or without nicotine and HNB tobacco products. Those who use e-cigarettes (vape) are referred to as “e-cigarette users”. E-cigarette use was assessed by level of exposure to include the following groups: (1) “daily e-cigarette users,” defined as those who have vaped every day for at 1 month (≥4 weeks) or who reported daily use; (2) “occasional e-cigarette users,” defined as those who reported vaping for <1 month or who reported occasional use; (3) “unclear e-cigarette users,” defined as those who were “ever users” (e.g., asking participants if they had vaped in the past year or month, or simply asking them if they have ever vaped) or with no description of duration of use or definition of what constitutes a “vaper.” If a study combined both daily and occasional e-cigarette users in the same group, the users are summarized as occasional users. Additionally, to reduce potential confounding due to cigarette exposure, the daily and occasional e-cigarette users groups were further stratified by former smoking status with “exclusive e-cigarette users,” defined as those with no history of conventional tobacco cigarette smoking. Studies examining the effects of second-hand smoke exposure, withdrawal symptoms, effects of different doses of nicotine, and effects of cannabis and THC, and, smoking cessation studies that did not include the health effects of vaping as principal outcome were excluded.

#### Comparators

Health effects among e-cigarette users were compared to “non-smokers” (no reported e-cigarette use or conventional tobacco cigarette use), “traditional smokers” (current conventional tobacco cigarette use), and “dual-users” (defined as those who use both e-cigarettes daily occasionally or of no clear duration and conventional tobacco cigarettes).

#### Outcomes

Chronic physical health effects were selected based on the research available comparing daily e-cigarette users to at least one comparison group. Studies on acute, transient changes (e.g., change in blood pressure 1 h after vaping) and withdrawal symptoms were excluded. Acute effects referred to those that developed suddenly and lasted for a short period (e.g., few days). Chronic effects were those that developed more slowly and present over an extended period of time (e.g., established disease). Studies that concentrated on e-cigarette or vaping use-associated lung injury (EVALI), mental health outcomes, sleep duration, or behavioral outcomes, as well as biomarkers of exposure to chemicals/metals in body fluid, were excluded.

#### Study designs

Quantitative studies were eligible, including cross-sectional, cohort, case control, and randomized controlled trials (RCTs). Experimental studies that examined the effects of switching from combustible tobacco cigarettes to vaping (e.g., smoking cessation modality) were eligible provided they also reported health effects as their primary outcome after at least 1 month of use. If the duration of use was less than once a month, baseline outcomes were used, and the study was categorized as cross-sectional. Literature/systematic reviews, dissertations, case reports, case series, qualitative studies, and *in vitro* and animal studies were also excluded.

#### Publication status and language

Studies were restricted to those published and indexed in the peer review literature in English or French. Only literature indexed between 31 August 2017 and 29 January 2021 was considered for this review. Conference publications were excluded.

### Search strategy

Research librarians were consulted, and the search strategy of the NASEM report was used with the addition of a new “vaping” MESH subject heading available in the MEDLINE database (i.e., vaping/). The following three databases were searched on 20 September 2019 with an update on 19 January 2021: Ovid MEDLINE, Ovid PsycINFO, and Ovid EMBASE. The search strategy used is shown in Supplementary Tables S1-1–S1-3). Gray literature was excluded from this systematic review.

### Study selection and data extraction

Following the search, titles and abstracts of studies were imported into RefWorks (RefWorks, Bethesda, MD, United States), and duplicates were removed using the “dedupe” function. The titles and abstracts were then imported into Covidence (Veritas Health Innovation, Melbourne, Australia), where additional duplicates were removed. Two independent reviewers screened all the titles and abstracts to identify potentially relevant studies. Conflicts were discussed and resolved by discussion and consensus. If a consensus could not be reached, a third reviewer was consulted. The full texts of all potentially relevant studies were then independently screened by two reviewers. Again, remaining discrepancies were discussed with a third reviewer. Systematic reviews were hand-searched for additional studies. Standardized data extraction forms were developed and completed using Google forms. All extracted data were verified by a second reviewer for accuracy.

### Risk of bias appraisal and conflict of interest

The included studies were assessed for risk of bias (RoB) using a modified Critical Appraisals Skills Programme (CASP) Checklist specific for each study design (42). The CASP checklist tools assess measurement bias, recruitment bias, confounding factors, precision, and external generalizability. An additional item assessed for possible conflict of interest if funding or contributions to support the study were received from the tobacco/e-cigarette industry or the pharmaceutical industry.

For cross-sectional studies, questions assessing the completeness and length of cohort follow-ups were removed. Furthermore, CASP items assessing whether the studies fit with the existing literature were removed, as the intention of this review was to examine and synthesize the evidence. A study was considered with high RoB if one or more of the items that assessed the “validity of the results” on the CASP checklist was not met (Supplementary Tables S4-1–S4-4).

### Data synthesis

Because of heterogeneity in the measures used to assess specific outcomes, findings were summarized using a narrative synthesis approach. Narrative syntheses describe the certainty of evidence and overall direction of associations between e-cigarette users and health outcomes relative to study comparison groups (non-smokers, cigarette smokers, and dual-users). The direction of association was determined by “vote counting,” where it summarized the number of studies per health outcome that reported “better,” “poorer,” or “null” associations with e-cigarette users compared to non-smokers, dual-users, and cigarette smokers. In the vote counting table, the studies were stratified by e-cigarette use exposure (daily, occasional, or unclear duration) and study design (RCT, cohort, pre-post, case-control, and cross-sectional). Results for the sub-analysis of studies that included “exclusive e-cigarette users” (i.e., no history of cigarette smoking) are also provided.

The narrative synthesis summarizes the findings only for daily e-cigarette users. It does not summarize the findings for occasional users or if the duration of e-cigarette use was unclear because of increased level of exposure uncertainty within the groups. The results for exclusive e-cigarette users or daily users were highlighted, when available. If a study reported on both daily and occasional e-cigarette use, only findings for daily users were included in the vote counting. If two studies analyzed the same outcome from the same survey (cohort), the one with most recent publication date was included unless the older one used a larger sample size from the cohort (e.g., pooling two waves of data collection). In order to minimize over-counting of studies reporting on multiple related indicators or biomarkers, the overall direction of association was based on the direction of at least 60% of the findings. Two vote counts were given in cases when there was a 50% split in the direction of associations, or three vote counts for a 33% split. Footnotes in the vote-counting tables identify study results contributing to more than one vote count.

The overall certainty of evidence by outcome was assessed using a modified Grading of Recommendations Assessment, Development and Evaluation (GRADE) approach, adapted for narrative syntheses (43, 44), and was summarized based on the highest level of study design (i.e., RCTs before cross-sectional studies). Supplementary Table S5 provides a summary of judgment decision rules applied in GRADE. The certainty of evidence was assessed for all studies that reported on findings for daily e-cigarette users and exclusive e-cigarette users separately, and was further stratified by study design (i.e., RCTs, pre-post and cohort studies were considered of higher quality than cross-sectional and case-control studies).

## Results

### Description of studies

**Figure 1:**
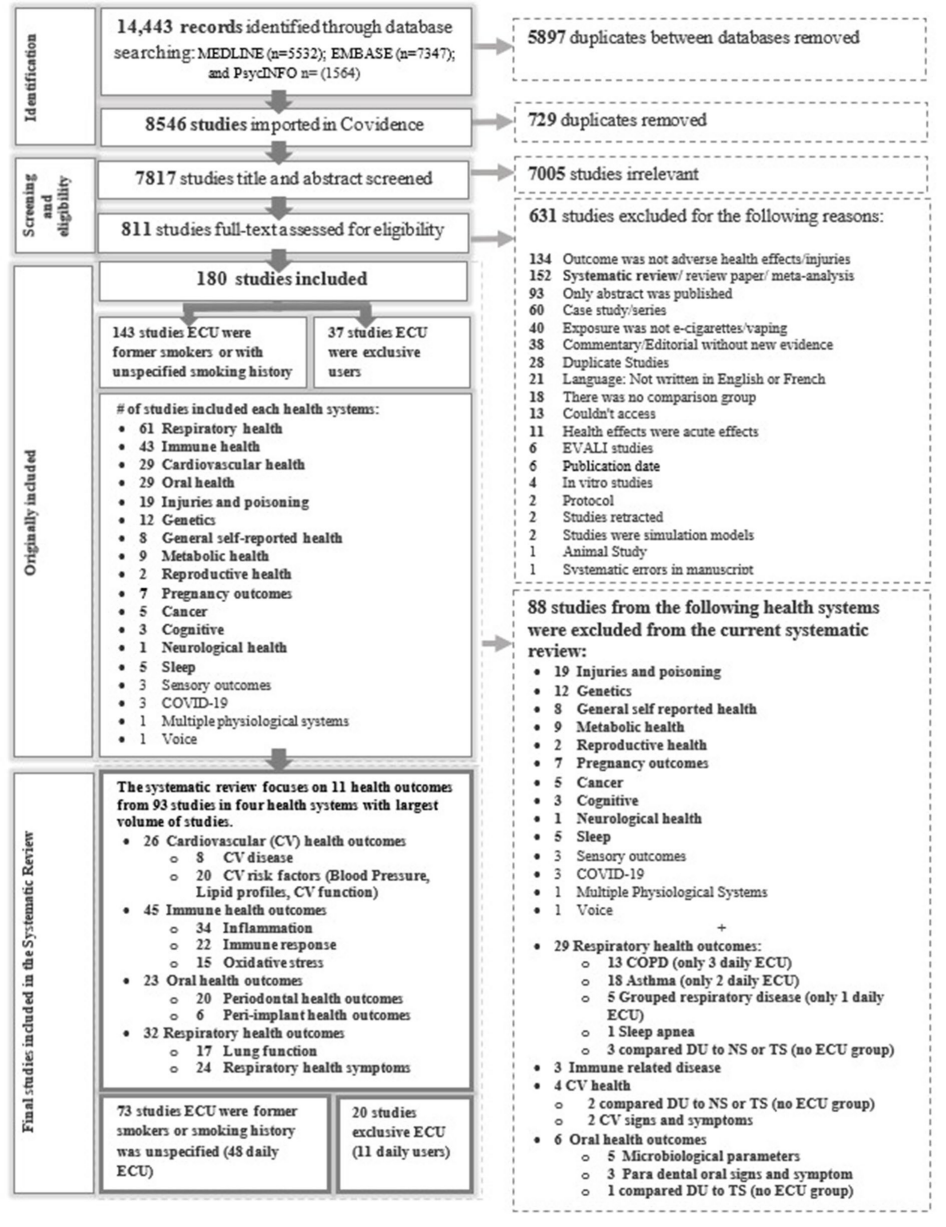
The PRISMA diagram.

Figure 1 displays the screening process and reasons for exclusion in each stage. The database search identified 14,443 potentially relevant studies; of these, 180 met the inclusion criteria. Supplementary Tables S2-1–S2-4 provide the study characteristics. Supplementary Tables S3-1–S3-4 provide the findings from each study. Supplementary Tables S4-1–S4-4 summarize the RoB assessments for daily e-cigarette users. Outcome measures in Supplementary materials presents a list of combined outcomes included in the systematic reviews, and Supplementary material [Full-texts reviewed studies and reasons for studies exclusion (excel file)] provides the reason for exclusion. Findings are summarized for 93 studies that reported on one or more of the 11 most common outcomes (Table 1) and Supplementary Tables in Supplements S2, S3. Fifty-nine studies assessed daily e-cigarette users, but only 11 reported on exclusive e-cigarette users. Thirty-four percent (*n* = 32) of the studies assessed outcomes from more than one health domain (e.g., respiratory and cardiovascular (CV) or respiratory and oral). Moreover, many studies assessed several outcomes within a health domain (e.g., cardiovascular disease (CVD), blood pressure, and lipids profiles; Table 1).

**Table 1:** Number of studies^1^ by physiological system and health outcome of daily users, and sub-set summary for exclusive e-cigarette users stratified by study design.

**System**	**Outcome**		**All studies**								**Exclusive e-cigarette usestudies only**							
			**Any exposure**		**Daily e-cigarette use only**						**Any exposure**		**Daily e-cigarette use only**					
					**Total**		**RCT/ Pre-post**	**Cohort**	**Case-control**	**Cross-sectional**			**Total**		**RCT/ Pre-post**	**Cohort**	**Case-control**	**Cross-sectional**
Cardiovascular	Cardiovascular disease		26	8	19	5	0	0	0	5	3	3	2	2	0	0	0	2
	CV risk factors																	
	Blood pressure			14		8	4	0	0	4		0		0	0	0	0	0
	Biomarkers of lipids			8		6	3	0	0	3		0		0	0	0	0	0
	CV function			8		6	1	0	0	5		0		0	0	0	0	0
Immunological	Inflammation		45	34	33	26	5	0	1	20	6	5	5	4	1	0	0	3
	Immune response			22		17	5	0	1	11		4		3	1	0	0	2
	Oxidative stress			15		8	3	0	1	4		1		0	0	0	0	0
Oral	Periodontal		23	20	19	15	0	2	2	11	5	4	5	4	0	1	0	3
	Peri-implant			6		6	0	0	0	6		1		1	0	0	0	1
Respiratory	Lung function		32	17	15	13	4	3	0	6	8	3	2	2	0	1	0	1
	Respiratory symptoms			24		10	2	3	0	5		6		1	0	1	0	0
Total number of studies			92		59^3^		7	5	2	45	19		11		1	1	0	9

^1^The total number of studies is not merely an addition of articles in each outcome, as some assessed several outcomes.

^2^The findings in the vote-counting table (Table 2) were drawn from 93 studies (73 for former smoking were not specified and 20 were for exclusive e-cigarette use).

^3^The narrative synthesis and certainty of evidence summarized in Table 3 focused on daily e-cigarette use (n = 59 studies, of which 11 were on exclusive e-cigarette use).

From the 93 included studies, the majority (78%, *n* = 71) used a cross-sectional design with very few cohort (9%, *n* = 9), RCTs (8.4%, *n* = 8), quasi-experimental (2%, *n* = 2), and case-control studies (2%, *n* = 2). For the majority (79%, *n* = 72) of the studies, the former smoking status of e-cigarette users was not reported. Table 1 presents the number of studies for each health outcome stratified by study design, daily exposure, and whether e-cigarette use was exclusive (i.e., daily e-cigarette users that never smoked conventional tobacco cigarettes). Half of the studies were conducted in the US (53.8%, *n* =4 9), with fewer from Middle Eastern/Arab countries (19.7%, *n* = 18), European countries (14.2%, *n* = 13), and eastern Asia (4.3%, *n* = 4). Most of the study populations included adults. Only three (69–71) included samples from youth. Fifty-nine studies (65%) reported on daily e-cigarette users, but only 11 of (19%) were on exclusive e-cigarette users (i.e., had never smoked cigarettes). These studies are the focus of health outcomes in the narrative synthesis section below.

### Risk of bias and conflicts of interest

The RoB among the studies reviewed is summarized in Supplementary Tables S4-1–S4-4. For all the 11 outcomes assessed, studies examining daily e-cigarette users had a high RoB, which contributed to reduction in the certainty of evidence. All the RCTs (*n* = 7) were considered to have a high RoB largely because of recruitment bias and confounding factors. Most of the RCTs (71%), had no (*n* = 2) or unclear (*n* = 3) randomization in the assignment of the e-cigarette users to the control groups. In all the RCTs (*n*=7), patients, health workers, or study personnel were not blind to treatment (i.e., e-cigarette use) (*n* = 5) or it was unclear (*n* = 2) whether there was blinding. For most of the RCTs (86%), the groups were not (*n* = 4) or it was unclear (*n* = 3) if they were similar at the start of the trial.

In the cohort studies (*n* = 5), 80% (*n* = 3) had a high risk of selection bias (i.e., due to convenience samples), and all of them had a high RoB due to confounding factors. For the majority (83%), attrition was not a problem, and all the studies had a reasonable follow-up period (min = 6 months, max = 60 months, and average = 33.6 months). In the cross-sectional studies (*n* = 45), the majority (77%) had a high risk of selection bias and bias in 80% of the studies due to lack of controlling for confounding factors (e.g., duration of e-cigarette use, and smoking).

Twenty percent of the studies examining daily e-cigarette use (*n* = 12/59) had a potential conflict of interest (COI) related to direct or indirect involvement of the tobacco or e-cigarette or pharmaceutical industry. A large proportion of the more rigorously designed studies had a potential COI; 62% (5/8) of the RCTs and 33% (3/9) the cohort studies compared to the 18% (13/71) of the cross-sectional studies (Supplementary Tables S2.1–S2.4).

### Health outcomes: Narrative synthesis

Table 1 summarizes the total number of studies for each outcome and those that reported on daily or exclusive e-cigarette use stratified by study design. Many of the studies assessed more than one outcome. Supplementary Table S2 provides all the outcomes assessed in each study and provides details on population characteristics, and Outcome measures in Supplementary materials provides all lists of combined outcomes. Table 2 provides the vote-count by outcome based on the statistical significance and directionality of effect for comparisons (i.e., e-cigarette users compared to non-smokers, cigarette smokers, or dual-users). The vote counting reflects the number of comparisons in each group. A sub-summary of the results for exclusive e-cigarette users is provided, although very few studies examined the health outcomes of exclusive e-cigarette use. In total, 59 studies assessed daily e-cigarette users related to CV (*n* = 19), immunological (*n* = 33), oral (*n* = 19), and respiratory (*n* = 15) health outcomes. Hereafter we focus our narrative synthesis on these 59 studies.

**Table 2:** Vote counting for all the studies (including those on exclusive e-cigarette use) and a sub-analysis for exclusive e-cigarette use only.

**Health outcome categorized by frequency of e-cigarette use and study design**	**E-cigarette users (ECU)vs. non-smokers(NS)**						**ECU vs. traditional smokers (TS)**						**ECU vs. dual-users (DU)**					
	**All studies**			**Exclusive** ^**1**^ **ECU (sub-analysis)**			**All studies**			**Exclusive ECU (sub-analysis)**			**All studies**			**Exclusive ECU (sub-analysis)**		
	**Better**	**NS**	**Poorer**	**Better**	**NS**	**Poorer**	**Better**	**NS**	**Poorer**	**Better**	**NS**	**Poorer**	**Better**	**NS**	**Poorer**	**Better**	**NS**	**Poorer**
**CARDIOVASCULAR HEALTH**																		
**Cardiovascular diseases (Coronary heart disease and stroke)**																		
**Daily users**																		
Cross-sectional		4^2^	1		2^2^													
**Occasional user**			
Cross-sectional		2			1													
**Cardiovascular risk factors**																		
**Blood pressure**																		
**Daily users**																		
RCT		2						3										
Cross-sectional		3						3						1				
**Occasional users**																		
RCT								2						1				
Pre-post							1^3^	1^3^										
Cross-sectional		2						1						1				
**Biomarkers for lipids**																		
**Daily users**																		
RCT		2					1	2										
Cross-sectional		2					1^4^	3^4^										
**Occasional users**																		
Cross-sectional		1^5^	1^5^															
**Cardiovascular function**																		
**Daily users**																		
RCT							1											
Cross-sectional		3	1					5^6^	1^6^					1				
**Occasional users**																		
RCT								1										
pre-post								1										
**IMMUNOLOGICAL HEALTH**																		
**Inflammation**																		
**Daily users**																		
RCT		2	1		1		1	2										
Cross-sectional		9	11		2	1	6	6		1								
Case-control			1				1											
**Occasional users**																		
RCT																		
Cross-sectional								1						1				
**Unclear**																		
Cross-sectional		4	2		1			2						2				
**Immune response**																		
**Daily users**																		
RCT		4^7^	3^7^		1		2^8^	3^8^										
Cross-sectional		7^9^	5^9^		1	1	5^10^	7^10**, 11**^	1^11^									
**Occasional users**																		
RCT								1						1				
Pre-post								1										
**Unclear**																		
		2^12^	1^12^					^1^						^1^				
**Oxidative stress**																		
**Daily users**																		
RCT		2					3											
Case-control		1^13^	1^13^															
Cross sectional		2	1				1	2										
**Occasional users**																		
Pre-post							1											
Cross-sectional		1					1	1										
**Unclear**																		
Cross-sectional	1	1	2		1			2					1					
**ORAL HEALTH**																		
**Periodontal health**																		
**Daily users**																		
Cohort		1	1		1			1										
Case-control		1						2										
Cross-sectional	2	6^14^	4^14^	1	2^14^	1^14^	2^14^	2^14^		2^14^	1^14^							
**Occasional users**			
Cross-sectional			2															
**Unclear**			
Cohort								1						1				
Cross-sectional			1															
**Peri-implant oral health**																		
**Daily users**																		
Cross-sectional	4 ^15, 16, 17^	3^16, 17^	6^15, 16, 17^			1	3^16, 18^	4^16, 18^	1^16, 18^	1^18^	1^18^							
**RESPIRATORY HEALTH**																		
**Lung function**																		
**Daily users**																		
RCT		2					1	4							1			
Cohort		1			1		1	1										
Cross-sectional	1^19^	4^19^	3^19^			1		4										
**Occasional users**																		
Cohort		2			2^20^													
**Unclear**																		
Cross-sectional		2			1													
**Respiratory symptoms**																		
**Daily users**																		
RCT							1	1						1				
Cohort		1			1		2^21^	1^21^										
Cross-sectional		3	1				1	2	1				1					
**Occasional users**																		
Cohort		1	2			1												
Cross-sectional		4^22^	5^22^		5		1			1				1				
**Unclear**																		
Cross-sectional		2	1															

^1^Exclusive e-cig users (ECUs) are those without any lifetime history of cigarette use.

^2^Vinhyal et al. (45) found a significant increase in odds of having myocardial infarction, but there was no statistically significant increased odds of stroke or coronary heart disease.

^3^Ikonomidis et al. (46) reported a significant decrease in systolic blood pressure but not in diastolic blood pressure. As a result, of the 50/50 split, we added a vote for both poorer and non-significant outcomes.

^4^Sakaguchi et al. (47) reported significantly better HDL and triglyceride values when comparing daily ECU to TS, and non-significant differences in total cholesterol and LDL. As a result of the 50/50 split, we added a vote for both better and non-significant outcomes.

^5^Kim et al. (48) reported that ECUs have significant higher odds of having high triglycerides but that that there were an insignificant difference in odds of having low HDL cholesterol. As a result of the 50/50 split, we added a vote for both poorer and non-significant outcomes.

^6^Podzolkov et al. (49) reported significant higher albuminuria levels and non-significant differences in augmentation index when comparing ECU to TS. We split the vote to account for the 50/50 difference.

^7^Haziza et al. (50) found that WBC count was significantly higher in ECUs than in NSs and an insignificant difference in 11-dehydro thromboxane B2 between ECUs and NSs, resulting in a 50/50 split in vote counting. Therefore, the vote was counted twice, once for insignificant and once for poorer outcome.

^8^Ludicke et al. (51) and Ludicke et al. (52) assessed 11-dehydro thromboxane B2 and WBC counts as immune response indicators. Both studies found significantly higher WBC counts among TSs than among ECUs and an insignificant difference in 11-dehydro thromboxane B2 between TSs and ECUs, resulting in a 50/50 split in vote counting.

^9^Cichonska et al. (53) found that lysozyme was significantly lower among ECUs than among NSs and that there was no difference in lactoferrin between ECUs and NSs, resulting in a 50/50 split in vote counting. Ghosh et al. (54) found that proteases (neutrophil elastase, MMP-2, and MMP-9) were significantly higher among ECUs than among NSs, and there was no difference in protease inhibitors (A1AT, SLPI, TIMP-1, and TIMP-2) between ECUs and NSs, resulting in a 50/50 split in vote counting. Jackson et al. (55) found that IgE was significantly higher among ECUs than among NS, but that IgG did not differ significantly between the ECUs and NSs, resulting in a 50/50 split in vote counting.

^10^Cichonska et al. (53) found that lactoferrin was significantly higher among ECUs than among TS, and that there was no difference in lysozyme level between the ECUs and the TSs, resulting in a 50/50 split in vote counting. Oliveri et al. (56) assessed white blood cell (WBC) counts and 11-dehydrothromboxane B2 as immune response indicators. The WBC counts did not differ significantly between the ECUs and the TSs, but 11-dehydrothromboxane B2 was significantly lower among the ECU than among the cigarette smokers, resulting in a 50/50 split in vote counting. Song (57) found that the numbers of macrophages and lymphocytes were significantly lower among the vapers than the TSs and that the number of neutrophils and eosinophils did not differ significantly between the ECU and the TSs, resulting in a 50/50 split in vote counting.

^11^Reidel et al. (58) assessed mucin proteins, neutrophil counts, neutrophil granule proteins, and airway epithelial defense proteins as immune response indicators. Seven out of the 13 indicators did not differ significantly between ECUs and TSs, and 6 of the 13 indicators were associated with poorer outcomes among the ECUs than among the cigarette smokers, resulting in approximately a 50/50 split in vote counting.

^12^Singh et al. (59) assessed the levels of growth factors, desmosine, and PAI-1 as immune response indicators. Seven of 13 indicators (EGF, VEGF, b-NGF, PDGF, SCF, HGF, and PIGF) were significantly higher among the ECUs than among the NSs, and 6 of the 13 indicators (BDNF, BMP-2, TGC-alpha, bFGF/FGF2, desmosine, and PAI-1) did not differ significantly between the ECUs and NSs, resulting in a 50/50 split in vote counting.

^13^Karaaslan et al. (60) assessed GSH-Px and 8–OhdG as indicators of oxidative stress. GSH-Px was significantly high among ECUs compared to NSs, and there was no difference in 8-OHdG between the ECUs and NSs, resulting in a 50/50 split in vote counting. Therefore, the vote was counted twice, once for poorer and once for non-significant outcome.

^14^Jayed et al. (61), found no significant difference in clinical periodontal parameters between ECUs and smokers and NS, but there were statistically significant better outcomes compared to TSs and poorer outcomes compared to NSs in self-reported gingival health, resulting in a 50/50 split in vote counting.

^15^Al-Aali et al. (62) found that clinical peri-implant assessment for PD and PI were significantly higher in ECUs than in NSs, but that BoP and radiographic evaluations for peri-implant bone loss (PIBL) were significantly lower in the ECUs than in the NSs, resulting in a 50/50 split in vote counting.

^16^AlDeeb et al. (63) found significantly lower BOP, higher BD, and no significant difference in PI between ECUs and NSs, and significantly higher BOP, lower PI, and no significance difference in PD when the ECUS were compared to TSs, resulting in a 33% split in vote counting for each comparison group.

^17^Sinha et al. (64) found no significant difference in PI, significantly better BOP, and significantly poorer PD and PIBL in ECUs compared to NSs; hence, the vote count was spilt into 3.

^18^AlQahtani et al. (65) found no statistical significant association between in BOP and PI between ECUs and TSs, but significantly lower PD and bone loss (RBL) in ECUs than in TSs, resulting in a 50/50 split in vote counting.

^19^Kizhakke et al. (66) found no significant difference in mean alveolar ventilation, mean alveolar perfusion, oxygen saturation, peripheral oxygen saturation, and FVC, significantly better FEV 1 and FEV1/FVC, but worse perfusion heterogeneity and ventilation-perfusion heterogeneity in ECUs compared to NSs, resulting in a 33% split in vote counting.

^20^Bowler et al. (67) examined the lung function in two different cohorts and thus was counted as 2 votes.

^21^Polosa et al. (68) used a sample of patients with COPD patients and measured the number of COPD exacerbations, and CAT score, which is a summative measure of COPD symptoms; both of which were categorized into respiratory symptoms. When comparing ECUs with TSs, the study found that the ECUs had significantly less COPD exacerbations, but that there was no significant difference in CAT, compared to traditional TSs. This resulted in a single vote in both categories in both comparator groups.

^22^Alnajem et al. (69) measured wheezing independently, as well as including a grouped measure of respiratory symptoms. Wheezing was found to be significant, but grouped symptoms was not, resulting in a 50/50 split in vote counting; therefore, the vote was counted twice to account for this.

### Certainty of evidence

The certainty of the evidence for all the health outcomes was very low regardless of daily or exclusive e-cigarette use. High RoB and study design were key contributor to reducing the certainty of evidence. Table 3 provides the certainty of evidence for each outcome separately for high and low quality study designs. Results are reported separately for daily and exclusive e-cigarette users. The Table summarizes the relative direction of association and certainty of the evidence. The outcome descriptions summarize the highest quality of evidence available with an emphasis on daily and exclusive e-cigarette use and study design.

**Table 3:** Summary of findings for all daily ECU studies and exclusive ECU studies by study design.

**Outcome**	**Group^1^**	**ECU exposure**	**Study design**	**Number of studies**	**Sample size**	**Relative effect^2^**	**Certainty of evidence**
**Cardiovascular health**							
CV disease	ECUvs. NS	Exclusive	Cross-sectional	2	380,644	No statistically significant difference One study found, however,variationby CVD outcome, with a significantly higher odds of myocardial infraction, but not stroke or coronary heart disease	**Very low** 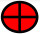 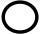 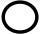 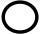 Initial rating: low RoB: −1, Inconsistency: 0, indirectness: 0, imprecision: 0, publication bias: 0
	ECU vs. NS	All	Cross-sectional	5	493,064	No statistically significant difference (4/5 of the studies)	**Very low** 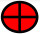 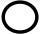 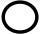 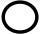 Initial rating: low RoB: −1.5, inconsistency: 0, indirectness: 0, imprecision: 0, publication bias: 0
**Cardiovascular risk factors (No studies for exclusive ECU)**							
Blood pressure	ECU vs. NS	All	RCT	3	237	No statistically significant difference	**Very low** 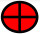 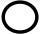 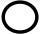 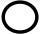 Initial rating: high RoB: −1.5, inconsistency: 0, indirectness: −0.5, imprecision: −1, publication bias: −1
	ECU vs. NS	All	Cross-sectional	3	376	No statistically significant difference	**Very low** 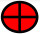 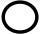 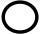 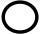 Initial rating: low RoB: −1.5, inconsistency: 0, indirectness: −0.5, imprecision: −1, publication bias: −1
	ECU vs. TS	All	RCT	4	335	No statistically significant difference	**Very low** 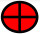 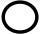 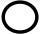 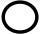 Initial rating: high RoB: −1.5, inconsistency: 0, indirectness: −0.5, imprecision: −1, publication bias: −1
	ECU vs TS	All	Cross- sectional	3	510	No statistically significant difference	**Very low** 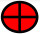 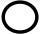 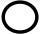 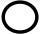 Initial rating: low RoB: −1.5, inconsistency: 0, indirectness: −0.5, imprecision: −1, publication bias: −1,
	ECU vs DU	All	Cross- sectional	1	88	No statistically significant difference	**Very low** 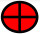 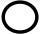 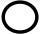 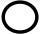 Initial rating: low RoB: −1.5, inconsistency: −1, indirectness: −0.5, imprecision: −1, publication bias: −1
Lipid biomarkers	ECU vs NS	All	RCT	2	237	No statistically significant difference	**Very low** 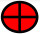 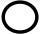 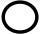 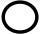 Initial rating: high RoB: −1.5, inconsistency: 0, indirectness: −0.5, imprecision: −1, publication bias: −1
	ECU vs NS	All	Cross- sectional	2	451	No statistically significant difference	**Very low** 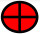 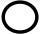 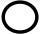 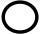 Initial rating: low RoB: −1.5, inconsistency: 0, indirectness: −0.5, imprecision: −1, publication bias: −1
	ECU vs TS	All	RCT	3	914	**Mixed findings:** No statistically significant difference (2/3 of the studies). The 3^rd^study showedbetter HDL and overall lipid profile in ECU	**Very low** 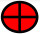 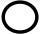 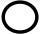 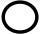 Initial rating: high RoB: −1.5, inconsistency: 0, indirectness: −0.5, imprecision: −1, publication bias: −1
	ECU vs TS	All	Cross- sectional	3	642	**Mixed findings:** No statistically significant difference (2/3 of the studies). The 3^rd^ study, reported significant higher odds of high triglycerides and HDL-cholesterol (c), but insignificant difference in LDL-c	**Very low** 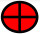 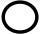 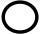 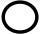 Initial rating: low RoB: −1.5, inconsistency: 0, indirectness: −0.5, imprecision: −1, publication bias: −1
Cardiovascular function	ECU vs NS	All	Cross- sectional	4	553	No statistically significant difference (3/4 of the studies)	**Very low** 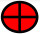 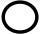 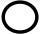 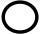 Initial rating: low RoB: −1.5, inconsistency: 0, indirectness: −0.5, imprecision: −1, publication bias: −1
	ECU vs TS	All	RCT	1	40	Statisticallysignificant betterCV function inECU	**Very low** 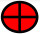 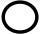 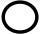 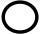 Initial rating: high RoB: −1.5, inconsistency: −1, indirectness: −0.5, imprecision: −1, publication bias: −1
	ECU vs TS	All	Cross- sectional	5	411	No statistically significant difference (4/5 of the studies)	**Very low** 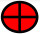 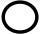 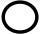 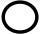 Initial rating: low RoB: −1.5, inconsistency: −1, indirectness: −0.5, imprecision: −1, publication bias: −1
	ECU vs DU	All	Cross- sectional	1	88	No statistically significant difference	**Very low** 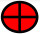 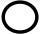 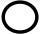 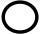 Initial rating: low RoB: −1.5, inconsistency: −1, indirectness: −0.5, imprecision: −1, publication bias: −1
Immunological health							
Inflammation	ECU vs NS	Exclusive	Cohort	1	27	No statistically significant difference	**Very low** 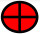 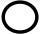 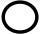 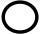 Initial rating: high RoB: −1.5, inconsistency: −1, indirectness: −0.5, imprecision: −1, publication bias: −1
	ECU vs NS	All	Cohort	4	299	No statistically significant difference (3/4 of the studies)	**Very low** 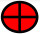 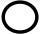 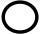 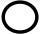 Initial rating: high RoB: −1.5, inconsistency: 0, indirectness: −0.5, imprecision: −1, publication bias: −1
	ECU vs NS	Exclusive	Cross sectional	3	195	**Mixed findings:** No statistically significant difference (2/3 of the studies), and statistically significant poorer biomarkers in ECU in the 3^rd^ study	**Very low** 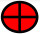 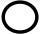 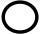 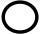 Initial rating: low RoB: −1.5, inconsistency: −1, indirectness: −0.5, imprecision: −1, publication bias: −1
	ECU vs NS	All	Cross- sectional	20	1549	**Mixed findings:** No statistically significant difference in 40% of the studies (8/20), andstatistically significant poorer biomarkers in 60%(12/20) of the studies	**Very low** 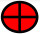 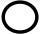 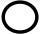 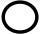 Initial rating: low RoB: −1.5, inconsistency: −1, indirectness: −0.5, imprecision: −1, publication bias: −1
	ECU vs TS	All	Cohort	3	914	No statistically significant difference (2/3 of the studies). Poorer biomarkers of inflammation in ECU in the 3^rd^ study	**Very low** 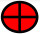 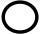 Initial rating: high RoB: −1.5, inconsistency: −1, indirectness: −0.5, imprecision: −1, publication bias: −1
	ECU vs TS	Exclusive	Cross- sectional	1	151	No statistically significant difference	**Very low** 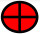 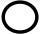 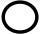 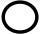 Initial rating: low RoB: −1.5, inconsistency: −1, indirectness: −0.5, imprecision: −1publication bias: −1
		All	Cross- sectional	13	1152	**Mixed findings**: No statistically significant difference in 54% (7/13) of the studies and statistically significant better biomarkers in 46% of the studies (*n* = 6/13)	**Very low** 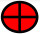 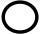 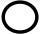 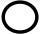 Initial rating: low RoB: −1.5, inconsistency: −1, indirectness: −0.5, imprecision: −1publication bias: −1
Immune response	ECU vs NS	Exclusive	RCT	1	27	No statistically significant difference	**Very low** 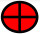 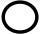 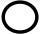 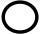 Initial rating: high RoB: −1.5, inconsistency: −1, indirectness: −0.5, imprecision: −1, publication bias: −1
		All	RCT	5	299	**Mixed findings**: No statistically significant difference(2/3 of the studies)	**Very low** 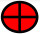 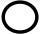 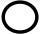 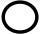 Initial rating: high RoB: −1.5, inconsistency: −1, indirectness: −0.5, imprecision: −1, publication bias: −1
		Exclusive	Cross- sectional	2	97	No statistically significant differences (1.5/2 of the studies) One study showed insignificant differences and the other showed both statistically insignificant and poorer immune response in ECU	**Very low** 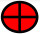 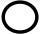 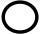 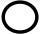 Initial rating: low RoB: −1.5, inconsistency: −1, indirectness: −0.5, imprecision: −1, publication bias: −1
		All	Cross- sectional	11	654	**Mixed findings**: No statistically significant difference, (7/11 of the studies)	**Very low** 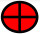 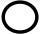 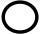 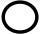 Initial rating: low RoB: −1.5, inconsistency: −1, indirectness: −0.5, imprecision: −1, publication bias: −1
	ECU vs TS	All	RCT	3	914	**Mixed findings**: No statistically significant differences (2/3 of the studies).	**Very low** 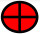 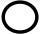 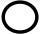 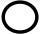 Initial rating: high RoB: −1.5, inconsistency: −1, indirectness: −0.5, imprecision: −1, publication bias: −1
			Cross-sectional	8	721	Statistically significant better immune response (7/8 of the studies, one of which also showed no significant difference)	**Very low** 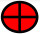 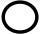 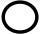 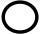 Initial rating: low RoB: −1.5, inconsistency: 0, indirectness: −0.5, imprecision: −1, publication bias: −1
Oxidative stress	ECU vs NS	All	Cohort	2	237	No statistically significant difference	**Very low** 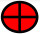 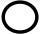 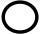 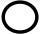 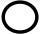 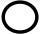 Initial rating: high RoB: −1.5, inconsistency: −1, indirectness: −0.5, imprecision: −1, publication bias: −1
	ECU vs NS	All	Cross- sectional	4	512	**Mixed findings**: No statistically significant difference in 2 studies and statistically significant poorer biomarkers in ECUin 2 studies	**Very low** 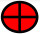 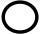 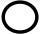 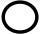 Initial rating: low RoB: −1.5, inconsistency: −1, indirectness: −0.5, imprecision: −1, publication bias: −1
	ECU vs TS	All	Cohort	3	914	**Mixed findings**: No statistically significant difference (2/3 of the studies)	**Very low** 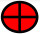 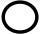 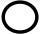 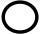 Initial rating: high RoB: −1.5, inconsistency: −1, indirectness: −0.5, imprecision: −1, publication bias: −1
	ECU vs TS	All	Cross- sectional	5	676	**Mixed findings**: Statistically significant better biomarkers in ECU (3/5 of the studies)	**Very low** 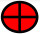 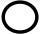 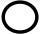 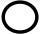 Initial rating: low RoB: −1.5, inconsistency: −1, indirectness: −0.5, imprecision: −1, publication bias: −1
**Oral health**
Periodontal	ECU vs NS	Exclusive	Cohort	1	59	No statistically significant differences in clinical parameters	**Very low** 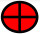 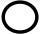 Initial rating: high RoB: −1.5, inconsistency: −1indirectness: −0.5, imprecision: −1publication bias: −1
		All	Cohort	2	10,020	**Mixed findings:** Statistically significant worse periodontal self-reported parameters (1 study), and no statistically significant difference in clinical parameters (1 study)	**Very low** 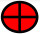 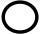 Initial rating: high RoB: −1.5, inconsistency: −1, indirectness: −0.5, imprecision: 0, publication bias: 0
	ECU vs NS	Exclusive	Cross-sectional	3	198	**Mixed findings**: Two studies showed no statistically significant difference in clinical parameters and showed mixed finding between worse and better parameters self reported outcomes	**Very low** 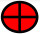 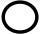 Initial rating: low RoB: −1.5, inconsistency: −1, indirectness: −0.5imprecision: −1publication bias: −1
		All	Cross-sectional	10	221,991	**Mixed findings**:No statistically significant difference in clinical parameters (6/10 studies), significant worse clinical parameters in ECU (3/10 studies), worse oral hygiene and self-reported complains (2/10) and better oral hygiene (2/10 studies)	**Very low** 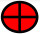 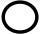 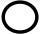 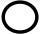 Initial rating: low RoB: −1.5, inconsistency: −1, indirectness: −0.5imprecision: 0publication bias: −1
	ECU vs TS	All/ Exclusive	Cohort	1	58	No statistically significant difference in clinical parameters	**Very low** 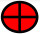 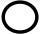 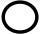 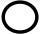 Initial rating: high RoB: −1.5, inconsistency: −1, indirectness: −0.5, imprecision: 0, publication bias: −1
		Exclusive	Cross-sectional	2	208	**Mixed findings**: Statistically significant better self-reported parameters in two studies, one of which also showed no statistically significant difference in clinical parameters	**Very low** 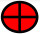 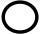 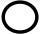 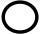 Initial rating: low RoB: −1.5, inconsistency: −1, indirectness: −0.5, imprecision: −1, publication bias: −1
		All	Cross-sectional	5	312	No statistically significant difference in clinical parameters in all studies (*n* = 4); better parameters (missing teeth) in two studies	**Very low** 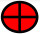 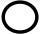 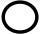 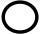 Initial rating: low RoB: −1.5, inconsistency: −1, indirectness: −0.5, imprecision: −1, publication bias: −1
Peri-implant	ECU vs NS	Exclusive	Cross-sectional	1	80	No statistically significant difference in clinical parameters	**Very low** 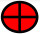 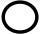 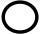 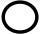 Initial rating: low RoB: −1.5, inconsistency: −1, indirectness: −0.5, imprecision: −1, publication bias: –
		All	Cross- sectional	6	442	**Mixed findings:** 30% of the periodontal parameters around the implants showing no significant differences, 30% showing statistically significant better outcomes and 40% showing statistically significant worse outcomes.	**Very low** 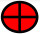 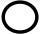 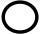 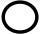 Initial rating: low RoB: −1.5, inconsistency: −1, indirectness: −0.5, imprecision: −1, publication bias: −1
	ECU TS	Exclusive	Cross- sectional	1	80	**Mixed findings**: No statistically significant difference in BOP and PI, but statistically significantly better outcomes in PD and bone loss (RBL) in ECU	**Very low** 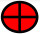 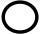 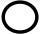 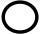 Initial rating: low RoB: −1.5, inconsistency: −1, indirectness: −0.5, imprecision: −1, publication bias: −1
		All	Cross- sectional	6	193	**Mixed findings:** 50% of the periodontal parameters around the implants showing No significant differences (50%), statistically significant better outcomes (33%) and statistically significant worse outcomes (17%)	**Very low** 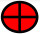 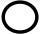 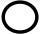 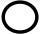 Initial rating: low RoB: −1.5, inconsistency: −1, indirectness: −0.5, imprecision: −1, publication bias: −1
**Respiratory health**							
Lung function	ECU vs NS	Exclusive	Cohort	1	21	No statistically significant difference	**Very low** 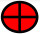 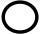 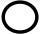 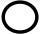 Initial rating: low RoB: −1.5, inconsistency: −1, indirectness: −0.5, imprecision: −1, publication bias: −1
		All	Cohort/ RCT	3	247	No statistically significant difference	**Very low** 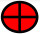 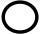 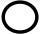 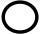 Initial rating: high RoB: −1.5, inconsistency: 0, indirectness: −0.5, imprecision: −1, publication bias: −1
		Exclusive	Cross-sectional	1	60	Statisticallysignifucantly worse results in lung function	**Very low** 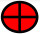 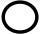 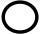 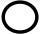 Initial rating: low RoB: −1.5, inconsistency: −1, indirectness: −0.5, imprecision: −1, publication bias: −1
		All	Cross sectional	6	228	No statistically significant difference (4/6 of the studies)	**Very low** 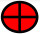 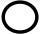 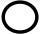 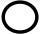 Initial rating: low RoB: −1.5, inconsistency: −1, indirectness: −0.5, imprecision: −1, publication bias: −1
	ECU vs TS	All	Cohort/ RCT	6	599	No statistically significant difference in 4/5 of the studies (80%), the other two showed better outcomes	**Very low** 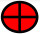 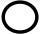 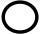 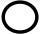 Initial rating: high RoB: −1.5, inconsistency: 0, indirectness: −0.5, imprecision: −1, publication bias: −1
		All	Cross-sectional	4	162	No statistically significant difference	**Very low** 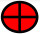 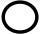 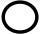 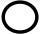 Initial rating: low RoB: −1.5, inconsistency: 0, indirectness: −0.5, imprecision: −1, publication bias: −1
	ECU vs DU	All	Cohort/ RCT	1	55	No statistically significant difference	**Very low** 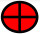 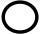 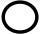 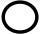 Initial rating: high RoB: −1.5, inconsistency: −1, indirectness: −0.5, imprecision: −1, publication bias: −1
Respiratory symptoms	ECU vs NS	Exclusive	Cohort	1	21	No statistically significant difference	**Very low** 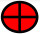 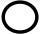 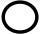 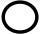 Initial rating: high RoB: −1.5, inconsistency: −1, indirectness: −0.5, imprecision: −1, publication bias: −1
	ECUvs NS	All	Cohort	1	21	No statistically significant difference	**Very low** 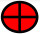 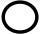 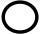 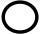 Initial rating: high RoB: −1.5, inconsistency: −1, indirectness: −0.5, imprecision: −1, publication bias: −1
		All	Cross-sectional	4	640	No statistically significant difference in 75% (3/4) of the studies	**Very low** 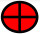 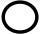 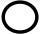 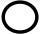 Initial rating: low RoB: −1.5, inconsistency: 0, indirectness: −0.5, imprecision: −1, publication bias: −1
	ECU vs TS	All	Cohort/ RCT	4	367	Statisticallysignifucant better respiratory symptoms in 75% (3/4) of the studies	**Very low** 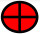 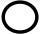 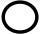 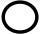 Initial rating: high RoB: −1.5, inconsistency: 0, indirectness: −0.5, imprecision: −1, publication bias: −1
		All	Cross-sectional	3	486	**Mixed findings**: no statistical signifucant difference in 2/3 of the studies), the 3^rd^showed better respiratory symptoms	**Very low** 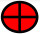 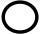 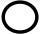 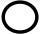 Initial rating: low RoB: −1.5, inconsistency: −1, indirectness: −0.5, imprecision: −1, publication bias: −1

1Groups are e-cigarette users (ECUs), non-smokers (NSs), traditional smokers (TSs), and dual users (DU).

^2^Direction of association is determined by the direction of 75% or more of studies, otherwise studies were considered as having mixed findings.

### Cardiovascular health

Twenty-five studies (45–52, 56, 72–88) explored cardiovascular (CV) health including cardiovascular disease (CVD) (*n* = 8), blood pressure (*n* = 14), biomarkers of lipid metabolism (*n* = 8), and CV function (*n* = 8). Nineteen studies assessed the association of daily e-cigarette use with CV health, five RCT (50–52, 73, 87), and 14 cross-sectional studies (45, 47, 49, 56, 74–83).

#### Cardiovascular disease

Eight studies (45, 48, 76, 78, 82, 83, 85, 86) assessed the odds of having CVD in e-cigarette users compared to non-smokers; five (45, 76, 78, 82, 83) concentrated on daily e-cigarette users, two of which assessed (45, 82) exclusive e-cigarette users. None compared daily e-cigarette users to cigarette smokers or dual-users. Indicators of CVD included self-reported premature CVD (i.e., disease age <65 years), myocardial infarction, ischemic attack (stroke), congestive heart failure, peripheral artery disease, and coronary heart disease. All evidence was cross-sectional and found no significant difference in the odds of CVD between daily e-cigarette users or exclusive e-cigarette users and non-smokers (45, 76, 78, 82, 83). One study looking at multiple CVD outcomes did find significantly higher odds of myocardial infarction for exclusive e-cigarette users compared to non-smokers, but there was no difference for stroke or coronary heart disease (76).

#### Blood pressure

Fourteen studies (46, 48, 50, 51, 72–75, 79, 80, 84, 86–88) assessed blood pressure, but only eight (50, 51, 73–75, 79, 80, 87) compared blood pressure results of daily e-cigarette users to those of non-smokers, smokers and/or dual users (four cross-sectional and four RCTs). None of the eight studies reported on exclusive e-cigarette use. Blood pressure indicators included measured effects on systolic, diastolic, and mean blood pressure or hypertension status. No significant difference was observed for daily e-cigarette users compared to non-smokers, cigarette smokers, or dual users.

#### Biomarkers of lipid metabolism

Eight studies (47, 50–52, 56, 77, 86, 88) assessed lipid profiles, six (47, 50–52, 56, 77) reported results for daily e-cigarette user, and none assessed exclusive e-cigarette use. Lipid biomarkers included total cholesterol, high-density lipoprotein cholesterol (HDL), low-density lipoprotein cholesterol (LDL), and/or triglycerides. The findings suggested no significant difference for any biomarker between daily e-cigarette users and non-smokers, regardless of study design. One of the three RCT studies (52), however, suggested better HDL at 6 months follow-up in daily e-cigarette users than in cigarette smokers. In contrast, two cross-sectional studies found no significant difference in lipids between daily e-cigarette users and cigarette smokers (56, 77), and one reported mixed findings suggesting better HDL and triglycerides in e-cigarette users but no significant differences in total cholesterol and LDL (47).

#### Cardiovascular function

Eight studies (46, 49, 72, 75, 79–81, 87) assessed CV function comparing e-cigarette users to non-smokers, smokers, or dual users. Six (49, 75, 79–81, 87) included daily e-cigarette users. None was conducted on exclusive e-cigarette users. A wide range of markers was explored, with the most common being differences in heart rate, pulse wave velocity, and arterial stiffness. Most (75%) of the studies found no statistically significant differences between daily e-cigarette users and non-smokers (79–81) or dual users (79). A single RCT (87) found that daily e-cigarette users had better CV function indicators than smokers. No differences were observed among the cross-sectional studies (49, 75, 79–81).

### Immunological health

Forty-five studies (46, 47, 50–58, 60, 62–65, 69, 72, 77, 80, 87, 89–112) examined inflammation, immune response, and oxidative stress, from which 33 (six RCTS (50–52, 87, 99, 100), two case controls (60, 111), and 25 cross-sectional studies (47, 53–58, 62–65, 77, 89–98, 101–103) included daily e-cigarette use. Only six studies (55, 64, 65, 98, 99, 103) included exclusive e-cigarette use.

#### Inflammation

Twenty-six studies compared biomarkers of inflammation between daily e-cigarette users and non-smokers and/or smokers (47, 50–53, 56–58, 60, 62–65, 89–100, 103). Most of these studies were related to oral health (*n* = 14) (53, 62, 63, 65, 89–94, 97, 103), followed by respiratory health (*n* = 7) (57, 58, 95, 96, 98–100), CV health (*n* = 4) (47, 50–52), and a single study with biomarkers of inflammation non-specific to a physiological system (56). Only four studies reported on exclusive e-cigarette use (65, 98, 99, 103). The biomarkers of inflammation included cytokine and protein biomarkers of inflammation, inflammatory mediators, anti-inflammatory lipid mediators, factors inhibiting inflammation, and other biomarkers of inflammation (e.g., soluble intercellular adhesion molecule-1 and gingival crevicular fluid volume. For a complete list of biomarkers, refer to Supplementary Table S2 and Outcome measures in Supplementary materials). The results differed based on the physiological system. Table 2 provides the direction of association for the three physiological systems combined (CV, respiratory, and oral health), and system-specific findings are outlined below.

For inflammation related to oral health, the majority of cross-sectional studies (79%, 11/14), one of which included exclusive e-cigarette use (65), found that inflammation was significantly higher in daily e-cigarette users than in non-smokers. Comparing daily e-cigarette users to smokers, only one study included exclusive e-cigarette users and found no statistically significant differences in inflammation (103). For the remaining studies, half reported significantly lower levels of inflammation among daily e-cigarette users than among cigarette smokers (60, 63, 92), and the other half reported no significant (53, 97, 103) associations. The inflammation biomarkers included gingival crevicular fluid volume and various cytokines.

For inflammation related to respiratory health, two RCTs (99, 100) reported on daily e-cigarette users, one of which reported on exclusive e-cigarette users (99), and neither found significant differences in inflammation compared to non-smokers. The other RCT (100) found significantly higher inflammation in daily e-cigarette users than in non-smokers. Four of the five cross-sectional studies comparing daily e-cigarette users to non-smokers found no significant differences in inflammation (57, 95, 96, 98), including one that looked at exclusive e-cigarette users (98). One study found significantly higher inflammation in daily e-cigarette users than in non-smokers (58). Respiratory health biomarkers were measured via sputum and serum for pulmonary health, other inflammatory biomarkers included nasal lavage fluid, nasal epithelial lining and bronchoalveolar. For a complete list of biomarkers, refer to Supplementary Table S2 and Outcome measures in Supplementary materials.

For inflammation related to CV health, both RCTs (50, 51) and a cross-sectional study (47) found no significant difference in inflammation between daily e-cigarette users and non-smokers; none were exclusive e-cigarette users. Comparing daily e-cigarette users to smokers, two RCTs (50, 51) reported no significant differences, while one RCT (51) and a cross-sectional study (47) found lower levels of inflammation among daily e-cigarette users. The biomarkers of inflammation included HS-C reactive protein, sICAM-1, fibrinogen, and biomarkers of platelet activation. For a complete list of biomarkers, refer to Supplementary Table S2 and Outcome measures in Supplementary materials.

#### Immune response

Seventeen studies (47, 50–58, 60, 77, 98–102) assessed immune response between daily e-cigarette users and non-smokers and/or smokers, of which three included exclusive e-cigarette users (55, 98, 99). The biomarkers of immune response included immune infiltration measures, white blood cell counts, platelet activity, and levels of growth factors, proteins, and enzymes associated with immune response. For a complete list of the biomarkers, refer to Supplementary Table S2 and Outcome measures in Supplementary materials. Seven studies explored indicators of immune response linked to respiratory health (54, 57, 58, 98–100, 102), four were related to CV health (47, 50–52), two were related to oral health (53, 60), and four were unrelated to a specific physiological system (55, 56, 77, 101). There were no clear patterns by immune response system, and the results were mixed for both RCT and cross-sectional study designs. Comparing exclusive e-cigarette users to non-smokers, one RCT (99) and a cross-sectional study (98) suggested no statistically significant difference in biomarkers of immune response related to respiratory health. This finding contradicted the RCT (100) results for daily e-cigarette users compared to non-smokers that found statistically significant lower levels for immune response biomarkers following inoculation with live attenuated influenza virus (100). Two out of the three RCTs (51, 52) and a cross-sectional study (47) comparing immune response related to CV health among daily e-cigarette users compared to non-smokers showed no significant differences, while the third RCT (50) showed non-significant differences in fibrinogen levels but higher white blood cells among daily e-cigarette users. A cross-sectional (53) and a case-control study (60) related to oral health (53, 60) and cross-sectional studies related to non-specific physiological system (55, 56, 77, 101) showed mixed findings with non-significant (53, 55, 56, 101), poorer (53, 60), and better (56, 77) immune response for daily e-cigarette users than for non-smokers.

No studies compared exclusive e-cigarette users to smokers. Two of the three RCTs (50, 51) reported no statistically significant differences in immune response biomarkers related to CV health between daily e-cigarette users and smokers, and the third (52) reported statistically significant lower white blood cells in daily e-cigarette users. Cross-sectional associations supported the mixed findings, reporting no statistical significance (53, 56–58, 77, 101), lower (53, 56, 102), and higher immune response (58) for daily e-cigarette users than for smokers.

#### Oxidative stress

Eight studies assessed differences in biomarkers of oxidative stress between daily e-cigarette users and non-smokers or smokers; three RCTs assessed CV health (50–52), one case-control (60) and one cross-sectional study (58) assessed respiratory health, and three cross-sectional studies were not linked to a specific physiological system (47, 56, 77). None of the studies examined exclusive e-cigarette users. The biomarkers of oxidative stress included salivary malondialdehyde, salivary mucins, and urinary 8-isoprostane. For a complete list of biomarkers, refer to Supplementary Table S2 and Outcome measures in Supplementary materials. The findings from all the RCTs suggested no significant differences in biomarkers of oxidative stress related to CV health between daily e-cigarette users and non-smokers (50, 51). Overall, the cross-sectional studies suggested mixed findings between daily e-cigarette users and non-smokers, with two (47, 77) finding no significant differences (one included markers linked to CV health) and two (58, 60) finding greater oxidative stress among daily e-cigarette users than among non-smokers. Comparing daily e-cigarette users to smokers, two of the three RCTs (50, 51) reported no significant difference, while the third showed statistically significant lower levels of biomarkers of oxidative stress for daily e-cigarette users (50). Cross-sectional associations comparing daily e-cigarette users to smokers suggested no significant differences in 40% (2/5) of the studies (58, 77), while 60% (3/5) showed significantly lower levels of biomarkers for e-cigarette users (47, 56, 60), one of which was related to CV health (47).

### Oral health

Twenty-three studies (60–65, 71, 74, 89, 90, 92, 93, 103, 111, 113–121) assessed periodontal (*n* = 20) and peri-implant (*n* = 6) oral health, among which 19 included daily e-cigarette use (60–65, 74, 89, 90, 92, 93, 103, 111, 113–118) (15 periodontal and 6 peri-implants) and five (61, 65, 103, 113, 115) included exclusive e-cigarette use (4 periodontal and one peri-implants).

#### Periodontal health

The periodontal health measures included clinical assessments [e.g., plaque index (PI), bleeding on probing (BOP), probing depth (PD), marginal bone loss (MBL), clinical attachment loss (CAL)], oral hygiene (number of missing teeth), self-reported measures of hard (teeth and bones) and soft (gums) tissues, and combined measures (self-reported dental health complaints and periodontal index).

Fifteen studies (60–65, 71, 74, 89, 90, 92, 93, 103, 111, 113–118) reported on periodontal health comparisons for daily e-cigarette use, four (61, 103, 113, 115) of which reported on exclusive e-cigarette use. From the 15 studies, two were cohort studies (115, 116), one of which assessed exclusive e-cigarette use. The cohort study (115) that compared exclusive e-cigarette users to non-smokers found no significant difference in clinical periodontal parameters at 3- and 6-month follow-ups (i.e., PI, BOP, PD, CAL, and number of missing teeth), while the other cohort showed significantly worse self-reported periodontal parameters (116) in daily e-cigarette users than in non-smokers. Cross-sectional studies on daily e-cigarette use reported no differences in 5/6 (83.3%) of the studies that assessed clinical parameters (61, 89, 93, 103, 118), two of which were on exclusive e-cigarette use (61, 103), while the sixth study showed poorer clinical outcomes in daily e-cigarette users than in non-smokers (114). Similarly, a case control (60) showed worse clinical periodontal outcomes (significantly higher PI, PD, CAL and MBL) for daily e-cigarette users than for non-smokers.

Two of the cross-sectional studies, however, showed poorer oral hygiene (number of missing teeth) for daily e-cigarette users than for non-smokers (103, 117), one of them was on exclusive e-cigarette use (103). One study comparing exclusive e-cigarette users to non-smokers showed a poorer self-reported clinical diagnosis of gum disease, bone loss, or periodontal disease (61). Finally, two studies (113, 118) reported significantly better outcomes for daily e-cigarette users (fewer missing teeth and marginal bone loss) than for non-smokers, and neither of these were on exclusive e-cigarette use.

The studies showed no significant difference in periodontal clinical outcomes but significantly better self-reported outcomes for daily e-cigarette users and exclusive e-cigarette users when compared to smokers. One cohort study showed no significant difference in periodontal clinical parameters between exclusive e-cigarette users and smokers (115). Three cross-sectional studies (60, 61, 90, 118), one of which looked at exclusive e-cigarette use (61) and a case-control supported the non-significant differences in clinical parameters, while two exclusive e-cigarette use studies reported better self-reported gingival health (61) and missing teeth (118) compared to smokers.

#### Peri-implant

Six studies (62–65, 90, 92) assessed differences in peri-implant parameters between daily e-cigarette users and non-smokers (62–65, 90, 92) and smokers (63, 65, 92), one of which assessed exclusive e-cigarette use (90). The outcomes included clinical periodontal assessments (i.e., PI, BOP, PD) around implants and radiographic parameters including peri-implant bone loss, clinical attachment loss, and radiographic bone loss (RBL). All the studies were cross-sectional, and most reported on more than one clinical outcome. For studies comparing daily e-cigarette users to non-smokers (62–65, 90, 92), most of the results were inconsistent within studies and led to “vote-splitting” (refer to Table 3). Four studies (62–64, 92) showed a mix of results, non-significant differences in PI (62–64, 92) and PD (62), poorer PD (63), BOP, PIBL (62) and MBL (92), and/or better clinical parameter BOP (63, 64, 92) for daily e-cigarette users than for non-smokers. Only one study showed significantly better clinical parameters (90), and only one study included exclusive users (65) showing poorer clinical parameters for the majority of clinical markers (PI, PD, and RBL) than non-smokers. Variable results for outcome measures were also seen within the studies comparing daily e-cigarette users with smokers, also leading to vote-splitting for these studies (63, 65, 92), including the one that assessed exclusive e-cigarette use (65). Clinical parameters of e-cigarette users either failed to differ from those of smokers (63, 90, 92), showed better (62, 63, 92), or worse clinical parameters (63).

### Respiratory health

Thirty-two studies (47, 50–52, 54, 59, 66–70, 73, 74, 86, 96, 112, 122–137) reported on respiratory symptoms and lung function, of which 15 (47, 50–52, 54, 66, 68, 73, 74, 96, 122, 126, 131–133) included daily e-cigarette use, two of which were on exclusive e-cigarette use (131, 132). From the 15 studies, 13 (47, 50–52, 54, 66, 68, 73, 96, 122, 131–133) reported on lung function or spirometry measures including forced expiratory volume in 1 s (FEV1), forced vital capacity (FVC), FEV1/FVC ratio, peak expiratory force (PEF), forced expiratory time (FET), and maximal forced expiratory flow at 25 and 75% of the pulmonary volume (MFEF25–75) and 10 (47, 50, 68, 73, 74, 96, 122, 126, 132, 133) reported on respiratory symptoms including self-reported coughing, wheezing, chest tightness, difficulties breathing, and phlegm/sputum production.

#### Lung function

Thirteen studies reported on lung function comparing daily e-cigarette users to non-smokers or smokers, of which nine [two RCTs (50, 51), one cohort (132), and six cross-sectional studies] (47, 54, 66, 96, 122, 131) compared the lung function of daily e-cigarette users to that of non-smokers, and only two of them were on exclusive e-cigarette use (131, 132). The two RCTs (50, 51) and half of the cross-sectional studies (47, 54, 96) comparing daily e-cigarette users to non-smokers and the cohort study (132) comparing exclusive e-cigarette users to non-smokers found no significant differences. Two cross-sectional studies (122, 131), one of which examined exclusive e-cigarette use (66), found statistically significant worse lung function in daily e-cigarette users than in non-smokers (FVC in one study (122) and in most of the measures [FVC, FEV1, FEV1/FVC ratio, and MFEF 25%–75% in the other (131)]). One additional cross-sectional study (66) reported mixed findings (no significant difference in mean alveolar ventilation, mean alveolar perfusion, oxygen saturation, peripheral oxygen saturation, and FVC in daily e-cigarette users but significantly better FEV1 and FEV1/FVC and worse perfusion heterogeneity and ventilation perfusion heterogeneity than in non-smokers). All the studies reporting poorer lung function between daily e-cigarette users and non-smokers were cross-sectional.

Ten studies compared the respiratory function of daily e-cigarette users to that of smokers, and none of them looked at exclusive e-cigarette use. Almost all of the studies reported no differences between these groups, including three out of the four RCTs (50, 51, 73), one cohort (133), and all the cross-sectional studies (47, 54, 96, 122). The other two studies (one RCT and one cohort) reported better lung function in daily e-cigarette users (52, 68) than in smokers.

#### Respiratory symptoms

Ten studies (two RCTs (52, 73), three cohorts (68, 132, 133), and five cross-sectional studies (47, 74, 96, 122, 126) compared respiratory symptoms between daily e-cigarette users and non-smokers or smokers, of which only one examined exclusive e-cigarette users (132). Among the cross-sectional studies comparing daily e-cigarette users to non-smokers (47, 74, 96, 122, 132), all but one reported no significant difference in respiratory symptoms. The one cohort study that examined exclusive e-cigarette use (132) also found no difference compared to non-smokers. One cross-sectional study (122) did report a significant difference, with daily e-cigarette users demonstrating increased respiratory symptoms when compared to non-smokers.

The results were mixed across the studies comparing daily e-cigarette users to smokers (47, 50, 68, 73, 96, 122, 133). None assessed exclusive e-cigarette use. Three of the four cohort and RCT studies found fewer respiratory symptoms (52, 68, 133); two cohorts (68, 133) found fewer exacerbations of chronic obstructive pulmonary disease (COPD) symptoms among COPD patients using e-cigarettes, one also reported lower (better) COPD assessment test scores (133), and an RCT found significantly less coughing (52) in e-cigarette users than in smokers. However, one RCT (73) reported no significant difference in combined respiratory symptoms in daily e-cigarette users compared to smokers. For the three cross-sectional studies, one showed better respiratory symptoms (47), and two reported non-significant differences (96, 122) between daily e-cigarette users and smokers. Respiratory symptoms were one of the few outcomes where e-cigarette users were compared to dual users. An RCT reported no difference (73) and a cross-sectional study (126) reported better lung function for e-cigarette users than for dual users. None compared exclusive e-cigarette users to smokers or dual users.

## Discussion

This systematic review provides a narrative synthesis of the most reported chronic health effects of e-cigarette use since the release of the NASEM review published in 2018 (19). When the NASEM report was drafted, human studies were not available for several plausible e-cigarette use-linked health outcomes (e.g., clinical CV outcomes, changes in respiratory function and respiratory diseases, and pregnancy outcomes), and conclusions were frequently based on evidence from studies using *in vitro* or animal models. Despite the recent surge in e-cigarette use-related studies (*n* = 180 studies involving 18 physiological health systems and presenting more than 40 outcomes), gaps in the evidence remain, and this review recognizes the need for stronger study designs and methods. It must also be recognized that the latency for the appearance of symptoms or signs of many health conditions is long; the recency of the introduction and use of e-devices may therefore preclude such findings during the timeframe of their use.

### Brief summary

To our knowledge, this is the first systematic review that stratifies outcomes by study design, exclusive e-cigarette use, and duration of e-cigarette exposure comparing e-cigarette users to non-smokers, traditional smokers, or dual-users, and assesses the certainty of evidence. The certainty of the evidence for all 11 outcomes was very low, owing to limitations in reporting and accounting for e-cigarette use exposure (exclusive, daily, and occasional), large cross-sectional designs, heterogeneity in findings, and validity of outcome measures. Only 51% of all the studies [62% of the 11 outcomes (*n* = 59)] reported on health effects associated with daily e-cigarette users. Moreover, only 20% of all the studies (21% of the 11 outcomes, *n* = 20), of which 11 were on daily users, reported on exclusive e-cigarette use. The lack of data on exclusive e-cigarette users and on the smoking history of daily e-cigarette users is a problem that makes it difficult to disentangle the long-term effects of e-cigarette use from the confounding effects of past cigarette smoking exposure. Establishing dose-response exposure gradients, both for e-cigarette users (i.e., frequency and duration for exclusive e-cigarette use) and in relation to comparison groups (i.e., compared to non-smokers and frequency and duration for former and current smokers and dual-users), would greatly improve the strength of the evidence by increasing confidence in comparison results. While new evidence has emerged, it is largely based on cross-sectional studies (81.7% of all the studies and 78% among the 11 selected outcomes) where causation is inferred rather than confirmed. Although the review identified some cohort and experimental studies, they were limited because of small samples and the potential for selection bias. There remains a need for ongoing, large, experimental and cohort studies among exclusive e-cigarette users that consider important individual factors such as health status, socioeconomic status, smoking duration, and history using valid and consistent health outcome measures.

In summary, among the experimental and cohort studies that compared exclusive e-cigarette users to non-smokers (*n*=6), no statistically significant differences was found for CVD (45, 82), respiratory inflammation (99), immune response (99), periodontal clinical parameters (115), peri-implant parameters (65), lung function (132), and respiratory symptoms (132). The relatively short duration of exposure to e-cigarette use may, at this point, limit the likelihood of identifying differences that may well emerge with longer exposure periods.

Comparing exclusive e-cigarette users to smokers, there were no statistically significant differences in biomarkers of oral inflammation (103), periodontal clinical parameters (115), and peri-implant parameters for BOP and PI, and there was better PD and bone loss (65). No studies compared exclusive e-cigarette users to non-smokers or smokers for either CV risk factors or oxidative stress, and none compared the lung function or respiratory symptoms of exclusive e-cigarette users to those of smokers. Moreover, no studies compared exclusive e-cigarette users to dual users.

### Comparisons to the literature

This review intended to update and fill the evidence gaps identified in the NASEM report (19) and more recent systematic reviews (32, 33, 35, 36) on chronic CV health effects of e-cigarette use. Previous reviews have found insufficient evidence on CV health outcomes, specifically changes in heart rate (32, 36), blood pressure (32, 35, 36) and cardiac geometry and function (19). Additionally, although based on few studies and limited duration of exposure, previous reviews have found no significant difference in the odds of CVD among e-cigarette users compared to non-smokers (33), but there were significant negative impacts on endothelial function, thrombogenesis, arterial stiffness, and long-term risk for coronary events (36). It is worth noting that the previous review evidence has been drawn from studies with varied exposure assessment methods such as including occasional e-cigarette users and not controlling for former cigarette use (33, 36). Despite the greater number of studies identified by the present review, the overall certainty of the evidence is very low and largely suggests no significant differences between e-cigarette users and non-smokers or smokers in terms of CVD, blood pressure, lipid profiles, and CV function. However, among the few examples where differences were observed, most were in the direction that would be hypothesized by exposure status, that is, e-cigarette users had poorer outcomes than non-smokers but better outcomes than smokers.

The NASEM report (19) also elaborated that although substantive evidence exists that e-cigarette components can induce oxidative stress, there was and remains limited evidence on long term exposure of humans resulting from e-cigarette use. The present review found no significant differences between daily e-cigarette users and non-smokers in biomarkers of oxidative stress related to CV health (50, 51), although the certainty of evidence from the RCT studies was very low. Additionally, no differences were identified in inflammatory biomarkers and immune response between exclusive e-cigarette users and non-smokers (99), but a reduction in oral inflammation in exclusive e-cigarette users compared to smokers (103) was observed. However, the certainty of evidence in all the cases was also very low.

Despite the growing number of studies that examine oral health, the findings from this review are mixed. Consistent results were observed for oral health inflammation, where 11 out of 15 cross-sectional studies reported significantly higher inflammation among daily e-cigarette users than among non-smokers. For most of the other oral health outcomes, however, more recent evidence cannot confirm the previous limited evidence of deterioration in periodontal health of e-cigarette users compared to that of non-smokers or the hypothesis that switching from cigarettes to e-cigarettes (19) reduces the risk of periodontal disease.

### Implications for research and practice

Despite the recent surge in e-cigarette use-related studies, gaps in the evidence remain, and this review calls for more research studies with stronger study designs and methods that focus on comparing exclusive e-cigarette users to non-smokers. The majority of studies in this review assessed health outcomes in healthy adults without consideration of within-population differences. As such, the findings of this review may not be generalizable to younger populations and specific subgroups (e.g., females vs. males, or ethnic groups). This is a key concern, as the number of youth who have never smoked previously but have used e-cigarettes is growing (3, 6, 17). More studies examining the health effects and risks in specific subpopulations (e.g., age group, sex, and ethnicity) are needed.

We found COI to be a concern; 20% (12/59) of the studies assessing the chronic health effects of daily e-cigarette use, included in this systematic review had a potential COI resulting from direct or indirect funding or affiliation with the pharmaceutical and tobacco industries Caution should be exercised when interpreting the evidence from these studies. Most studies with a COI reported either favorable or non-significant findings related to e-cigarette use and health outcomes.

We identified a substantial number of studies that reported on both injuries and poisonings (*n* = 19); asthma (*n* = 18, daily e-cigarette use = 2); COPD (*n* = 13, daily e-cigarette use = 3); genetic expressions (*n* = 12, daily e-cigarette use = 9); metabolic health (*n* = 9, daily e-cigarette use=3); self-reported health (*n* = 8, daily e-cigarette use = 2); pregnancy outcomes (*n* = 7, daily e-cigarette use = 0); cancer (*n* = 5, daily e-cigarette use = 1); sleep (*n* = 5, daily e-cigarette use = 0); cognitive health (*n* = 3, daily e-cigarette use = 1); reproductive health (*n* = 2, daily e-cigarette use = 1); sensory outcomes (*n* = 3, daily e-cigarette use = 2); neurological health (*n* = 1, daily e-cigarette use = 0); COVID-19 outcomes (*n* = 3, daily e-cigarette use = 1); voice quality (*n* = 1, daily e-cigarette use = 1), and multiple physiological systems (*n* = 1, daily e-cigarette use = 1). These studies were not narratively summarized in this review due to lower volume of evidence compared to the other health outcomes we reported on. The evidence is summarized, however, in appendices (available upon request) and recognizes the growing interest in studying the chronic health effects of e-cigarette use.

### Strengths and limitations

The strengths of this review include an *a priori* established study protocol with clear inclusion and exclusion criteria, development of search strategy by consultation with an information specialist, consideration of study design, specific/precise definition of e-cigarette use exposures (i.e., exclusive, daily, and occasional e-cigarette users) to further clarify the associations with various comparison groups (i.e., non-smokers, smokers, and dual users), and use of GRADE to assess the certainty of evidence associated with e-cigarette use.

The review included only *in vivo* studies on humans and did not take into consideration *in vitro*/mechanistic studies involving e-cigarettes.

Because of the heterogeneity of outcome assessment methods and effect statistics, the findings were summarized in a narrative manner based on the direction of association and statistical significance; the summary measures of effect and dose-response were not interpreted. Most of the studies did not report on e-cigarette or e-liquid brands or products used. This limited the analysis concerning the health effects of specific e-cigarette products and might have contributed to the mixed findings. Finally, much of the evidence was limited by the lack of report on e-cigarette use characteristics of duration and previous smoking exposure.

## Conclusions

This review provides an update on the human *in vivo* evidence on chronic health effects of e-cigarette use presented in the 2018 NASEM report. While there have been a large number of studies examining the chronic health effects of e-cigarette use, the certainty of the evidence remains very low largely because of cross-sectional study design, few studies with daily or exclusive e-cigarette use exposure, and potential COI. There remains a need for higher-quality interventional and prospective studies to assess causality, with a focus on exclusive e-cigarette users, to improve the certainty in longer-term health outcomes of e-cigarette users when compared to non-smokers, smokers, and dual-users. Additionally, future studies would benefit from exploring effects on different population groups (e.g., youth, young adults, ethnic groups) and effects of different e-cigarette compositions. The variability in the composition of e-cigarette liquids in different e-cigarette brands, with potential varying amounts of nicotine, heavy metals, flavorings, etc., may have contributed to the heterogeneity across the results, and warrants better reporting and investigation in future studies. The recency of the introduction and use of e-cigarette devices may also preclude the identification, at this point, of significant findings reflecting disordered function and enhanced risk of disease.

## Data availability statement

The original contributions presented in the study are included in the article/Supplementary material, further inquiries can be directed to the corresponding authors.

## Author contributions

RW, FB, MG, SP, SM, and WT conceptualized and designed the study. RW, FB, SP, MG, and WT performed data screening, extraction and analyses, and drafted the initial manuscript. AM, SS, AC, EW, AH, and JL assisted with the data screening and extraction. EW and AH assisted in data analysis and drafting the appendices tables. AP provided expertise for ECU and the physiological systems and assisted in interpreting the findings. All authors reviewed and revised the manuscript and approved the final manuscript as submitted and agree to be accountable for all aspects of the work.

## Conflict of interest

The authors declare that the research was conducted in the absence of any commercial or financial relationships that could be construed as a potential conflict of interest.

## Publisher's note

All claims expressed in this article are solely those of the authors and do not necessarily represent those of their affiliated organizations, or those of the publisher, the editors and the reviewers. Any product that may be evaluated in this article, or claim that may be made by its manufacturer, is not guaranteed or endorsed by the publisher.

## Author disclaimer

The content and views expressed in this article are those of the authors and do not necessarily reflect those of the government of Canada.

## Abbreviations

BOP: bleeding on probing
CAL: clinical attachment loss
CASP: Critical Appraisals Skills Programme
COI: conflict of interest
COPD: chronic obstructive pulmonary disease
CV: cardiovascular
CVD: cardiovascular disease
DUs: dual users
EVALI: e-cigarette- or vaping-associated lung illness
FEV1: first second of forced expiration
FVC: forced vital capacity
FET: forced expiratory time
GCF: gingival crevicular fluid
GRADE: grading of recommendation assessment, development, and evaluation
HDL: high-density lipoprotein
LDL: low-density lipoprotein
HNB: heat-not-burn
MBL: marginal bone loss
MFEF: maximal forced expiratory flow
MMP-2: matrix metalloproteinase-2
MMP-9: matrix metalloproteinase-9
NASEM: National Academies of Sciences, Engineering, and Medicine
PEF: peak expiratory force
PD: probing depth
PI: plaque index
PHAC: Public Health Agency of Canada
PRISMA: Preferred Reporting Items for Systematic Reviews and Meta-Analyses
RCT: randomized controlled trial
RBL: radiographic bone loss
RoB: risk of bias
THC: tetrahydrocannabinol
TSs: traditional smokers
USA: United States of America.
