# Supplementary material for: Chronic health effects associated with electronic cigarette use: A systematic review
Source: Front Public Health. 2022 Oct 6;10:959622. doi: 10.3389/fpubh.2022.959622 (PMC9584749; doi:10.3389/fpubh.2022.959622)
Supplement: Supplementary file 1 [file Table_1.pdf]

Supplemental S1: Search strategy

Table S1-1. Ovid Medline search strategy

#	Searches	Results
1	Electronic Nicotine Delivery Systems/ or vaping/	2703
2	("e-cigarette" or "e-cigarettes" or electronic cigarette or electronic cigarettes or electronic nicotine delivery or electronic nicotine device or electronic smoking device or "e-cig*").tw,kw,kf.	4551
3	((tobacco or nicotine or cannabi* or marijuana or marihuana) adj3 (vap* or heating device* or heating system*)).tw,kw,kf.	545
4	"heat not burn".tw,kw,kf.	95
5	(cigalike or ciga-like or ego-style or Joyetech or Ruyan or clearomizer or Vype or SKYCIG or MarkTen or Vuse or nicocigs or voke or JUUL or Aspire CF-VV* or kanger or "k-pin mini" or innokin endura or ohm go or eleaf or subvod or vaporfi or "smok" or resa stick*).tw,kw,kf.	168
6	(vaporizer* or vaporiser* or vape or vaping or vapes or vap* pens or vap* pen or dab pen? or wax pen? or dab vap* or wax vap* or e-pen or e-pens or atomizer* or atomiser*).tw,kw,kf.	2900
7	(e-liquid* or eliquid* or e-juice* or ejuice* or vape juice* or flavor juice* or flavour juice*).tw,kw,kf.	437
8	or/1-7	7193
9	(201709* or 201710* or 201711* or 201712* or 2018* or 2019*).dt,ez.	2642790
10	8 and 9	2454

Table S1-2. Ovid Embase search strategy

#	Searches	Results
1	tobacco heating system/ or vaping/ or electronic heating system/	1070
2	("e-cigarette" or "e-cigarettes" or electronic cigarette or electronic cigarettes or electronic nicotine delivery or electronic nicotine device or electronic smoking device or "e-cig*").tw,kw.	5450
3	((tobacco or nicotine or cannabi* or marijuana or marihuana) adj3 (vap* or heating device* or heating system*)).tw,kw.	643
4	"heat not burn".tw,kw.	128
5	(cigalike or ciga-like or ego-style or Joyetech or Ruyan or clearomizer or Vype or SKYCIG or MarkTen or Vuse or nicocigs or voke or JUUL or Aspire CF-VV* or kanger or "k-pin mini" or innokin endura or ohm go or eleaf or subvod or vaporfi or "smok" or resa stick*).tw,kw.	236
6	(vaporizer* or vaporiser* or vape or vaping or vapes or vap* pens or vap* pen or dab pen? or wax pen? or dab vap* or wax vap* or e-pen or e-pens or atomizer* or atomiser*).tw,kw.	3508
7	(e-liquid* or eliquid* or e-juice* or ejuice* or vape juice* or flavor juice* or flavour juice*).tw,kw.	547
8	or/1-7	8649
9	(201709* or 201710* or 201711* or 201712* or 2018* or 2019*).dc.	3748386
10	8 and 9	3382

Table S1-3. Ovid PsycINFO search strategy

#	Searches	Results
1	electronic cigarettes/	1036
2	("e-cigarette" or "e-cigarettes" or electronic cigarette or electronic cigarettes or electronic nicotine delivery or electronic nicotine device or electronic smoking device or "e-cig*").tw,id.	1594
3	((tobacco or nicotine or cannabi* or marijuana or marihuana) adj3 (vap* or heating device* or heating system*)).tw,id.	161
4	"heat not burn".tw,id.	15
5	(cigalike or ciga-like or ego-style or Joyetech or Ruyan or clearomizer or Vype or SKYCIG or MarkTen or Vuse or nicocigs or voke or JUUL or Aspire CF-VV* or kanger or "k-pin mini" or innokin endura or ohm go or eleaf or subvod or vaporfi or "smok" or resa stick*).tw,id.	74
6	(vaporizer* or vaporiser* or vape or vaping or vapes or vap* pens or vap* pen or dab pen? or wax pen? or dab vap* or wax vap* or e-pen or e-pens or atomizer* or atomiser*).tw,id.	366
7	(e-liquid* or eliquid* or e-juice* or ejuice* or vape juice* or flavor juice* or flavour juice*).tw,id.	92
8	or/1-7	1778
9	(201709* or 201710* or 201711* or 201712* or 2018* or 2019*).up.	334252
10	8 and 9	794
