# Supplementary material for: Chronic health effects associated with electronic cigarette use: A systematic review
Source: Front Public Health. 2022 Oct 6;10:959622. doi: 10.3389/fpubh.2022.959622 (PMC9584749; doi:10.3389/fpubh.2022.959622)
Supplement: Supplementary file 2 [file Table_2.pdf]

Supplement S2: Study characteristics

Color code and definitions of e-cigarette users (ECU) subgroups based on exposure:

ECU sub-group/s	Study final ECU exposure category	Exclusive ECU status	
		NO	YES
If study includes:<br>• "Daily users"<br>• "Daily AND occasional users" each in a separate group.	Daily e-cigarette use (ECU)	ECU could be former smokers or smoking status was unclear/ unspecified in the study	ECU were never smokers
If study includes:<br>• "Occasional users"<br>• "Daily OR occasional users" combined together in one group.	Occasional e-cigarette use (ECU)	ECU could be former smokers or it was unclear/ unspecified in the study	ECU were never smokers
If study includes:<br>• Exposure is not specified /unclear.<br>• Ever users.<br>• Combined daily or occasional users with an unclear exposure.	Unclear e-cigarette use (ECU)	ECU could be former smokers or it was unclear/ unspecified in the study	ECU were never smokers

Table S2.1: Cardiovascular health

First author (year)	Country of study, Study name	Population description	Outcome measures	Sample size analyzed			Age in years (range or mean (SD))	Exposure			Conflict of interest
				Total	Females (N, %)	Males (N, %)		Comparison groups[1]	ECU sub-groups	Exclusive ECU	
**Cardiovascular health (n =26)**											
**Randomized control trials** (n=6)											
(George et al., 2019) [2]	UK, VESUVIUS	Healthy adults	Endothelial function, arterial stiffness, flow-mediated dilation (FMD), systolic blood pressure, diastolic blood pressure, heart rate and pulse wave velocity	114	75, 66%	39, 34%	≥18	ECU, TS, DU	Occasional users	No	None
(Haziza et al., 2020) [3]	USA	Healthy adults smokers	Total cholesterol, HDL-c, LDL-c, Triglycerides, systolic blood pressure, diastolic blood pressure	160	64, 40%	96, 60%	37.7 (11.5)	ECU, TS NS	Daily users	No	Yes
(Ikonomidis et al., 2020)[4]	Greece	Smokers without cardiovascular disease	Systolic blood pressure, diastolic blood pressure, pulse wave velocity	40	32, 80%	8, 20%	-	ECU, TS	Daily users	No	None
(Ludicke et al., 2018)[5]	Switzerland	The sample consisted of healthy, smoking, Japanese participants	Total cholesterol, HDL-c, LDL-c, triglycerides, systolic blood pressure, diastolic blood pressure.	160	NR	NR	23-65	ECU, TS, NS	Daily users	No	Yes

[1] Comparison groups: Electronic cigarette users (ECU), traditional tobacco smokers (TS), dual users (DU), non-smokers (NS), (sometimes referred to as never users, or non-users, when this is the case a footnote will indicate that for each specific study).
[2] George et al. (2019): All check immunological health.
[3] Haziza et al. (2020): All check immunological health.
[4] Ikonomidis et al. (2020): Also check immunological health
[5] Ludicke et al. (2018): Also check immunological and respiratory health

(Ludicke et al., 2019)[6]	Switzerland	Healthy volunteers	HDL-c	815	339, 42%	476, 58%	30 years or older	ECU, TS, DU	Daily users	No	Yes
(Pulvers et al., 2020)[7]	USA	Healthy adults part of a multi-site study	Systolic blood pressure, diastolic blood pressure	186	75, 40%	111, 60%	43.3(12.5)	ECU, TS, DU	Daily users	No	Yes
**Pre-post studies** (n=1)											
(Ikonomidis et al., 2018)[8]	Greece	Participants from a hospital smoking cessation clinic	Augmentation index, diastolic blood pressure, heart rate, systolic blood pressure, peak wave velocity.	86	39, 56%	31, 44%	48(5)	ECU, TS, DU	Occasional users	No	None
**Cross-sectional studies (n=19)**											
(Aherrera et al., 2020)[9]	USA	Voluntary sample of ECU	Hypertension	150	59, 36%	91, 64%	30.1(9.6)	ECU, NS[10]	Daily users	No	None
(Arastoo et al., 2020)	USA	Healthy adults who were chronic ECU	Systolic blood pressure, diastolic blood pressure, mean blood pressure, heart rate	100	44, 44%	66, 66%	21-45	ECU, TS	Daily users	No	None
(Alzahrani et al., 2018)	USA, NHIS	Nationally representative sample of adult	Myocardial infraction	69 452	38 209, 55%	31 243, 44%	≥18	ECU, NS[11]	Daily and occasional users	No	None
(Badea et al., 2019)[12]	Romania	Random sample of healthy middle aged adults	Total cholesterol, HDL-c, LDL-c, Triglycerides.	150	115, 77%	35, 23%	-	ECU, TS, NS[13]	Daily users	No	None
(Farsalinos et al., 2019)	USA, NHIS	Adult respondents to a national survey	Myocardial infarction, coronary heart disease	59 779	32 595, 55%	26 984, 45%	≥ 18	ECU, NS[10]	Daily and occasional users	No	Yes
(Fetterman et al., 2020)	USA	Healthy adults	Brachial diameter, mean flow velocity,	466	206, 44%	260, 56%	21-45	ECU, NS[10] TS, DU	Daily users	No	None

[6] Ludicke et al. (2019): Also check respiratory health.
[7] Pulvers et al. (2020): Also check respiratory health.
[8] Ikonomidis et al. (2018): Also check immunological health.
[9] Aherrera et al. (2020): Also check immunological health.
[10] Aherrera et al. (2020): Authors referred to non-smokers group (NS) as non-users.
[11] Alzahrani et al. (2018): Authors referred to non-smokers group (NS) as non-users.
[12] Badea et al. (2019): Also check immunological health.
[13] Badea et al. (2019) : Authors referred to non-smokers group (NS) as non-users.

			flow-mediated dialation, shear stress, carotid-femoral pulse wave velocity, carotid-radial pulse wave velocity, augmented index, systolic blood pressure, diastolic blood pressure, heart rate, endothelial cell function, baseline mean flow velocity, hyperemic mean flow velocity								
(Haptonstall et al., 2020)	USA	Baseline characteristics of a sample of healthy young adults.	Systolic blood pressure, diastolic blood pressure, heart rate, mean blood pressure, peak shear rate, artery diameter, endothelial function, flow mediated dilation, velocity reactive hyperemia, shear stress reactive hyperemia	138	52, 38%	84, 62%	21-45	ECU, TS, NS[10]	Daily users	No	None
(Ip et al., 2020)	USA	Healthy young adults	Heart rate	145	63, 43%	82, 57%	21-45	ECU, TS NS[10]	Daily users	No	None
(T. Kim et al., 2020)	South Korea (Korean National Health and Nutrition Examination Survey (KNHANES) 2013-2015)	A nationally representative sample of men and women.	Hypertension<br>Low HDL-cholesterol<br>High triglycerides	14738	6716 (45.6%)	8022 (54.4%)	20 +	ECU, NS	Daily or Occasional users	No	None
(C. Kim et al., 2020)	South Korea, KNHNES	Nationally representative sample of male adult dual users.	Hypertension, hyperlipidaemia, cardiovascular disease	7 505	0, 0%	7 505, 100%	≥19	Dual-users, smokers, non-users	Occasional	None	

(Leavens et al., 2020)[14]	USA, MHS	Homeless adults respondents on a state-wide survey	Hypertension	4 148	1 858, 45%	2 265, 55%	≥18	ECU, TS, NS[10]	Occasional users	No	None
(Oliveri et al., 2020)[15]	USA,	Healthy adults	HDL-c	194	101, 48%	93, 52%	30-65	ECU, TS	Daily users	No	None
(Osei et al., 2019)	USA, BRFSS	Nationally representative sample of adults	Cardiovascular disease, premature cardiovascular disease	449 092	245 678, 55%	203 414, 45%	-	ECU, NS[10]	Daily and occasional users	Yes	None
(Parekh et al., 2020)	USA, BRFSS	Nationally representative sample of young adults	Stroke	161 529	85 772, 53%	75 757, 47%	18-44	ECU, TS, NS[10]	Occasional users	Yes	None
(Podzolkov et al., 2020)	Russia	Adolescent volunteers from a medical university	Blood pressure, albuminuria level, augmentation index arterial elasticity	270	174, 64%	96, 36%	21.2 (2.3)	ECU, TS, NS[10]	Daily users	No	None
(Rodu & Plurphanswat, 2020)	USA, PATH	Baseline for a nationally representative cohort of adolescents	Heart attack	25 137	-	-	-	ECU, NS[10]	Daily and occasional users	No	Yes
(Sakaguchi et al., 2021)[16]	Japan,	Healthy adults who took part in a three-group, multicentre study recruited by 3H Medi Solution Inc,.	Total cholesterol, HDL-c, LDL-c, Triglycerides	459	116, 25%	343, 75%	Overall: 45.4 (9.3) NS: 44.6 (8.7), ECU: 45.4 (9.4), TS: 45.9 (9.7)	ECU, TS NS[10]	Daily users	No	Yes
(Vindhyal et al., 2020)	USA, NHIS	Nationally representative sample of adults	Myocardial infarction, stroke, coronary heart disease	16 877	8 020, 48%	8 835, 52%	40.8	ECU, NS[10]	Daily users	Yes	None

[14] Leavens et al. (2020): Also check respiratory health.
[15] Oliveri et al. (2020): Also check immunological health.
[16] Sakaguchi, et al. (2021): Also check immunological and respiratory health.

(Wang et al., 2018)[17]	International, Health sHeart Study	Internet-based, longitudinal cohort	High blood pressure, cardiac arrest (heart attack), congestive heart failure, coronary artery disease, stroke	39 747	12 047, 30 %	27 600, 70%	18 years and older	ECU, NS	Occasional users	No	None

Table S2.2: Immunological health

First author (year)	Country of study, Study name	Population description	Outcome measures	Sample size analyzed			Age in years (range or mean (SD))	Exposure			Conflict of interest
				**Total**	**Females** (N, %)	**Males** (N, %)		**Comparison groups**	**ECU sub-groups**	**Exclusive ECU**	
**Immunological Health (n=45)**											
**Randomized controlled trials (n=7)**											
(George et al., 2019)[18]	UK, VESUVIUS	Healthy adults	Inflammatory biomarkers: C-reactive protein<br><br>Immune response indicators: Platelet activation inhibitor-1 (PAI-1)<br>Tissue plasminogen activator	114	75, 66%	39, 34%	≥18	ECU, TS, DU	Occasional users	No	None
(Haziza et al., 2020)[19]	USA	Healthy adults smokers	Inflammatory biomarker:<br>HS-C Reactive Protein, sICAM-1, Fibrinogen<br><br>Immune response indicators:<br>Total white blood cell counts, platelet activity—11-DTX-B2	160	64, 40%	96, 60%	37.7(11.5)	ECU, TS, NS	Daily users	No	Yes

[17] Wang et al. (2020): Also check respiratory health.
[18] George et al. (2019): All immunological health indicators were measured as indicator to cardiovascular health. Also check cardiovascular health.
[19] Haziza et al. (2020): All immunological health outcomes were measured in context of cardiovascular health. Also check cardiovascular health.

First author (year)	Country of study, Study name	Population description	Outcome measures	Sample size analyzed			Age in years (range or mean (SD))	Exposure			Conflict of interest
				Total	Females (N, %)	Males (N, %)		Comparison groups	ECU sub-groups	Exclusive ECU	
			Oxidative stress- 8-epi-prostaglandin F2, alpha (8-epi-PGF2a)								
(Ikonomidis et al., 2020)[20]	Greece	Smokers without cardiovascular disease	Biomarker of oxidative stress:<br><br>Malondialdehyde levels.	40	32, 80%	8, 20%	-	ECU, TS	Daily users	No	None
(Ludicke et al., 2018)[21]	Switzerland	The sample consisted of healthy, smoking, Japanese participants	Biomarker of oxidative stress—8-epi-PGF2α;<br><br>Immune response: platelet activity—11-DTX-B2, total white blood count<br><br>Inflammatory biomarker:<br><br>HS-C reactive proteins, ICAM-1	160	NR	NR	23-65	ECU, TS, NS[22]	Daily users	No	Yes
(Ludicke et al., 2019)[23]	Switzerland	The sample consisted of healthy adult volunteers who were not motivated to	Immune response: Total white blood cell count<br><br>Oxidative stress:	815	476, 59%	339, 41%	30+<br><br>ECU 44.2 (9.64), DU	ECU, TS, DU	Regular users	No	Yes

[20] Ikonomidis et al. (2020): Also check cardiovascular health

[21] Ludicke et al. (2018): All immunological health outcomes were measured in context of cardiovascular health. Also check respiratory, and cardiovascular health.

[22] Ludicke et al. (2018): NS are abstinent participants.

[23] Ludicke et al. (2019): All immunological health outcomes were measured in context of cardiovascular health. Also check in respiratory and cardiovascular health.

First author (year)	Country of study, Study name	Population description	Outcome measures	Sample size analyzed			Age in years (range or mean (SD))	Exposure			Conflict of interest
				Total	Females (N, %)	Males (N, %)		Comparison groups	ECU sub-groups	Exclusive ECU	
		quit smoking within the next 6 months.	8-epi-prostaglandin F2 alpha (8-epi-PGF2a), platelet activation: 11-DTX-B2<br><br>Inflammatory biomarker: sICAM-1				43.8 (9.77), TS 45.2 (9.55)				
(Song, Reisinger, et al., 2020)	USA	This study consisted of healthy adults who volunteered for bronchoscopy	Immune response: Macrophages, lymphocytes, neutrophils, eosinophils,<br><br>Inflammatory biomarkers in bronchoalveolar lavage fluids: IFN-γ, IL-1β, IL-2, IL-4, IL-6, IL-8, IL10, IL-12, IL-13, TNF-α	29	NR	NR	21-30	ECU, NS	Daily users	Yes	None
(Rebuli et al., 2021)	USA	The study consisted of healthy volunteers	Nasal lavage fluid IgA<br>Inflammatory biomarkers in nasal epithelial-lining fluid MCP-1, MIP1β, MIP-1α, IL-6, IL-12, IFN- γ, IL-1α, IL-2, VEGF.	49	22, 45%	27, 55%	18-40	ECU, NS	Daily users	No	None
**Pre-post studies (n=1)**											

First author (year)	Country of study, Study name	Population description	Outcome measures	Sample size analyzed			Age in years (range or mean (SD))	Exposure			Conflict of interest
				Total	Females (N, %)	Males (N, %)		Comparison groups	ECU sub-groups	Exclusive ECU	
(Ikonomidis et al., 2018)[24]	Greece	Participants from a hospital smoking cessation clinic	Immune response indicators:<br><br>Biomarkers of platelet activation PFA-100<br><br>Biomarker of oxidative stress: Malondialdehyde	40	32, 80%	8, 20%	ECU: 46.8 (10.9)<br><br>TS 43.2 (11.7)	ECU, TS	Occasional users	No	None
**Case-control studies** (n=2)											
(Ibraheem et al., 2020)[25]	Saudi Arabia	Healthy male adults recruited from the outpatient department of the College of Dentistry, Jazan University.	GCF Volume	120	0	120, 100%	Overall: 45.4<br><br>NS: 43.8 (1.7)<br><br>TS: 46.5 (5.3)<br><br>ECU: 45.6 (3.6)	NS, TS, ECU	Daily users	No	None
(Karaaslan et al., 2020)[26]	Turkey	Participants recruited from the Department of Periodontology of Usak University.	GCF Volume<br><br>Biomarker of oxidative stress<br><br>GsH-Px8, hydroxydeoxyguan-osine<br><br>Inflammatory biomarkers: IL-8,	57<br><br>19 smokers, 19 e-cig users and 19 former smokers	18 (31.6%)	39 (68.4%)	29-39	ECU, TS	Daily users (at least 12 months)	No	None

[24] Ikonomidis et al. (2020): Also check cardiovascular health.
[25] Ibraheem et al. (2020): Also check oral health.
[26] Karaaslan, et al. (2020): Also check oral health.

First author (year)	Country of study, Study name	Population description	Outcome measures	Sample size analyzed			Age in years (range or mean (SD))	Exposure			Conflict of interest
				**Total**	**Females** (N, %)	**Males** (N, %)		**Comparison groups**	**ECU sub-groups**	**Exclusive ECU**	
			TNF-α								
**Cross-sectional studies (n=35)**											
(Al Deeb et al., 2020)[27]	Saudi Arabia	The sample consisted of 71 Individuals with peri - implant mucositis (p-iM)	Inflammatory biomarkers:<br><br>MMP-8<br>TNF-α (pg/mL)	71	0	71, 100%	30.2(4.4)	ECU, NS, TS	Daily users (more than one month, and every day use)	No	None
(Al-Aali et al., 2018)	Saudi Arabia	The sample consisted of participants from a single clinic	Inflammatory biomarkers:<br><br>Peri-implant sulcular fluid (PISF) Volume<br>Pro-inflammatory cytokines: TNF-α<br>IL-1β	92	0	92, 100%	NS 42.6 (2.7)<br><br>ECU: 35.8 (6.2)	ECU, NS	Daily users (at least one year)	No	None
(Al-Hamoudi et al., 2020)	USA	The sample consisted of individuals with moderate chronic periodontitis (CP), who were recruited to investigate the effect of scaling and root planning	Inflammatory biomarkers at baseline.<br><br>GCF volume (μL)<br>IL-4<br>IL-10<br>IL-11<br>IL-13 (pg/μL)	71	9, 12.7%	62, 87.3%	ECU: 47.7 (5.8),<br><br>NS: 46.5 (3.4)	ECU, NS[28]	Daily users (more than one month)	No	None

[27] Al Deeb et al. (2020): All immunological health outcomes were collected from peri-implant sulcular fluid (PISF) for periodontal outcomes. Also check oral health.
[28] Al-Hamoudi et al. (2020), NS are never smokers.

First author (year)	Country of study, Study name	Population description	Outcome measures	Sample size analyzed			Age in years (range or mean (SD))	Exposure			Conflict of interest
				Total	Females (N, %)	Males (N, %)		Comparison groups	ECU sub-groups	Exclusive ECU	
		(SRP) on the expression of anti-inflammatory cytokines.									
(AlQahtani et al., 2018)[29]	Saudi Arabia	Adults with dental implants	Inflammatory biomarkers:<br><br>PISF Volume TNF-α, IL-6, IL-1β	160	0	160, 100%	ECU: 34-53<br><br>TS: 34<br><br>NS: 41.8	ECU (ENDS users), TS, NS with periodonti-tis and NS without periodonti-tis	Daily users (at least once daily for the past 12 months)	Yes	None
(F. Alqahtani et al., 2019)	Saudi Arabia	Adults with dental implants	Inflammatory biomarkers:<br><br>PISF Volume	102	0	102, 100%	ECU: 33.5 (0.7)<br><br>NS: 32.2(0.6)<br><br>TS: 36.3 (1.2)	ECU, NS, TS.	Daily users (at least once daily for at least 1 year)	No	None
(S. Alqahtani et al., 2020)[30]	USA	The sample consisted of healthy volunteers recruited from Purdue University campus and local shops selling ENDS products.	Inflammatory biomarkers: IL-1β, IL-6, IL-8, TNF-α<br><br>inflammatory mediators: Prostaglandin E2, 16-phenoxy tetranor, 17-phenyl trinor-13, and 14-	30	12, 40%	16, 60%	NS: 23.2 (3.62)<br><br>ECU: 20.4(1.82)	NS, ECU	Daily users and some occasio nal tobacco smokin g (Unclea r)	No	None

[29] AlQahtani et al. (2018): Immunological indicators were measured as indicators of oral health. Also check oral health.
[30] AlQahtani et al. (2020): Immunological indicators were measured as indicators of oral health.

First author (year)	Country of study, Study name	Population description	Outcome measures	Sample size analyzed			Age in years (range or mean (SD))	Exposure			Conflict of interest
				Total	Females (N, %)	Males (N, %)		Comparison groups	ECU sub-groups	Exclusive ECU	
			dihydro prostaglandin, D4 methyl ester, leukotriene E4, pentosidine.<br><br>Factors inhibiting inflammation: Ganglioside; Glutathionyl spermine; angiotensin II, phosphotrosyl-angiotensin II, reduced coenzyme F420								
(ArRejaie, 2019)[31]	Saudi Arabia	Individuals were enrolled from the department of prosthetic dental sciences, King Saud University	PISF Volume<br><br>inflammatory biomarkers: IL-1β, MMP-9			95, 100%		NS, TS, ECU	Daily users (for at least 1 year)	No	None
(Ashford et al., 2020)[32]	USA	The sample consisted of a convenience sample collected from English speaking undergraduate college students on	Inflammatory biomarkers:<br><br>IL-2, IL-4, IL-6, IL-8, IL-10, IL-12, IL-13, TNF-α, INF-γ,	61	29, 47%	32, 53%	18-25	NS, ECU	Occasi onal users	No	None

[31] ArRejaie et al. (2019): Immunological indicators were measured as indicators of peri-implants clinical assessments. Also check oral health.
[32] Ashford et al. (2020): Also check respiratory health.

First author (year)	Country of study, Study name	Population description	Outcome measures	Sample size analyzed			Age in years (range or mean (SD))	Exposure			Conflict of interest
				Total	Females (N, %)	Males (N, %)		Comparison groups	ECU sub-groups	Exclusive ECU	
		campus at the University of Kentucky.									
(Badea et al., 2019)[33]	Romania	Random sample of healthy middle aged adults	Indicators of oxidative stress: Vitamin A, Vitamin E, albumin, antioxidants.<br><br>Immune response indicators: White blood cell count, neutrophil counts, lymphocyte counts, monocyte counts, eosinophil counts, basophils counts	150	115, 77%	35, 23%	-	NS, TS, ECU, DU	Daily users	No	None
(BinShabaib et al., 2019)[34]	Saudi Arabia	Volunteer individuals	Inflammatory biomarkers:<br><br>GCF Volume, IL-1β, IL-6, IFN-γ,TNF-α, MMP-8	135	11, 8%	124, 92%	NS: 40.6 (3.3)<br><br>TS: 44.2 (3.5)<br><br>ECU: 36.5 (1.7)	NS, TS, ECU	Daily users (for at least once daily)	No	None
(Cichonska et al., 2019)[35]	Potland	The sample was collected from a healthy sample of students of the Medical	Inflammatory biomarkers: Immunoglobulin A (IgA). Antimicrobial	125	NR	NR	20-30	NS, TS, ECU	Daily users	No	None

[33] Badea et al. (2019): Also check cardiovascular health.
[34] BinShabaib et al. (2019): Immunological indicators were measured as indicators of oral health. Also check oral health.
[35] Cichonska et al. (2019): Immunological indicators were measured from saliva samples for oral antibacterial health outcomes.

**First author (year)**	**Country of study, Study name**	**Population description**	**Outcome measures**	**Sample size analyzed**			**Age in years** (range or mean (SD))	**Exposure**			**Conflict of interest**
				**Total**	**Females** (N, %)	**Males** (N, %)		**Comparison groups**	**ECU sub-groups**	**Exclusiv e ECU**	
		University of Gdansk and young patients	enzymes (immune response), lactoferrin lysozyme.								
(Faridoun et al., 2021)	USA	The sample was collected from adult individuals at the University of Maryland School of Denistry	Inflammatory biomarkers: IL-6, IL-8, IL-1β, TNF-α, IL-10, IL-1RA, c-reactive protein.	64	27, 42.2%	37, 57.8%	28-83<br><br>51.66 (16.81)	NS, TS, ECU, DU	Not specifie d	No	NR
(Ganesan et al., 2020)[36]	USA	Periodontal healthy individuals with attachment loss ≤ 1; less than three sites with 4 mm of probe depths (PD); bleeding index (BOP) ≤ 20%	Inflammatory biomarkers: IL-2,IL-6, GM-CSF, INF-γ, TNF-α, IL-10	123	72, 58.5%	51, 41.5%	21-35	NS, TS, DU	Daily users (daily for at least 3 months)	No	None
(Gavrilin et al., 2020)	USA	Three separate samples were taken, one healthy cohort, one HIV+ smoking cohort, and one pneumonia patient cohort. These	Inflammatory biomarkers:<br><br>PYCARD/ASC in lung bronchoalveolar lavage fluid (BAL)	157	121, 77%	36, 23%	Healthy cohort: NS: 26 (21–30) ECU: 27 (21–30) TS: 26 (21–30) HIV cohort: 42.9 (1.1)	NS<br><br>TS<br><br>ECU<br><br>HIV+ TS	Daily users	No	None

[36] Ganesan et al. (2021): Immunological indicators were measured as indicators of oral health. Also check oral health.

First author (year)	Country of study, Study name	Population description	Outcome measures	**Sample size analyzed**			Age in years (range or mean (SD))	**Exposure**			Conflict of interest
				**Total**	**Females** (N, %)	**Males** (N, %)		**Comparison groups**	**ECU sub-groups**	**Exclusive ECU**	
		individuals were collected in three separate studies.					Pneumonia cohort: 55.2 (2.4)				
(Ghosh et al., 2019)	USA	Recruitment of healthy population	Protein Outcomes (BAL Protease levels)<br><br>Protease, MMP-2, MMP-9, Neutrophil elastase<br><br>Protease inhibitors A1AT,SLPI, TIMP-1, TIMP-2,	42	22, 52%	20, 48%	NS 25.79 (7.29)<br><br>TS: 29.50 (5.59)<br><br>ECU: 26.07 (8.30)	NS, TS, ECU	Daily users	No	None
(Ikonomidis et al., 2018)[37]	Greece	Participants from a hospital smoking cessation clinic	Biomarker of oxidative stress : Malondialdehyde	70	39, 56%	31, 44%	48 (5)	ECU, TS	Occasional users	No	None
(Jackson et al., 2020)	USA	The sample was collected from healthy adult individuals recruited by newspaper and magazine advertisements in Rochester NY from 2016-2019	Immune response indicators:<br><br>Immunoglobulin (IgE / IgG)<br><br>- collected from plasma samples to measure immune outcomes	48	NS 57.69%<br><br>ECU 54.54%	NS 42.30%<br><br>ECU 45.45%	NS 33.88 (14.07)<br><br>ECU 35.54 (12.21)	NS, ECU	Daily users	Yes	None

[37] Ikonomidis et al. (2018): Also check cardiovascular health.

First author (year)	Country of study, Study name	Population description	Outcome measures	Sample size analyzed			Age in years (range or mean (SD))	Exposure			Conflict of interest
				Total	Females (N, %)	Males (N, %)		Comparison groups	ECU sub-groups	Exclusive ECU	
(Kelesidis et al., 2020)	USA	The sample consisted of healthy adults	Immune response indicators:<br><br>Neutrophils,<br>Monocytes,<br>T-cells,<br>NK Cells,<br>B-cells	33	14, 42%	19, 58%	21-45<br><br>NS<br>24.3 (2.15)<br><br>TS:<br>24.9 (4.08)<br><br>ECU:<br>24.1 (4.34)	NS, TS, ECU	Regular users	No	None
(Lee et al., 2020)	USA	Dataset 1: 30 healthy subjects (21–30 years old) with no prior history of e-cig or tobacco<br><br>Dataset 2: Patients were aged 18–55 years old and were all former cigarette smokers<br><br>Dataset 3: Comparator groups - 49 lung squamous cell carcinoma (LUSC) patients from The Cancer Genome Atlas	Immune response indicators:<br>Immune cell type Infiltration (naïve B-cells, memory B-cells, plasma cells, CD8 T-cells, CD4 naïve T-cells, CD4 memory resting T-cells, CD4 memory activated T-cells, follicular helper T-cells, regulatory T-cells, gamma-delta T-cells, resting NK cells, activated NK cells, monocytes, M0-M2macrophages, resting dendritic cells, activated dendritic cells, resting mast cells, activated mast cells, eosinophils, neutrophils.)	115	NR	NR	Dataset 1<br>21-30<br><br>Dataset 2<br>18-55<br><br>Dataset 3<br>NR	NS, ECU, ECU (former TS)	Regular users	Yes	None

First author (year)	Country of study, Study name	Population description	Outcome measures	Sample size analyzed			Age in years (range or mean (SD))	Exposure			Conflict of interest
				Total	Females (N, %)	Males (N, %)		Comparison groups	ECU sub-groups	Exclusive ECU	
		(TCGA). Subjects were matched for age, gender, and race.									
(Mainous et al., 2020)[38]	USA, NHANES 2015-2016	Adult participants, with no pre-existing heart conditions, who responded to the National Health and Nutrition Examination survey, a nationally representative survey.	Inflammatory biomarkers:<br><br>C-Reactive Protein	4659	NR	NR	20+	NS, ECU, TS, DU	Not specified	No	None
(Menicagli et al., 2020)[39]	Italy	Healthy male adults	Biomarkers of oxidative stress:<br><br>Salivary malondialdehyde (MDA)<br><br>Salivary mucins (SM)	51	0	51, 100%	32-41	NS, ECU	Not Specified	No	None
(Mokeem et al., 2018)[40]	Saudi Arabia	No details on recruitment method. 39 cigarette-	Inflammatory biomarkers: Whole Salivary Flow Rate	154	0	154, 100%	NS 40.6 (4.5)<br><br>TS	NS, TS, ECU	Daily users (at least 12	Yes	None

[38] Mainous et al. (2020): C-reactive protein was collected as inflammatory biomarker of cardiovascular health.
[39] Serra et al. (2020): Oxidative stress is measured in the context of oral health.
[40] Mokeem et al. (2018): All immunological indicators measured as indicators of oral health. Also check oral health.

First author (year)	Country of study, Study name	Population description	Outcome measures	Sample size analyzed			Age in years (range or mean (SD))	Exposure			Conflict of interest
				Total	Females (N, %)	Males (N, %)		Comparison groups	ECU sub-groups	Exclusive ECU	
		smokers, 40 waterpipe smokers, 37 E-cig users and 38 never-smokers.	IL-1β<br>IL-6				42.4 (5.6);<br><br>ECU<br>28.3 (3.5)		months, and never smoked tobacco in the past		
(Moon et al., 2020)	South Korea, KNHANES	Healthy adult men who participated in the first year of the 7th KNHANES Survey	Inflammatory biomarkers:<br>High sensitivity C-reactive protein,<br>Uric acid	1208	0	1208, 100%	19-65<br><br>NS<br>38.42 (13.25)<br><br>TS<br>42.25 (11.27)<br><br>ECU:<br>37.08 (11.54)	NS, TS, ECU	Occasional users	No	None
(Oliveri et al., 2020)[41]	USA	Individuals living in the Northeast, Midwest, South, and West US Census Bureau Regions and lived within 30miles of the LabCorp Patient Service Center	Biomarkers of immune response:<br><br>White blood cells<br><br>Biomarker of platelet activation:<br>11-dehydrothromboxane B2<br><br>Biomarker of oxidative stress:<br>8-epi-prostaglandin<br><br>Inflammation biomarker:	194	93, 48%	101, 52%	30-65	TS, ECU	Daily users	No	Yes

[41] Oliveri et al. (2020): Also check cardiovascular health.

First author (year)	Country of study, Study name	Population description	Outcome measures	Sample size analyzed			Age in years (range or mean (SD))	Exposure			Conflict of interest
				Total	Females (N, %)	Males (N, %)		Comparison groups	ECU sub-groups	Exclusive ECU	
			sICAM-1								
(Perez et al., 2020)[42]	USA	Participants were divided into 3 groups based on exposure status.<br><br>TS =23 mean age 39.5, ECU =22 mean age 24.0 and healthy controls =20 mean age 28.0	Biomarkers of inflammation :<br><br>TNF-α, IL-6, IL-33, IL-8, IL-10, IL-13, YKL-40,<br><br>Biomarkers of immune response: White blood cells, neutrophils, lymphocytes, eosinophils, monocytes, or basophils	65	41, 63%	24, 37%	18-55<br><br>NS:<br>28.0 (25.5-34.0)<br><br>ECU<br>24.0 (20.3-28.0)<br><br>TS<br>39.5 (30.5-48.0)	NS, ECU, TS	Daily users	No	None
(Pushalkar et al., 2020)[43]	USA	Adults diagnosed with mild, moderate, or severe periodontal disease, but otherwise healthy	Biomarkers of inflammation :<br><br>INF-γ, IL-10, IL-12, IL-13, IL-1β, IL-2, IL-4, IL-6, IL-8, TNF-α<br><br>-	119	NS<br>43.6%<br><br>TS<br>20%<br><br>ECU<br>22.5%	NS:<br>56.4%<br><br>TS:<br>80%<br><br>ECU:<br>77.5%	NS[44]<br>M:28.8 (6.1)<br>F: 38.4 (13.8)<br><br>TS:<br>M: 46.4 (10.0)<br>F:44.6 (12.0)<br><br>ECU<br>M: 36.0 (9.5)	NS, TS, ECU	Daily users	No	None

[42] Perez et al. (2020): All immunological indicators were measured via sputum and serum as a biomarker of pulmonary health. Also check respiratory health.
[43] Pushalkar et al. (2020): Immunological indicators were measured as indicators of oral health.
[44] Pushalkar et al. (2020): NS are never smokers.

First author (year)	Country of study, Study name	Population description	Outcome measures	Sample size analyzed			Age in years (range or mean (SD))	Exposure			Conflict of interest
				Total	Females (N, %)	Males (N, %)		Comparison groups	ECU sub-groups	Exclusive ECU	
							F: 35.7 (16.6)				
(Reidel et al., 2018)[45]	USA	Healthy adults aged 18-55	Biomarkers of oxidative stress:<br>Myeloperoxidase (MPO)<br>Aldehyde dehydrogenase 3A1<br>Nucleobindin 1<br>Thioredoxin<br>Glutathion S-transferase<br>Betamicroseminoprotein<br><br>Immune response:<br>Neutrophil count<br>Neutrophil elastase<br>Proteinase 3<br>Lysozyme C<br>Azurocidin<br>Coronin 1<br>Protein arginine deminase 4<br>Mucins (MUC5B MUC5AC)<br>Deleted in malignant brain tumours 1 (DMBT1)<br>Lactotransferrin<br>Trefoil factor 3<br><br>Inflammatory biomarkers:	30	14, 47%	16, 53%	18-55<br><br>NS 29.45 (2.36)<br><br>TS 33.00 (1.7)<br><br>ECU: 21.83 (0.82)	NS, TS, ECU	Daily users	No	None

[45] Reidel et al. (2018): All indicators were collected via sputum and measured as an indicator of respiratory/immunological health.

First author (year)	Country of study, Study name	Population description	Outcome measures	Sample size analyzed			Age in years (range or mean (SD))	Exposure			Conflict of interest
				Total	Females (N, %)	Males (N, %)		Comparison groups	ECU sub-groups	Exclusive ECU	
			-Anti-inflammatory lipid mediator: MMP9<br>- s100A8/ s100A9								
(Sakaguchi et al., 2021)[46]	Japan	Healthy adults who took part in a three-group, multicentre study recruited by 3H Medi Solution Inc,.	Immune response indicator:<br>White blood cell count<br><br>Indicator of platelet activation<br>11-dehydro thromboxane B2,<br>2,3-dinor thromboxane<br><br>oxidative stress indicators:<br>8-epi-prostaglandin<br><br>Inflammation biomarker:<br>sICAM-1	459	343, 75%	116, 25%	Overall: 45.4 (9.3)<br><br>NS<br>44.6 (8.7)<br><br>ECU 45.4 (9.4)<br><br>TS<br>45.9 (9.7)	NS, TS, ECU	Daily users	No	None
(Sakamaki-Ching et al., 2020)	USA	Subjects were recruited through media and flyers in Buffalo New York to provide urine samples.	Biomarkers of oxidative stress<br><br>8-isoprostane,<br>8-hydroxydeoxyguaosine<br>Metallothinonein	53	27, 51%	26, 49%	19-75<br><br>44.8 (14.0)	NS, TS, ECU	Not specified	No	Yes
(Shields et al., 2020)	USA	Convenience sample of healthy adults	Immune response: Presence of lipid-laden macrophages	64	40, 62.5%	24, 37.5%	21-45<br><br>25.5 (3.4)	NS, TS, ECU	Daily users	No	None

[46] Sakaguchi et al. (2021): Also check respiratory and cardiovascular health.

First author (year)	Country of study, Study name	Population description	Outcome measures	Sample size analyzed			Age in years (range or mean (SD))	Exposure			Conflict of interest
				Total	Females (N, %)	Males (N, %)		Comparison groups	ECU sub-groups	Exclusive ECU	
		recruited through Ohio State University study, through website, print media, TV, radio, and Craigslist.	(LLM) in bronchoalveolar lavage fluids								
(Singh et al., 2019)[47]	USA	Adults recruited through the General Clinical Research Center of the University of Rochester Medical Center through local newspapers and magazines advertisement, word of mouth, and flyers posted in and around the university campus.	Inflammatory biomarkers: IL-1α, IL-1β,IL-6, IL-8, IL-10, IL-13, IL-33, TNF-α, INF-γ, GM-CSF<br><br>Anti-inflammatory lipid mediators MMP-9,EN-RAGE, RAGE, S100A8, S100A9, Galectin-3, Uterglobin/CC-10, CC16<br><br>Inflammatory mediators: CXCL1 MCP-1,MIP-1α, MIP-1β,RANTES, Eotaxin,CXCL2,G-CSF,Leukotriene E4 Resolvin D1, Resolvin D2<br><br>Other inflammation indicator	48	21, 44%	27, 56%	21-65<br><br>TS: 33.9 (14.1)<br><br>ECU: 35.5 (12.2)	NS, ECU	Not specified	No	None

[47] Singh et al. (2019): Also check respiratory health

First author (year)	Country of study, Study name	Population description	Outcome measures	Sample size analyzed			Age in years (range or mean (SD))	Exposure			Conflict of interest
				Total	Females (N, %)	Males (N, %)		Comparison groups	ECU sub-groups	Exclusive ECU	
			ICAM-1<br><br>Immune response indicators:<br>Growth factors:<br>endothelial growth factor (EGF), vascular endothelial growth factor (VEGF), β-nerve growth factor (NGF), platelet-derived growth factor-AA (PDGF), stem cell factor (SCF), hepatocyte growth factor (HGF) and placental growth factor (PGF),<br>BDNF<br>BMP-2, TGC-α, FGF/GFG2<br><br>Extracellular matrix breakdown indicators:<br>Desmosine<br><br>Immune response/tissue injury:<br>PAI-1/Serpine-1<br>Biomarkers of oxidative stress:<br>8-isoprostane								

First author (year)	Country of study, Study name	Population description	Outcome measures	Sample size analyzed			Age in years (range or mean (SD))	Exposure			Conflict of interest
				Total	Females (N, %)	Males (N, %)		Comparison groups	ECU sub-groups	Exclusive ECU	
			8 hydroxydeoxyguanosine<br>Myeloperoxidase4-hydroxynonenal<br>Resolvin E1<br>Malondialdehyde								
(Sinha et al., 2021)[48]	India	Healthy adults. Recruitment is not described.	Inflammatory biomarkers:<br>PISF Volume<br><br>TNF-α<br>IL-1β	92	0, 0%	92, 100%	NS:<br>44.8 (2.5)<br><br>ECU<br>34.6 (6.1)	NS, ECU	Daily users	Yes	None
(Song, Freudenheim, et al., 2020)[49]	USA	Healthy adults who volunteered for bronchoscopy	Inflammatory biomarkers:<br><br>IFN-γ, IL-1β, IL-2, IL-4, IL-6, IL-8, IL-10, IL-12, IL-13, TNF-α	73	34, 47%	39, 53%	21-30	NS, TS, ECU	Daily users	No	None
(Stokes et al., 2021)[50]	USA, PATH Study Wave 1	Adults who participated in the PATH study and gave biological samples	Inflammatory biomarkers:<br><br>High-sensitivity C-reactive protein,<br>IL-6<br>sICAM-1<br>Fibrinogen<br>Biomarker of oxidative stress<br>Urinary 8-isoprostane	7130	NR	NR	18-65+	NS, TS, ECU	Occasional users	No	Yes

[48] Sinha et al. (2021): All immunological biomarkers were measured as a indicator of oral health/inflammation via PISF. Also check oral health
[49] Song et al. (2020): All immunological indicators were measured as an indicator of respiratory health/injury/inflammation
[50] Stokes et al. (2021): All immunological indicators were measured in the context of cardiovascular health

First author (year)	Country of study, Study name	Population description	Outcome measures	Sample size analyzed			Age in years (range or mean (SD))	Exposure			Conflict of interest
				Total	Females (N, %)	Males (N, %)		Comparison groups	ECU sub-groups	Exclusive ECU	
(Ye et al., 2020)[51]	USA	Healthy adults recruited in New York at a university-based dental setting	Inflammatory biomarkers IL-1β<br>Inflammatory mediators: PGE2, EN-RAGE, RAGE, MMP-9, S100A8, S100A9, Galectin-3, Uteroglobin/CC-10<br>Biomarkers of oxidative stress: Myeloperoxidase (MPO)<br>Immune Response: Indicator of tissue injury/repair: Serpine1/PAI-1<br>Growth factors BDNF, Basic-FGF, Beta-NGF, SCF, BMP-2, HGF, PDGF-AA, TGF-α, EGF, PGF, VEG	48	24, 50%	24, 50%	37.57<br>NS 35.67 (12.46)<br>TS 40.25 (15.96)<br>ECU 34.92 (11.45)<br>DU 39.42 (11.81)	NS, TS, ECU, DU	Not specified	No	None

[51] Ye et al. (2020) all immunological indicators were measured as indicators of oral health

Table S2.3: Oral health

First author (year)	Country of study, Study name	Population description	Outcome measures	Sample size analyzed			Age in years (range or mean (SD))	Exposure			Conflict of Interest
				Total	Females (N, %)	Males (N, %)		Comparison groups	ECU sub-groups	exclusive ECU	
**Oral health (n=23)**											
**Cohort studies (n=3)**											
(ALHarthi et al., 2019)	Saudi Arabia	Patients were recruited from a single outpatient dental clinic, and received a full-mouth ultrasonic scaling in the initial visit, and followed after 3 and 6 months.	Full-mouth plaque index (PI), BOP, CAL and PD measured at 6 sites per tooth, assessed at baseline, 3 and 6 months.<br><br>Periodontal inflammation: if ≥ 6 sites with a (PD) of 4 to 8 mm in the upper and lower jaws.<br><br>Numbers of missing teeth	89	89	0	25- 60	NS, TS, ECU	Daily users (for at least 1 year)	Yes	None
(Atuegwu et al., 2019)	USA	Participants responding to 3 waves (2013 to 2016) of the Population Assessment of Tobacco and Health study (PATH)	Gums disease, bone loss, or any periodontal disease	18, 289, 329 were daily ECU for 3 years	Longitudinal ECU cohort = 154 (46.8%)<br><br>and<br><br>NS= 5347 (55.6%)	Longitudinal ECU cohort = 175 (53.2%)<br><br>and<br><br>NS= 4274 (44.4%)	>=18	ECU, NS[52]	Daily users (for the 3 years of data collection).<br><br>and<br><br>Others reported daily or occasional use at	No	None

[52] Atuegwu et al NS: never ECU

First author (year)	Country of study, Study name	Population description	Outcome measures	Sample size analyzed			Age in years (range or mean (SD))	Exposure			Conflict of Interest
				Total	Females (N, %)	Males (N, %)		Comparison groups	ECU sub-groups	exclusive ECU	
		Participants were followed for 3 years, had no history of gum disease at baseline							baseline only		
(Ghazali et al., 2019)	Malaysia	Participants were recruited from a dental specialist clinic Kulliyyah of Denistry, and divided into three exposure groups (control, cigarette, and e-cigarette users). The groups were followed up with after 6 months.	Clinical oral examination of caries statuses, assessing decayed, missing and filled teeth.<br><br>Health Organization (WHO) "Decayed, Missing, Filled (DMFT) index is computed.<br><br>The index can range from 0 to 28 or 32	135 participants (45 per group) at baseline assuming 20% dropout	NS[53]: 29 (64.4%) TS 1 (0.7%) ECU (2 (4.4%)	NS: 16 (35.6%) TS: 44 (97.8%) ECU: 43 (95.6%)	20-64	NS, TS, ECU	Not stated/ Unclear	No	None
**Case-control studies** (n=2)											
(Ibraheem et al., 2020)	Saudi Arabia	Healthy male adults recruited from the	Receptor activator of nuclear factor-kB (RANK)	120	0	120, 100%	Overall: 45.4<br><br>NS:	NS, TS, ECU	Daily users	No	None

First author (year)	Country of study, Study name	Population description	Outcome measures	Sample size analyzed			Age in years (range or mean (SD))	Exposure			Conflict of Interest
				Total	Females (N, %)	Males (N, %)		Comparison groups	ECU sub-groups	exclusive ECU	
		outpatient department of the College of Dentistry, Jazan University.	Osteoprotegerin (OPG)<br>- as biomarkers of bone density losss<br><br>Clinical periodontal parameters: PI, PD, CAL, and MBL on the mesial and distal surfaces of the teeth.				43.8 (1.7)<br><br>TS:<br>46.5 (5.3)<br><br>ECU:<br>45.6 (3.6)				
(Karaaslan et al., 2020)[54]	Turkey	Participants were recruited from the Department of Periodontology of Usak University.	Clinical periodontal measurements (PI, GI, PD and CAL) obtained from 6 points around each tooth except 3rd molars.<br><br>Periodontitis diagnosed if interdental AL was detectable at ≥2 non-adjacent teeth; buccal or oral AL was ≥3 mm with pocketing >3 mm detectable at ≥2 teeth and observed AL	57<br><br>19 TS, 19 ECU and 19 former smokers	18 (31.6%)	39 (68.4%)	29-39	TS, ECU	Daily users, (for at least 12 months)	No	None

[54] Karaaslan et al. (2020): Also check immunological health.

First author (year)	Country of study, Study name	Population description	Outcome measures	Sample size analyzed			Age in years (range or mean (SD))	Exposure			Conflict of Interest
				Total	Females (N, %)	Males (N, %)		Comparison groups	ECU sub-groups	exclusive ECU	
			could not be attributed to non-periodontitis causes. (2017 World Workshop).								
**Cross-sectional studies (n=18)**											
(Aherrera et al., 2020)[55]	USA	Residents of Maryland, recruited through advertisements in universities, vaping shops conventions, local newspaper, online websites and social media.	Self reported dental discolouration, gingival inflammation.	150	91, 64%	59, 36%	30.1 (9.6)	ECU , NS	Daily users	No	Yes, none declared but the work was partially supported by the Maryland Cigarette Restitution Fund
(Akinkugbe, 2019)	USA	Adolescents responding to 2013-2014 wave of the Population Assessment of Tobacco and Health study (PATH)	Self-reported dental health issues, such as cavities, gum disease, or dental stains in the past year, or ever<br><br>Participants report on behalf of them selves or their child, as diagnosed by	13650	6657, 48.8%	6993, 51.2%	12-17	NS[56],<br><br>Current ECU (including ECU only or dual users),<br><br>ever users (ECU only or dual users	Occasional or daily users<br><br>Unclear (ever users)	No	None

[55] Aherrera et al. (2020): Also see in respiratory, cardiovascular, and immunological health.
[56]Akinkugbe et al. (2020): NS: non-users

First author (year)	Country of study, Study name	Population description	Outcome measures	Sample size analyzed			Age in years (range or mean (SD))	Exposure			Conflict of Interest
				Total	Females (N, %)	Males (N, %)		Comparison groups	ECU sub-groups	exclusive ECU	
			dentist or health professional.								
(Al Deeb et al., 2020)[57]	Saudi Arabia	Men with peri-implant mucositis (p-iM)	Plaque index (PI)<br><br>Probing depth (PD) (in millimetres)	71	0	71, 100%	30.2 (4.4)	NS, TS, ECU	Daily users (> one month, and every day use)	No	None
(Al-Aali et al., 2018)[58]	Saudi Arabia	Menrecruite d from a single clinic	Clinical peri-implant assessment (plaque index, BOP probing depth >=4 mm), and CAL<br><br>Radiographic evaluation of peri-implant bone loss: volume of PISF	92	0	92, 100%	ECU: 35.8 (6.2)<br>NS: 42.6 (2.7)	ECU and NS[59]	Daily users (at least one year)	No	None
(Aldakheel et al., 2020)	France	Men volunteers, including 15 Self-reported cigarette-smokers, 15 self-reported ENDS-users, and 30 self-reported non-smokers; with (n=15)	Full-mouth clinical attachment loss (CAL), gingival index (GI), plaque index (PI)	60	0	60, 100%	ECU: 38.6 (3.3),<br><br>TS 40.5 (2.1),<br><br>NS with periodontitis = 39.4 (1.6),<br><br>NS without periodontitis 39.5 (0.8)	ECU, TS (light smokers; < 10 cigarette/day), and NS.	Daily users (at least 5 cigarettes daily for the past 12-months).	No	None

[57]Al Deeb et al. (2020): Also check immunological health.
[58] Al-Aali et al. (2018): Also check immunological health.
[59] Al-Aali et al.(2018): NS are never smokers.

First author (year)	Country of study, Study name	Population description	Outcome measures	Sample size analyzed			Age in years (range or mean (SD))	Exposure			Conflict of Interest
				Total	Females (N, %)	Males (N, %)		Comparison groups	ECU sub-groups	exclusive ECU	
		and without (n=15) a diagnosis of periodontitis. All patients with periodontitis had Grade-B periodontitis									
(Al-Hamoudi et al., 2020)[60]	USA	Adults with chronic periodontitis, recruited to investigate the effect of scaling and root planning (SRP) on the expression of anti-inflammatory cytokines.	Baseline characteristics periodontal parameters (Plaque Index (PI), PD, CAL, and MBL)	71	9, 12.7%<br><br>4 ECU and 5 NS.	62, 87.3%<br><br>32 ECU and 30 NS	ECU= 47.7(5.8)<br><br>NS= 46.5±3.4 years	ECU and NS	Daily users (> one month)	No	None
(AlQahtani et al., 2018)[61]	Saudi Arabia	Men with dental implants.	Bleeding on probing (BOP), plaque index (PI), probing depth (PD)) and radiographic bone loss	160	0	160, 100%	41.8 (34-53)	TS, ECU (ENDS users), NS with periodontitis and NS without periodontitis	Daily users (at least once daily for the past 12 months)	Yes	None

[60] Al-Hamoudi et al. (2020): Also check immunological health
[61] AlQahtani et al. (2018): Also check immunological health

First author (year)	Country of study, Study name	Population description	Outcome measures	Sample size analyzed			Age in years (range or mean (SD))	Exposure			Conflict of Interest
				Total	Females (N, %)	Males (N, %)		Comparison groups	ECU sub-groups	exclusive ECU	
(F. Alqahtani et al., 2019)[62]	Saudi Arabia	Men with dental implants	Bleeding on probing (BoP), plaque index (PI) and probing depth (PD) assessed at six sites per implant.	102	0	102, 100%	TS: 36.3 (1.2), e-cig users: (33.5 (0.7), non-smokers: 32.2 (0.6)	TS, ECU, NS	Daily users (at least once daily for at least 1 year)	No	None
(ArRejaie, 2019)[63]	Saudi Arabia	Men enrolled from the department of prosthetic dental sciences, King Saud University	Clinical peri-implant assessment, plaque index (PI) bleeding on probing (BOP), probing depth (PD) and marginal bone loss (MBL)	95	0	95, 100%	TS: 40.4 (3.5), ECU: 35.8 (6.2), non smokers: 42.6 (2.7)	TS, ECU, NS	Daily users (for at least 1 year)	No	None
(BinShabaib et al., 2019)[64]	Saudi Arabia	Volunteers	Clinical assessment of probing depth (PD), bleeding on probing (POB), plaque index (PI), marginal bone loss.	135	11.8%	124, 92%	TS : 44.2 (3.5), ECU: 36.5 (1.7), and NS: 40.6 (3.3)	TS, ECU and NS	Daily users (for at least once daily)	No	None
(Huilgol et al., 2019)	USA	Participants who responded to the 2016 Behavioral	Poor oral health, determined by the number of permanent teeth removed due to	456,343	258,321, 56.6%	198,022, 43.4%	>=18	ECU, TS, and NS.	Daily and occasional users within the last 30 days	No	None declared. But two authors received

[62] AlQahtani et al. (2019): Also check immunological health
[63] ArRejaie et al. (2019): Also check immunological health
[64] BinShabaib et al. (2019): Also check immunological health

First author (year)	Country of study, Study name	Population description	Outcome measures	Sample size analyzed			Age in years (range or mean (SD))	Exposure			Conflict of Interest
				Total	Females (N, %)	Males (N, %)		Comparison groups	ECU sub-groups	exclusive ECU	
		Risk Factor Surveillance System (BRFSS)	non-traumatic causes, losing 1-5 teeth, 6 or more (but not all).<br><br>No teeth loss= 'good oral health"								consultants fees/ grants on projects related to pharmatheutical companies outside the submitted work.
(Javed et al., 2017)	Saudi Arabia	Participants recruited from the out-patient clinic of the College of Dentistry, King Saud University	Full-mouth plaque index (PI) , Bleeding on probing (BOP), clinical attachment loss (CAL), probing depth (PD) ‡4 mm, measured at six sites per tooth and marginal bone loss (MBL).<br><br>Self-perceived oral symptoms (gingival bleeding, pain, and swelling).	94	0	94, 100%	TS: 41.3 (2.8),<br><br>ECU 37.6 (2.1),<br><br>and NS 40.7 (1.6)	TS, ECU and NS	Daily users (at least once daily for 12 months	Yes	None
(Jeong et al., 2019)	South Korea	Individuals are a sample of the 2013-2015 Korean National Health and Nutrition Examination	Community periodontal index (CPI) score (0-4). **0:** Healthy periodontal tissue, **1:** Bleeding periodontal tissue, **2:** periodontal tissue with	13, 551	7836, 57.8%	5715, 42.2%	19 -60	ECU, TS, and NS	Unclear duration, ever users.	No	None

First author (year)	Country of study, Study name	Population description	Outcome measures	Sample size analyzed			Age in years (range or mean (SD))	Exposure			Conflict of Interest
				Total	Females (N, %)	Males (N, %)		Comparison groups	ECU sub-groups	exclusive ECU	
		Survey (KNHANES)	plaques, **3:**periodontal tissue with shallow periodontal pockets (3.5 ≤ pocket depth < 5.5 mm), and **4**: periodontal tissue with deep periodontal pockets (pocket depth ≥ 5.5 mm). Either 3 or 4 indicates periodontal disease.								
(Mokeem et al., 2018)	Saudi Arabia	Adults TS (n=39), ECU (n=37) and and NS (n=38). No details on recruitment method.	Clinical (PI, BOP, PPD and CAL) and radiographic periodontal parameters (mesial and distal MBL).	154	0	154, 100%	TS: (42.4 ± 5.6); ECU: (28.3 ± 3.5); NS[65] (40.6 ± 4.5)	TS, ECU, and NS,	Daily users (at least 12 months, and never smoked tobacco in the past	Yes	None
(Mokeem et al., 2019)	Saudi Arabia	TS, ECU, and NS[66] recruited from the outpatient department of	Number of missing teeth.	129	0	129, 100%	TS: (33.2 ± 8.6); ECU: (29.4 ± 4.5); NS (32.5 ± 5.4)	TS, ECU, and NS	Daily users (vaping at least 6 times daily since 12 months	Yes	None

[65] Mokeem et al. (2018): NS: never smokers
[66] Mokeem et al. (2019): NS: never smokers

First author (year)	Country of study, Study name	Population description	Outcome measures	Sample size analyzed			Age in years (range or mean (SD))	Exposure			Conflict of Interest
				**Total**	**Females** (N, %)	**Males** (N, %)		**Comparison groups**	**ECU sub-groups**	**exclusive ECU**	
		a local University-based dental clinic in Riyadh.									
(Sinha et al., 2021)[67]	India	Healthy ECU and NS with a minimum of one dental implant (functional for 3 years), and no periodontal therapy, nor use of antibiotic in the last 6 months.	Clinical parameters including PI, BOP, and PD and radiographic evaluation peri-implant bone loss (PIBL)	92	0	92, 100%	ECU: 34.6(6) and NS: 44.8(2.5)	ECU and NS	Daily users for a minimum of 1 year	No	None
(Vohra et al., 2020)	Saudi Arabia	Men, recruitment method was not reported	Self -rated oral symptoms: Pain in gum, and bleeding gums.<br><br>Periodontal parameters: clinical and radiographic examination: number of missing teeth, PI, BOP, PD (in	105	0	105, 100%	33.5 (1.4)	TS, ECU, JUUL users, and NS[68]	Daily users (daily users for approximatel y a year use	No	None

[67] Sinha et al. (2021): Also check immunological health
[68] Vohra et al. (2020): NS are never smokers

First author (year)	Country of study, Study name	Population description	Outcome measures	Sample size analyzed			Age in years (range or mean (SD))	Exposure			Conflict of Interest
				Total	Females (N, %)	Males (N, %)		Comparison groups	ECU sub-groups	exclusive ECU	
			mm), CAL (in mm), MBL mesial (in mm) and distal (in mm).								
(Vora & Chaffee, 2019)	USA	Adults who responded to the PATH study (a nationally representative longitudinal study)	Self-reported gingival disease	32,320	NS : 35.8%<br><br>TS: 49.9%<br><br>ECU: 44.8%	NS 64.2% ;<br><br>TS: 50.1% ;<br><br>ECU 55.2%	≥ 18	NS[69], TS, ECU	Daily or occasional users	No	None

Table S2.4: Respiratory heath

First author (year)	Country of study, Study name	Population description	Outcome measures	Sample size analyzed			Age in years (range or mean (SD))	Exposure			Conflict of Interest
				Total	Female (N, %)	Male (N, %)		Comparison groups	ECU sub-groups	exclusive ECU	
**Respiratory health** (n =32)											
**Randomized control trials (n=4)**											
(Haziza et al., 2020)[70]	USA	Healthy adults daily smokers (22yrs+)	FEV1	160	96, 60%	64, 40%	22–66<br><br>37.7 (11.45)	NS, ECU, TS	Daily users	No	Yes
(Ludicke et al., 2018)[71]	Switzerland	Healthy Japanese adults	FEV1<br><br>FVC<br><br>FEV1/FVC	160	NR	NR	23-65	NS, ECU, TS	Daily users	No	Yes

[69] Vora & Chaffe (2019): NS are never users.
[70] Haziza (2020): Also check cardiovascular, and immunological health
[71] Ludick (2020): Also check immunological health section

First author (year)	Country of study, Study name	Population description	Outcome measures	Sample size analyzed			Age in years (range or mean (SD))	Exposure			Conflict of Interest
				**Total**	**Female** (N, %)	**Male** (N, %)		**Comparison groups**	**ECU sub-groups**	**exclusive ECU**	
(Ludicke et al., 2019)[72]	Switzerland	Healthy adult volunteers who were not motivated to quit smoking within the next 6 months.	FEV1, FEV1%pred<br><br>Cough	803	476, 59%	339, 41%	Overall: 30+<br>ECU:<br>44.2 (9.64),<br>DU:<br>43.8 (9.77),<br>TS:<br>45.2 (9.55)	ECU, TS, DU	Daily users	No	Yes
(Pulvers et al., 2020)[73]	USA	Healthy adults from several US cities participating in a smoking cessation program, and identifying as African American or Latinos	MFEF25%-75%<br><br>Grouped respiratory symptoms (summative ATSQ score measuring coughing, wheezing, phlegm)	186	111, 60%	75, 40%	43.3 (12.5)	ECU, TS, DU	Daily users	No	Yes
**Cohort studies (n=5)**											
(Dai & Khan, 2020)	USA, Path Study Waves 1-2	Nationally representative US civilians (18yrs+). ECU information was collected during Wave 1 (September 2013 = december 2014) and respiratory symptoms collected during Wave 2 (October	Grouped Respiratory Symptoms (cough and/or wheeze)	4614	2178, 41%	2436, 59%	18-55+	NS, ECU, DU	Daily or occasion al users	No	None

[72] Ludick (2019): Also check immunological and cardiovascular health
[73] Pulvers (2020): Also check in cardiovascular health

First author (year)	Country of study, Study name	Population description	Outcome measures	Sample size analyzed			Age in years (range or mean (SD))	Exposure			Conflict of Interest
				Total	Female (N, %)	Male (N, %)		Comparison groups	ECU sub-groups	exclusive ECU	
		2014 -October 2015).									
(Polosa et al., 2020)	Italy, N/R	Patients diagnosed with COPD, being treated for symptoms, and reported daily ECU. Datasets from age- and sex-matched COPD daily TS patients. Groups were followed up at the 60-month (5 yr) and compared to baseline.	Grouped Respiratory Symptoms (COPD exacerbation s)<br><br>CAT score<br><br>FEV1<br><br>FVC<br><br>FEV1/FVC	39	33, 85%	6, 15%	ECU+ DU 66.9 (5.8) at baseline<br><br>TS 65.0 (5.7) at baseline	ECU, TS	Daily users	No	Yes
(Polosa et al., 2018)	Italy, N/R	Adults with COPD were recruited by physicians at participating health clinics at four Italian hospitals (September 2013 - december 2015) in the outpatient setting. Groups were followed up at the 36-month (3 yr) and compared to baseline.	Respiratory Symptoms (COPD exacerbation s & CAT score)<br><br>FEV1<br><br>FVC<br><br>FEV1/FVC	44	37, 84%	7, 16%	COPD controls 65.2 (5.6)<br><br>COPD E-Cigarette users 66.5 (6.8)	ECU<br><br>TS	Daily users	No	Yes
(Polosa et al., 2017)	Italy, N/R	Adult vape users who frequented affiliated vape shops were invited They were	FEV1<br><br>FVC<br><br>FEV1/FVC	31	21, 68%	10, 32%	ECU 29.7 (6.1)<br><br><br>NS	NS<br><br>ECU	Daily users	Yes	Yes

First author (year)	Country of study, Study name	Population description	Outcome measures	Sample size analyzed			Age in years (range or mean (SD))	Exposure			Conflict of Interest
				Total	Female (N, %)	Male (N, %)		Comparison groups	ECU sub-groups	exclusive ECU	
		invited for a free medical check-up at the Centro per la Prevenzione e Cura del Tabagismo (CPCT) of the University of Catania. Age- and sex-matched non-smoking controls (and not using ECs) were selected from hospital staff and included as a controls. Groups were followed up at the 42-month (3.5 yr) mark and compared from baseline	MFEF25%-75%<br><br>FeNO<br><br>CO<br><br>Cough<br><br>Wheeze<br><br>Shortness of breath<br><br>Chest tightness				32.5 (7.0)				
(Tackett et al., 2020)	USA, PATH Study Wave 3 & 4	Healthy adolescents responding to a nationally representative survey, USA. ECU was measured at Wave 3 (October 2015-October 2016), and wheezing was measured at Wave 4 (December 2016 - January 2018).	Wheezing	7049	3532, 50%	3517, 50%	12-17	NS, ECU	Occasion al users<br><br>Not Specified (12 month use)	No	None

First author (year)	Country of study, Study name	Population description	Outcome measures	Sample size analyzed			Age in years (range or mean (SD))	Exposure			Conflict of Interest
				Total	Female (N, %)	Male (N, %)		Comparison groups	ECU sub-groups	exclusive ECU	
**Cross-Sectional (n=23)**											
(AboElNaga, 2018)	Egypt	Adults diagnosed with asthma	Forced vital capacity (FVC),<br><br>forced expiratory volume (FEV1),<br><br>FEV1/FVC ratio,<br><br>maximal mid expiratory flow (MFEF25%-75%),<br><br>peak expiratory flow rate (PEFR),<br><br>grouped respiratory symptoms (asthma control test (act score; measures asthma symptoms as summative score)	130	63, 48%	67, 52%	NS: 30.29 (4.89),<br><br>TS : 29.46 (5.34),<br><br>ECU: 30.41 (4.73)	NS, TS, ECU	Daily users	No	None

First author (year)	Country of study, Study name	Population description	Outcome measures	Sample size analyzed			Age in years (range or mean (SD))	Exposure			Conflict of Interest
				Total	Female (N, %)	Male (N, %)		Comparison groups	ECU sub-groups	exclusive ECU	
(Aherrera et al., 2020)[74]	USA	Adults residing in Maryland, ECU recruited through advertisements and flyers posted in universities, local newspapers and online advertisement websites social media platforms, and e-cigarette shops and conventions.	Asthma<br><br>respiratory disease,<br><br>wheezing,<br><br>shortness of breath,<br><br>coughing,<br><br>phlegm.	150	91, 64%	59, 36%	18+<br><br>30.1 (9.6)	NS, ECU	Daily users	No	Yes
(Alnajem et al., 2020)	Kuwait	Young adults, grades 11 & 12, attending public high-schools in Kuwait from January to May 2019	Wheezing,<br><br>grouped respiratory symptoms (severe asthma symptoms (≥ 4 wheezing attacks, sleep disturbance from wheezing ≥ 1 night per week, and/or wheezing-affected speech in the	1345	648, 48%	697, 52%	16-19	NS, ECU, DU	Occasion al users<br><br>Former users	Yes	None

[74] Aherrera (2020): Also check oral, cardiovascular, and immunological health

**First author (year)**	**Country of study, Study name**	**Population description**	**Outcome measures**	**Sample size analyzed**			**Age in years** (range or mean (SD))	**Exposure**			**Conflict of Interest**
				**Total**	**Female** (N, %)	**Male** (N, %)		**Comparison groups**	**ECU sub-groups**	**exclusive ECU**	
			past 12 months))								
(Ashford et al., 2020)[75]	USA	Convenience sample collected from English speaking undergraduate college students on campus at the University of Kentucky.	Cough	61	29, 47%	32, 53%	18-25	NS, ECU	Occasion al users	No	None
(Boddu et al., 2019)	USA	Adults recruited through social media to participate in a nationwide online survey	Cough	273	134, 49%	139, 51%	30.2 (12.3)	NS, ECU, TS, DU	Not specified	No	None
(Bowler et al., 2017)	United States, COPDGene Study & SPIROMICS Study	The sample consisted of non-Hispanic white or African American (CODPGene) adults with a history of at least 10 pack years of CC, or no smoking, who participated in our of two prospective cohorts (COPD Gene and SPIROMICS). COPDGene collected	The Global Initiative for Chronic Obstructive Lung Disease (GOLD Criteria (FEV1/FVC; FEV1%)<br><br>FEV1<br><br>Chronic Bronchitis	COPD Gene: 3435<br><br>SPIRO MICS 1060	COPDGe ne Never use 51% Current use 41% Former use 43%<br><br>SPIROM ICS Never use 54% Current use	NR	Age Range 40-80	Traditional Smokers<br><br>E-cigarette Smokers	Regular or Occasion al Use	Yes	None

[75] Ashford (2020): Also see in immunological Health

First author (year)	Country of study, Study name	Population description	Outcome measures	Sample size analyzed			Age in years (range or mean (SD))	Exposure			Conflict of Interest
				**Total**	**Female** (N, %)	**Male** (N, %)		**Comparison groups**	**ECU sub-groups**	**exclusive ECU**	
		information from 2008-2011, and SPIROMICS collected information from 2011-2015.			55% Former use 44%						
(Braymiller et al., 2020)	USA, Happiness and Health Study	Adolescents recruited from Southern California high-school (June 2018 to October 2019).	Grouped respiratory symptoms (Bronchitic symptoms (daily cough for 3 months in a row, congestion or phlegm other than with a cold, and/or bronchitis), wheeze, and shortness of breath.)	2553	1076, 42%	1477, 58%	19.3 (0.79)	NS, ECU	Occasional users<br><br>And<br><br>Not specified (lifetime)	No	None
(Brozek et al., 2019)	Poland, Young People E-Smoking Study	Healthy young adults recruited from a single site in Katowice.	FEV1, FVC, FEV1/FVC, PEF, MFEF25%-75%,<br><br>O2 saturation	120	71, 59%	49, 40%	21.7 (2.1)	NS, TS, ECU, DU	Not specified	No	None
(Cassidy et al., 2020)	USA	Adults (18-60yrs) recruited via flyers, bus ads, online and social media posts, and in-person methods at local vape	Wheezing,<br><br>grouped respiratory symptoms (ATSQ Summative	113	61, 54%	52, 46%	31.0 (11.0)	ECU and DU	Daily and occasional users	No	None

First author (year)	Country of study, Study name	Population description	Outcome measures	Sample size analyzed			Age in years (range or mean (SD))	Exposure			Conflict of Interest
				**Total**	**Female** (N, %)	**Male** (N, %)		**Comparison groups**	**ECU sub-groups**	**exclusive ECU**	
		shops (July 2016 - July 2018).	Score: cough first thing in morning, cough throughout day, wheezing, shortness of breathe when walking/during exercise, phlegm or mucous when coughing, pain/tightness in chest)								
(Diamantopoulou et al., 2019)	Greece,	Adults recruited from Athens vape-shop customers.	Difficulties breathing (perceived changes in breathing), cough, dyspnea (shortness of breath)	309	215, 70%	92, 30%	36.3 (11.9)	ECU, DU	Daily or occasional users	No	None
(Ghosh et al., 2019)[76]	USA	Healthy adults	FEV1, FVC, protein outcomes (BAL Protease levels)	42	22, 52%	20, 48%	NS: 25.79 (7.29), TS: 29.50 (5.59), ECU: 26.07 (8.30)	NS, TS, ECU	Daily users	No	None

[76] Ghosh A (2019): Also check in immunological heath

First author (year)	Country of study, Study name	Population description	Outcome measures	Sample size analyzed			Age in years (range or mean (SD))	Exposure			Conflict of Interest
				Total	Female (N, %)	Male (N, %)		Comparison groups	ECU sub-groups	exclusive ECU	
(Giovanni et al., 2020)	USA, BRFSS (2017)	Non-institutionalized adults.	Grouped Respiratory symptoms (cough, sputum production, or breathlessness during the past 3 months), cough, sputum, shortness of breath.	87067	41728, 48%	45339, 52%	18-55+	NS, TS, ECU, DU	Daily or occasional users	Yes	None
(Hedman et al., 2018)	Sweden, Obstructive Lung Disease in Northern Sweden (OLIN) study and the West Sweden Asthma Study(WSAS)	Adults random sample who responded to two surveys, in 2 large geographical areas of Sweden, Norrbotten and Västra Götaland.	Cough, sputum production, wheezing	30272	NR, 46%	NR, 54%	20-75	NS, ECU, DU	Daily or occasional users	Yes	Yes
(Kizhakke Puliyakote et al., 2020)	USA	Volunteer healthy adults (free of cardiovascular or pulmonary disease)	Mean alveolar ventilation & perfusion,<br><br>SpO2, peripheral oxygen saturation,	16	10, 63%	6, 37%	NS 23 (5), ECU 21 (2)	NS, ECU, DU (Hookah + E-Cig)	Daily users	No	None

**First author (year)**	**Country of study, Study name**	**Population description**	**Outcome measures**	**Sample size analyzed**			**Age in years** (range or mean (SD))	**Exposure**			**Conflict of Interest**
				**Total**	**Female** (N, %)	**Male** (N, %)		**Comparison groups**	**ECU sub-groups**	**exclusive ECU**	
			FVC, FEV1, FEV1/FVC								
(Li & Xie, 2020)	USA, PATH Study (2016-2018)	Adults who responded to Wave 4 of the PATH Study, a nationally representative study.	Wheezing, coughing	21976	NR	NR	18-65+	NS[77], ECU	Not Specified (Lifetime Puffs)	No	None
(Li et al., 2019)	USA, PATH Study (2014-2015; Wave 2)	Adult who responded to the 2nd Wave of the PATH study, a nationally representative study.	Wheezing, coughing	28171	Total: NR<br><br>Current ECU: 58.52%,<br><br>TS: 54.81%,<br><br>DU: 54.00%,<br><br>NS: 46.18%	Total: NR<br><br>Current ECU 41.48%<br><br>TS, 45.19%.<br><br>DU: 46.00%<br><br>NS: 53.82%	18+	NS[78], ECU, TS, DU	Daily or occasion al users	Yes	Yes
(Meo et al., 2019)	Saudi Arabia	Healthy voluntary participants were recruited on campus and matched to controls, from the Department of	FVC<br>FEV1<br>FEV1/FVC<br>PEF<br><br>MFEF25%-75%	60	60, 100%	0, 0%	Group 1<br>27.07 (6.00)<br><br>Group 2<br>25.90 (7.72)	NS, ECU	Daily users	Yes	None

[77] Li & Xie: NS is stated as never ECU users.
[78] Li et al.: NS is stated as non-ECU users.

First author (year)	Country of study, Study name	Population description	Outcome measures	Sample size analyzed			Age in years (range or mean (SD))	Exposure			Conflict of Interest
				Total	Female (N, %)	Male (N, %)		Comparison groups	ECU sub-groups	exclusive ECU	
		Physiology, College of Medicine, King Saud University, Riyadh, Saudi Arabia.									
(Perez et al., 2020)[79]	USA	Adults TS (n=23 mean age 39.5 (48.0), ECU (n=22) and NS (n=20).	Sputum FEV1, FVC, FEV1/FVC ratio	65	41, 63%	24, 37%	18-55<br><br>NS<br>28.0 (95%CI 25.5-34.0)<br><br>ECU<br>24.0 (95%CI 20.3-28.0)<br><br>TS:<br>39.5 (95%CI 30.5-48.0)	NS, ECU	Daily users	No	None
(Sakaguchi et al., 2021)[80]	Japan	Healthy adults who took part in a three-group, multicentre study recruited by 3H Medi Solution Inc.<br>.	FEV1<br>FVC,<br>MFEF25-75,<br><br>FEV1/FVC ratio,<br>PEF, cough.	459	343, 75%	116, 25%	Overall: 45.4 (9.3)<br><br>NS:<br>44.6 (8.7),<br><br>ECU: 45.4 (9.4),<br><br>TS:<br>45.9 (9.7),	NS, TS, ECU	Daily users	No	None
(Schneller et al., 2020)	USA, PATH Study	Adults who participated in the 3rd Wave of the PATH study	Wheezing	28082	13747, 49%	14310, 51%	18-55+	NS, TS, ECU	Daily or occasional users	Yes	Yes

[79] Perez (2020): Also check immunological health
[80] Sakaguchi et al.(2021): Also check immunological, and cardiovascular health

First author (year)	Country of study, Study name	Population description	Outcome measures	Sample size analyzed			Age in years (range or mean (SD))	Exposure			Conflict of Interest
				Total	Female (N, %)	Male (N, %)		Comparison groups	ECU sub-groups	exclusive ECU	
(Singh et al., 2019)[81]	USA	Adults recruited through the General Clinical Research Center of the University of Rochester Medical Center through various local newspaper and magazine advertisements, word of mouth, and flyers posted in and around the university campus.	FEV1, FEV1 % pred, FVC%, PEF FEV1/FVC	48	21, 44%	27, 56%	21-65<br>TS 33.9 (14.1)<br>ECU: 35.5 (12.2)	NS<br>ECU	Not specified	No	None
(Wang et al., 2018)[82]	USA, Health eHeart Study	Adults (18yrs+) who participates in the Health eHeart Study from March 2013-2017 and recruited worldwide via the lay press, promotional events, word-of-mouth, social media, e-mail, and clinic visits.	Difficulty breathing	39747	12047, 30%	27600, 69%	21.15 (2.55)	ECU, DU	Daily or occasional users	No	None

[81] Singh et al. (2019): Also check immunological health
[82] Wang JB et al.((2019): Also check cardiovascular health

First author (year)	Country of study, Study name	Population description	Outcome measures	Sample size analyzed			Age in years (range or mean (SD))	Exposure			Conflict of Interest
				Total	Female (N, %)	Male (N, %)		Comparison groups	ECU sub-groups	exclusive ECU	
(Xie et al., 2020)	USA, PATH Study (4 Waves)	Adults who responded to the 4th wave of the PATH Study, a nationally representative survey.	Wheezing Cough	33822	16635, 49%	16967, 50%	18-65+	NS, ECU	Not specified (ever use)	No	None

References of studies included in this Systematic Review: (N=93)

AboElNaga, H. H. (2018). Electronic cigarettes: Not an advantageous alternative to conventional smoking in asthma. *Egyptian Journal of Chest Diseases and Tuberculosis*, *67*(4), 427–432. https://doi.org/http://dx.doi.org/10.4103/ejcdt.ejcdt_83_18

Aherrera, A., Aravindakshan, A., Jarmul, S., Olmedo, P., Chen, R., Cohen, J. E., Navas-Acien, A., & Rule, A. M. (2020). E-cigarette use behaviors and device characteristics of daily exclusive e-cigarette users in Maryland: Implications for product toxicity. *Tobacco Induced Diseases*, *18*(Journal Article PG-93), 93. https://doi.org/https://dx.doi.org/10.18332/tid/128319

Akinkugbe, A. A. (2019). Cigarettes, E-cigarettes, and Adolescents' Oral Health: Findings from the Population Assessment of Tobacco and Health (PATH) Study. *JDR Clinical and Translational Research*, *4*(3 PG-276–283), 276–283. https://doi.org/https://dx.doi.org/10.1177/2380084418806870

Al-Aali, K. ., ArRejaie, A., Abduljabbar, T., Vohra, F., & Akram, Z. (2018). Peri-implant parameters, tumor necrosis factor-alpha, and interleukin-1 beta levels in vaping individuals. *Clinical Implant Dentistry and Related Research*, *20*(3), 410–415. https://doi.org/http://dx.doi.org/10.1111/cid.12597

Al-Hamoudi, N., Alsahhaf, A., Al Deeb, M., Alrabiah, M., Vohra, F., & Abduljabbar, T. (2020). Effect of scaling and root planing on the expression of anti-inflammatory cytokines (IL-4, IL-9, IL-10, and IL-13) in the gingival crevicular fluid of electronic cigarette users and non-smokers with moderate chronic periodontitis. *Journal of Periodontal & Implant Science*, *50*(2 PG-74–82), 74–82. https://doi.org/https://dx.doi.org/10.5051/jpis.2020.50.2.74

Al Deeb, M., Alresayes, S., Mokeem, S. A., Alhenaki, A., AlHelal, A., Shafqat, S., Vohra, F., & Abduljabbar, T. (2020). Clinical and immunological peri-implant parameters among cigarette and electronic smoking patients treated with photochemotherapy: A randomized controlled clinical trial. *Photodiagnosis and Photodynamic Therapy*, *31*(Journal Article), 101800. https://doi.org/http://dx.doi.org/10.1016/j.pdpdt.2020.101800

Aldakheel, F. M., Alduraywish, S. A., Jhugroo, P., Jhugroo, C., & Divakar, D. D. (2020). Quantification of pathogenic bacteria in the subgingival oral biofilm samples collected from cigarette-smokers, individuals using electronic nicotine delivery systems and non-smokers with and without periodontitis. *Archives of Oral Biology*, *117*(Journal Article),

104793. https://doi.org/http://dx.doi.org/10.1016/j.archoralbio.2020.104793

ALHarthi, S. S., BinShabaib, M., Akram, Z., Rahman, I., Romanos, G. E., & Javed, F. (2019). Impact of cigarette smoking and vaping on the outcome of full-mouth ultrasonic scaling among patients with gingival inflammation: a prospective study. *Clinical Oral Investigations*, *23*(6 PG-2751–2758), 2751–2758. https://doi.org/https://dx.doi.org/10.1007/s00784-018-2725-2

Alnajem, A., Redha, A., Alroumi, D., Alshammasi, A., Ali, M., Alhussaini, M., Almutairi, W., Esmaeil, A., & Ziyab, A. H. (2020). Use of electronic cigarettes and secondhand exposure to their aerosols are associated with asthma symptoms among adolescents: a cross-sectional study. *Respiratory Research*, *21*(1), 300. https://doi.org/http://dx.doi.org/10.1186/s12931-020-01569-9

Alqahtani, F., Alqahtani, M., Albaqawi, A. H., Al-Kheraif, A., & Javed, F. (2019). Comparison of cotinine levels in the peri-implant sulcular fluid among cigarette and waterpipe smokers, electronic-cigarette users, and nonsmokers. *Clinical Implant Dentistry and Related Research*, *21*(4 PG-702–707), 702–707. https://doi.org/https://dx.doi.org/10.1111/cid.12813

AlQahtani, M. A., Alayad, A. S., Alshihri, A., Correa, F. O. B., & Akram, Z. (2018). Clinical peri-implant parameters and inflammatory cytokine profile among smokers of cigarette, e-cigarette, and waterpipe. *Clinical Implant Dentistry and Related Research*, *20*(6), 1016–1021. https://doi.org/http://dx.doi.org/10.1111/cid.12664

Alqahtani, S., Cooper, B., Spears, C. A., Wright, C., & Shannahan, J. (2020). Electronic nicotine delivery system-induced alterations in oral health via saliva assessment. *Experimental Biology and Medicine*, *245*(15), 1319–1325. https://doi.org/http://dx.doi.org/10.1177/1535370220941258

Alzahrani, T., Pena, I., Temesgen, N., & Glantz, S. A. (2018). Association Between Electronic Cigarette Use and Myocardial Infarction. *American Journal of Preventive Medicine*, *55*(4), 455–461. https://doi.org/http://dx.doi.org/10.1016/j.amepre.2018.05.004

Arastoo, S., Haptonstall, K. P., Choroomi, Y., Moheimani, R., Nguyen, K., Tran, E., Gornbein, J., & Middlekauff, H. R. (2020). Acute and chronic sympathomimetic effects of e-cigarette and tobacco cigarette smoking: Role of nicotine and non-nicotine constituents. *American Journal of Physiology - Heart and Circulatory Physiology*, *319*(2), H262–H270. https://doi.org/http://dx.doi.org/10.1152/ajpheart.00192.2020

ArRejaie, A. S. (2019). Proinflammatory cytokine levels and peri-implant parameters among cigarette smokers, individuals vaping electronic cigarettes, and non-smokers. *Journal of Periodontology*, *90*(4), 367–374. https://doi.org/http://dx.doi.org/10.1002/JPER.18-0045

Ashford, K., McCubbin, A., Rayens, M. K., Wiggins, A., Dougherty, K., Sturgill, J., & Ickes, M. (2020). ENDS use among college students: Salivary biomarkers and persistent cough. *Addictive Behaviors*, *108*(Journal Article), 106462. https://doi.org/http://dx.doi.org/10.1016/j.addbeh.2020.106462

Atuegwu, N. C., Perez, M. F., Oncken, C., Thacker, S., Mead, E. L., & Mortensen, E. M. (2019). Association between regular electronic nicotine product use and self-reported periodontal disease status: Population assessment of tobacco and health survey. *International Journal of Environmental Research and Public Health*, *16*(7), 1263. https://doi.org/http://dx.doi.org/10.3390/ijerph16071263

Badea, M., Gaman, L., Delia, C., Ilea, A., Leasu, F., Henriquez-Hernandez, L. A., Luzardo, O. P., Radoi, M., & Rogozea, L. (2019). Trends of lipophilic, antioxidant and hematological parameters associated with conventional and electronic smoking habits in middle-age Romanians. *Journal of Clinical Medicine*, *8*(5), 665. https://doi.org/http://dx.doi.org/10.3390/jcm8050665

BinShabaib, M., ALHarthi, S. S., Akram, Z., Khan, J., Rahman, I., Romanos, G. E., & Javed, F. (2019). Clinical periodontal status and gingival crevicular fluid cytokine profile among cigarette-smokers, electronic-cigarette users and never-smokers. *Archives of Oral Biology*, *102*(Journal Article PG-212-217), 212–217. https://doi.org/https://dx.doi.org/10.1016/j.archoralbio.2019.05.001

Boddu, S. A., Bojanowski, C. M., Lam, M. T., Advani, I. N., Scholten, E. L., Sun, X., Montgrain, P., Malhotra, A., Jain, S., & Crotty, A. L. (2019). Use of E-cigarettes with Conventional Tobacco is Associated with Decreased Sleep Quality in Women. *American Journal of Respiratory and Critical Care Medicine*, *Journal Article*. https://doi.org/http://dx.doi.org/10.1164/rccm.201904-0890LE

Bowler, R. P., Hansel, N. N., Jacobson, S., Graham, B. R., Make, B. J., Han, M. L. K., O'Neal, W. K., Oelsner, E. C., Casaburi, R., Barjaktarevic, I., Cooper, C., Foreman, M., Wise, R. A., DeMeo, D. L., Silverman, E. K., Bailey, W., Harrington, K. F., Woodruff, P. G., & Drummond, M. B. (2017). Electronic Cigarette Use in US Adults at Risk for or with COPD: Analysis from Two Observational Cohorts. *Journal of General Internal Medicine*, *32*(12), 1315–1322. https://doi.org/http://dx.doi.org/10.1007/s11606-017-4150-7

Braymiller, J. L., Barrington-Trimis, J. L., Leventhal, A. M., Islam, T., Kechter, A., Krueger, E. A., Cho, J., Lanza, I., Unger, J. B., & McConnell, R. (2020). Assessment of Nicotine and Cannabis Vaping and Respiratory Symptoms in Young Adults. *JAMA Network Open*, *Journal Article*. https://doi.org/http://dx.doi.org/10.1001/jamanetworkopen.2020.30189

Brozek, G. M., Jankowski, M., & Zejda, J. E. (2019). Acute respiratory responses to the use of e-cigarette: an intervention study. *Scientific Reports*, *9*(1 PG-6844), 6844. https://doi.org/https://dx.doi.org/10.1038/s41598-019-43324-1

Cassidy, R. N., Tidey, J. W., & Colby, S. M. (2020). Exclusive E-Cigarette Users Report Lower Levels of Respiratory Symptoms Relative to Dual E-Cigarette and Cigarette Users. *Nicotine & Tobacco Research : Official Journal of the Society for Research on Nicotine and Tobacco*, *22*(1), S54–S60. https://doi.org/http://dx.doi.org/10.1093/ntr/ntaa150

Cichonska, D., Kusiak, A., Kochanska, B., Ochocinska, J., & Swietlik, D. (2019). Influence of electronic cigarettes on selected antibacterial properties of saliva. *International Journal of Environmental Research and Public Health*, *16*(22), 4433. https://doi.org/http://dx.doi.org/10.3390/ijerph16224433

Dai, H., & Khan, A. S. (2020). A longitudinal study of exposure to tobacco-related toxicants and subsequent respiratory symptoms among U.S. adults with varying e-cigarette use status. *Nicotine and Tobacco Research*, *22*, S61–S69. https://doi.org/10.1093/NTR/NTAA180

Diamantopoulou, E., Barbouni, A., Merakou, K., Lagiou, A., & Farsalinos, K. (2019). Patterns of e-cigarette use, biochemically verified smoking status and self-reported changes in health status of a random sample of vapeshops customers in Greece. *Internal and Emergency Medicine*, *14*(6 PG-843–851), 843–851. https://doi.org/https://dx.doi.org/10.1007/s11739-018-02011-1

Faridoun, A., Sultan, A. S., Jabra-Rizk, M. A., Weikel, D., Varlotta, S., & Meiller, T. F. (2021). Salivary biomarker profiles in E-cigarette users and conventional smokers: A cross-sectional study. *Oral Diseases*, *27*(2), 277–279. https://doi.org/http://dx.doi.org/10.1111/odi.13533

Farsalinos, K. E., Polosa, R., Cibella, F., & Niaura, R. (2019). Is e-cigarette use associated with coronary heart disease and myocardial infarction? Insights from the 2016 and 2017 National Health Interview Surveys. *Therapeutic Advances in Chronic Disease*, *10*(Journal Article). https://doi.org/http://dx.doi.org/10.1177/2040622319877741

Fetterman, J. L., Keith, R. J., Palmisano, J. N., McGlasson, K. L., Weisbrod, R. M., Majid, S., Bastin, R., Stathos, M. M., Stokes, A. C., Robertson, R. M., Bhatnagar, A., & Hamburg, N. M. (2020). Alterations in Vascular Function Associated With the Use of Combustible and Electronic Cigarettes. *Journal of the American Heart Association*, *9*(9), e014570. https://doi.org/http://dx.doi.org/10.1161/JAHA.119.014570

Ganesan, S. M., Dabdoub, S. M., Nagaraja, H. N., Scott, M. L., Pamulapati, S., Berman, M. L., Shields, P. G., Wewers, M. E., & Kumar, P. S. (2020). Adverse effects of electronic cigarettes on the disease-naive oral microbiome. *Science Advances*, *6*(22). https://doi.org/http://dx.doi.org/10.1126/sciadv.aaz0108

Gavrilin, M. A., McAndrew, C. C., Prather, E. R., Tsai, M., Spitzer, C. R., Song, M. A., Mitra, S., Sarkar, A., Shields, P. G., Diaz, P. T., & Wewers, M. D. (2020). Inflammasome Adaptor ASC Is Highly Elevated in Lung Over Plasma and Relates to Inflammation and Lung Diffusion in the Absence of Speck Formation. *Frontiers in Immunology*, *11*(Journal Article), 461. https://doi.org/http://dx.doi.org/10.3389/fimmu.2020.00461

George, J., Hussain, M., Vadiveloo, T., Ireland, S., Hopkinson, P., Struthers, A. D., Donnan, P. T., Khan, F., & Lang, C. C. (2019). Cardiovascular Effects of Switching From Tobacco Cigarettes to Electronic Cigarettes. *Journal of the American College of Cardiology*, *74*(25), 3112–3120. https://doi.org/http://dx.doi.org/10.1016/j.jacc.2019.09.067

Ghazali, A. F., Ismail, A. F., & Daud, A. (2019). Caries experience among cigarette and E-cigarette users: A 6-month prospective study. *Journal of Pharmaceutical Sciences and Research*, *11*(7), 2566–2569. http://www.jpsr.pharmainfo.in/Documents/Volumes/vol11issue07/jpsr11071915.pdf; http://ovidsp.ovid.com/ovidweb.cgi?T=JS&PAGE=reference&D=emexc&NEWS=N&AN=2002387403

Ghosh, A., Coakley, R. D., Ghio, A. J., Muhlebach, M. S., Esther, C. R., Alexis, N. E., & Tarran, R. (2019). Chronic E-Cigarette Use Increases Neutrophil Elastase and Matrix Metalloprotease Levels in the Lung. *American Journal of Respiratory and Critical Care Medicine*, *Journal Article*. https://doi.org/http://dx.doi.org/10.1164/rccm.201903-0615OC

Giovanni, S. P., Keller, T. L., Bryant, A. D., Weiss, N. S., & Littman, A. J. (2020). Electronic cigarette use and chronic respiratory symptoms among U.S. adults. *American Journal of Respiratory and Critical Care Medicine*, *201*(9), 1157–1160. https://doi.org/http://dx.doi.org/10.1164/RCCM.201907-1460LE

Haptonstall, K. P., Choroomi, Y., Moheimani, R., Nguyen, K., Tran, E., Lakhani, K., Ruedisueli, I., Gornbein, J., & Middlekauff, H. R. (2020). Differential effects of tobacco cigarettes and electronic cigarettes on endothelial function in healthy young people. *American Journal of Physiology - Heart and Circulatory Physiology*, *319*(3), H547–H556. https://doi.org/http://dx.doi.org/10.1152/ajpheart.00307.2020

Haziza, C., Bourdonnaye, de La, Donelli, A., Skiada, D., Poux, V., Weitkunat, R., Baker, G., Picavet, P., & Ludicke, F. (2020). Favorable Changes in Biomarkers of Potential Harm to Reduce the Adverse Health Effects of Smoking in Smokers Switching to the Menthol Tobacco Heating System 2.2 for Three Months (Part 2). *Nicotine & Tobacco Research : Official Journal of the Society for Research on Nicotine and Tobacco*, *Journal Article*. https://doi.org/http://dx.doi.org/10.1093/ntr/ntz084

Hedman, L., Backman, H., Stridsman, C., Bosson, J. A., Lundback, M., Lindberg, A., Ronmark, E., & Ekerljung, L. (2018). Association of Electronic Cigarette Use With Smoking Habits, Demographic Factors, and Respiratory Symptoms. *JAMA Network Open*, *1*(3), e180789. https://doi.org/http://dx.doi.org/10.1001/jamanetworkopen.2018.0789

Huilgol, P., Bhatt, S. P., Biligowda, N., Wright, N. C., & Wells, J. M. (2019). Association of e-cigarette use with oral health: a population-based cross-sectional questionnaire study. *Journal of Public Health (Oxford, England)*, *41*(2), 354–361. https://doi.org/http://dx.doi.org/10.1093/pubmed/fdy082

Ibraheem, W. I., Fageeh, H. I., Preethanath, R. S., Alzahrani, F. A., Al-Zawawi, A. S., Divakar, D. D., & Al-Kheraif, A. A. (2020). Comparison of RANKL and osteoprotegerin levels in the gingival crevicular fluid of young cigarette- and waterpipe-smokers and individuals using electronic nicotine delivery systems. *Archives of Oral Biology*, *115*(Journal Article), 104714. https://doi.org/http://dx.doi.org/10.1016/j.archoralbio.2020.104714

Ikonomidis, I., Katogiannis, K., Kostelli, G., Kourea, K., Kyriakou, E., Kypraiou, A., Tsoumani, M., Andreadou, I., Lambadiari, V., Plotas, P., Thymis, I., & Tsantes, A. E. (2020). Effects of electronic cigarette on platelet and vascular function after four months of use. *Food and Chemical Toxicology*, *141*(Journal Article), 111389. https://doi.org/http://dx.doi.org/10.1016/j.fct.2020.111389

Ikonomidis, I., Vlastos, D., Kourea, K., Kostelli, G., Varoudi, M., Pavlidis, G., Efentakis, P., Triantafyllidi, H., Parissis, J., Andreadou, I., Iliodromitis, E., & Lekakis, J. (2018). Electronic cigarette smoking increases arterial stiffness and oxidative stress to a lesser extent than a single conventional cigarette. *Circulation*, *137*(3), 303–306. https://doi.org/http://dx.doi.org/10.1161/CIRCULATIONAHA.117.029153

Ip, M., Diamantakos, E., Haptonstall, K., Choroomi, Y., Moheimani, R. S., Nguyen, K. H., Tran, E., Gornbein, J., & Middlekauff, H. R. (2020). Tobacco and electronic cigarettes adversely impact ECG indexes of ventricular repolarization: implication for sudden death risk. *American Journal of Physiology.Heart and Circulatory Physiology*, *318*(5), H1176–H1184. https://doi.org/http://dx.doi.org/10.1152/ajpheart.00738.2019

Jackson, M., Singh, K. P., Lamb, T., McIntosh, S., & Rahman, I. (2020). Flavor preference and systemic immunoglobulin responses in e-cigarette users and waterpipe and tobacco smokers: A pilot study. *International Journal of Environmental Research and Public Health*, *17*(2), 640. https://doi.org/http://dx.doi.org/10.3390/ijerph17020640

Javed, F., Abduljabbar, T., Vohra, F., Malmstrom, H., Rahman, I., & Romanos, G. E. (2017). Comparison of Periodontal Parameters and Self-Perceived Oral Symptoms Among Cigarette Smokers, Individuals Vaping Electronic Cigarettes, and Never-Smokers. *Journal of Periodontology*, *88*(10), 1059–1065. https://doi.org/http://dx.doi.org/10.1902/jop.2017.170197

Jeong, W., Choi, D. W., Kim, Y. K., Lee, H. J., Lee, S. A., Park, E. C., & Jang, S. I. (2019). Associations of Electronic and Conventional Cigarette Use with Periodontal Disease in South Korean Adults. *Journal of Periodontology*, *Journal Article*. https://doi.org/http://dx.doi.org/10.1002/JPER.19-0060

Karaaslan, F., Dikilitas, A., & Yigit, U. (2020). The effects of vaping electronic cigarettes on periodontitis. *Australian Dental Journal*, *65*(2), 143–149.

https://doi.org/http://dx.doi.org/10.1111/adj.12747

Kelesidis, T., Tran, E., Arastoo, S., Lakhani, K., Heymans, R., Gornbein, J., & Middlekauff, H. R. (2020). Elevated Cellular Oxidative Stress in Circulating Immune Cells in Otherwise Healthy Young People Who Use Electronic Cigarettes in a Cross-Sectional Single-Center Study: Implications for Future Cardiovascular Risk. *Journal of the American Heart Association*, *9*(18), e016983. https://doi.org/http://dx.doi.org/10.1161/JAHA.120.016983

Kim, C., Paek, Y., Seo, H., Cheong, Y., Lee, C., Park, S. M., Park, D., & Lee, K. (2020). Dual use of electronic and conventional cigarettes is associated with higher cardiovascular risk factors in Korean men. *Scientific Reports*, *10*(1 PG-5612), 5612. https://doi.org/http://dx.doi.org/10.1038/s41598-020-62545-3

Kim, T., Choi, H., Kang, J., & Kim, J. (2020). Association between electronic cigarette use and metabolic syndrome in the Korean general population: A nationwide population-based study. *PLoS ONE*, *15*(8), e0237983. https://doi.org/http://dx.doi.org/10.1371/journal.pone.0237983

Kizhakke Puliyakote, A. S., Elliott, A. R., Sa, R. C., Anderson, K. M., Crotty Alexander, L. E., & Hopkins, S. R. (2020). Vaping Disrupts Ventilation-Perfusion Matching in Asymptomatic Users. *Journal of Applied Physiology (Bethesda, Md.: 1985)*, *Journal Article*. https://doi.org/http://dx.doi.org/10.1152/japplphysiol.00709.2020

Leavens, E. L. S., Ford, B. R., Ojo-Fati, O., Winkelman, T. N. A., Vickery, K. D., Japuntich, S. J., & Busch, A. M. (2020). Electronic cigarette use patterns and chronic health conditions among people experiencing homelessness in MN: a statewide survey. *BMC Public Health*, *20*(1). https://doi.org/10.1186/s12889-020-09919-4

Lee, A. C., Chakladar, J., Li, W. T., Chen, C., Chang, E. Y., Wang-Rodriguez, J., & Ongkeko, W. M. (2020). Tobacco, but not nicotine and flavor-less electronic cigarettes, induces ace2 and immune dysregulation. *International Journal of Molecular Sciences*, *21*(15), 1–16. https://doi.org/http://dx.doi.org/10.3390/ijms21155513

Li, D., Sundar, I. K., McIntosh, S., Ossip, D. J., Goniewicz, M. L., O'Connor, R. J., & Rahman, I. (2019). Association of smoking and electronic cigarette use with wheezing and related respiratory symptoms in adults: cross-sectional results from the Population Assessment of Tobacco and Health (PATH) study, wave 2. *Tobacco Control*, *Journal Article*. https://doi.org/http://dx.doi.org/10.1136/tobaccocontrol-2018-054694

Li, D., & Xie, Z. (2020). Cross-Sectional Association of Lifetime Electronic Cigarette Use with Wheezing and Related Respiratory Symptoms in U.S. Adults. *Nicotine & Tobacco Research : Official Journal of the Society for Research on Nicotine and Tobacco*, *22*(1), S85–S92. https://doi.org/http://dx.doi.org/10.1093/ntr/ntaa195

Ludicke, F., Ansari, S. M., Lama, N., Blanc, N., Bosilkovska, M., Donelli, A., Picavet, P., Baker, G., Haziza, C., Peitsch, M., & Weitkunat, R. (2019). Effects of Switching to a Heat-Not-Burn Tobacco Product on Biologically-Relevant Biomarkers to assess a Candidate Modified Risk Tobacco Product: A Randomized Trial. *Cancer Epidemiology, Biomarkers & Prevention : A Publication of the American Association for Cancer Research, Cosponsored by the American Society of Preventive Oncology*, *Journal Article*. https://doi.org/http://dx.doi.org/10.1158/1055-9965.EPI-18-0915

Ludicke, F., Picavet, P., Baker, G., Haziza, C., Poux, V., Lama, N., & Weitkunat, R. (2018). Effects of switching to the menthol tobacco heating system 2.2, smoking abstinence, or continued cigarette smoking on clinically relevant risk markers: A randomized, controlled, open-label, multicenter study in sequential confinement and ambulatory settin. *Nicotine and Tobacco Research*, *20*(2), 173–182. https://doi.org/http://dx.doi.org/10.1093/ntr/ntx028

Mainous, A. G., Yadav, S., Hong, Y. R., & Huo, J. (2020). e-Cigarette and Conventional Tobacco Cigarette Use, Dual Use, and C-Reactive Protein. *Journal of the American College of Cardiology*, *75*(17), 2271–2273. https://doi.org/http://dx.doi.org/10.1016/j.jacc.2020.02.061

Menicagli, R., Marotta, O., & Serra, R. (2020). Free radical production in the smoking of e-cigarettes and their possible effects in human health. *International Journal of Preventive Medicine*, *11*(1), 53. https://doi.org/http://dx.doi.org/10.4103/ijpvm.IJPVM_424_19

Meo, S. A., Ansary, M. A., Barayan, F. R., Almusallam, A. S., Almehaid, A. M., Alarifi, N. S., Alsohaibani, T. A., & Zia, I. (2019). Electronic Cigarettes: Impact on Lung Function and Fractional Exhaled Nitric Oxide Among Healthy Adults. *American Journal of Men's Health*, *13*(1), 1557988318806073. https://doi.org/http://dx.doi.org/10.1177/1557988318806073

Mokeem, S. A., Abduljabbar, T., Al-Kheraif, A. A., Alasqah, M. N., Michelogiannakis, D., Samaranayake, L. P., & Javed, F. (2019). Oral Candida carriage among cigarette- and

waterpipe-smokers, and electronic cigarette users. *Oral Diseases*, *25*(1), 319–326. https://doi.org/http://dx.doi.org/10.1111/odi.12902

Mokeem, S. A., Alasqah, M. N., Michelogiannakis, D., Al-Kheraif, A. A., Romanos, G. E., & Javed, F. (2018). Clinical and radiographic periodontal status and whole salivary cotinine, IL-1beta and IL-6 levels in cigarette- and waterpipe-smokers and E-cig users. *Environmental Toxicology and Pharmacology*, *61*(Journal Article), 38–43. https://doi.org/http://dx.doi.org/10.1016/j.etap.2018.05.016

Moon, J., Lee, H., Kong, M., Kim, H., & Oh, Y. (2020). Association Between Electronic Cigarette Use and Levels of High-Sensitivity C-Reactive Protein and Uric Acid. *Asia-Pacific Journal of Public Health*, *32*(1), 35–41. https://doi.org/http://dx.doi.org/10.1177/1010539519899777

Oliveri, D., Liang, Q., & Sarkar, M. (2020). Real-World Evidence of Differences in Biomarkers of Exposure to Select Harmful and Potentially Harmful Constituents and Biomarkers of Potential Harm Between Adult E-Vapor Users and Adult Cigarette Smokers. *Nicotine & Tobacco Research : Official Journal of the Society for Research on Nicotine and Tobacco*, *22*(7 PG-1114–1122), 1114–1122. https://doi.org/https://dx.doi.org/10.1093/ntr/ntz185

Osei, A. D., Mirbolouk, M., Orimoloye, O. A., Dzaye, O., Uddin, S. M. I., Benjamin, E. J., Hall, M. E., DeFilippis, A. P., Stokes, A., Bhatnagar, A., Nasir, K., & Blaha, M. J. (2019). Association Between E-Cigarette Use and Cardiovascular Disease Among Never and Current Combustible-Cigarette Smokers. *American Journal of Medicine*, *132*(8), 949. https://doi.org/http://dx.doi.org/10.1016/j.amjmed.2019.02.016

Parekh, T., Pemmasani, S., & Desai, R. (2020). Risk of stroke with e-cigarette and combustible cigarette use in young adults. *American Journal of Preventive Medicine*, *58*(3 PG-446–452), 446–452. http://ovidsp.ovid.com/ovidweb.cgi?T=JS&CSC=Y&NEWS=N&PAGE=fulltext&D=psyc17&AN=2020-14949-017

Perez, M. F., Atuegwu, N. C., Mortensen, E. M., & Oncken, C. (2020). The inflammatory biomarker YKL-40 is elevated in the serum, but not the sputum, of E-cigarette users. *Experimental Lung Research*, *Journal Article*. https://doi.org/http://dx.doi.org/10.1080/01902148.2020.1847216

Podzolkov, V. I., Bragina, A. E., Druzhinina, N. A., Vasil'eva, L. V, Osadchiy, K. K., Dubchak, A. E., & Khvalin, E. I. (2020). Relation between Tobacco Smoking/Electronic Smoking and Albuminuria/Vascular Stiffness in Young People without Cardiovascular Diseases. *Kidney and Blood Pressure Research*, *45*(3), 467–476. https://doi.org/http://dx.doi.org/10.1159/000507510

Polosa, R., Cibella, F., Caponnetto, P., Maglia, M., Prosperini, U., Russo, C., & Tashkin, D. (2017). Health impact of E-cigarettes: a prospective 3.5-year study of regular daily users who have never smoked. *Scientific Reports*, *7*(1), 13825. https://doi.org/http://dx.doi.org/10.1038/s41598-017-14043-2

Polosa, R., Morjaria, J. B., Prosperini, U., Busa, B., Pennisi, A., Malerba, M., Maglia, M., & Caponnetto, P. (2020). COPD smokers who switched to e-cigarettes: health outcomes at 5-year follow up. *Therapeutic Advances in Chronic Disease*, *11*(Journal Article). https://doi.org/http://dx.doi.org/10.1177/2040622320961617

Polosa, R., Morjaria, J. B., Prosperini, U., Russo, C., Pennisi, A., Puleo, R., Caruso, M., & Caponnetto, P. (2018). Health effects in COPD smokers who switch to electronic cigarettes: A retrospective-prospective 3-year follow-up. *International Journal of COPD*, *13*(Journal Article), 2533–2542. https://doi.org/http://dx.doi.org/10.2147/COPD.S161138

Pulvers, K., Nollen, N. L., Rice, M., Schmid, C. H., Qu, K., Benowitz, N. L., & Ahluwalia, J. S. (2020). Effect of Pod e-Cigarettes vs Cigarettes on Carcinogen Exposure among African American and Latinx Smokers: A Randomized Clinical Trial. *JAMA Network Open*, *3*(11), 26324. https://doi.org/http://dx.doi.org/10.1001/jamanetworkopen.2020.26324

Pushalkar, S., Paul, B., Li, Q., Yang, J., Vasconcelos, R., Makwana, S., Gonzalez, J. M., Shah, S., Xie, C., Janal, M. N., Queiroz, E., Bederoff, M., Leinwand, J., Solarewicz, J., Xu, F., Aboseria, E., Guo, Y., Aguallo, D., Gomez, C., … Saxena, D. (2020). Electronic Cigarette Aerosol Modulates the Oral Microbiome and Increases Risk of Infection. *IScience*, *23*(3 PG-100884), 100884. https://doi.org/https://dx.doi.org/10.1016/j.isci.2020.100884

Rebuli, M. E., Glista-Baker, E., Hoffman, J. R., Duffney, P. F., Robinette, C., Speen, A. M., Pawlak, E. A., Dhingra, R., Noah, T. L., & Jaspers, I. (2021). Electronic-cigarette use alters nasal mucosal immune response to live-attenuated influenza virus: A clinical trial. *American Journal of Respiratory Cell and Molecular Biology*, *64*(1), 126–137. https://doi.org/http://dx.doi.org/10.1165/rcmb.2020-0164OC

Reidel, B., Radicioni, G., Clapp, P. W., Ford, A. A., Abdelwahab, S., Rebuli, M. E., Haridass, P., Alexis, N. E., Jaspers, I., & Kesimer, M. (2018). E-cigarette use causes a unique innate immune response in the lung, involving increased neutrophilic activation and altered mucin secretion. *American Journal of Respiratory and Critical Care Medicine*, *197*(4), 492–501. https://doi.org/http://dx.doi.org/10.1164/rccm.201708-1590OC

Rodu, B., & Plurphanswat, N. (2020). A re-analysis of e-cigarette use and heart attacks in PATH wave 1 data. *Addiction (Abingdon, England)*, *115*(11), 2176–2179. https://doi.org/http://dx.doi.org/10.1111/add.15067

Sakaguchi, C., Nagata, Y., Kikuchi, A., Takeshige, Y., & Minami, N. (2021). Differences in Levels of Biomarkers of Potential Harm among Users of a Heat-not-burn Tobacco Product, Cigarette Smokers, and Never-Smokers in Japan: A Post-Marketing Observational Study. *Nicotine & Tobacco Research : Official Journal of the Society for Research on Nicotine and Tobacco*, *Journal Article PG-*. https://doi.org/https://dx.doi.org/10.1093/ntr/ntab014

Sakamaki-Ching, S., Williams, M., Hua, M., Li, J., Bates, S. M., Robinson, A. N., Lyons, T. W., Goniewicz, M. L., & Talbot, P. (2020). Correlation between biomarkers of exposure, effect and potential harm in the urine of electronic cigarette users. *BMJ Open Respiratory Research*, *7*(1), e000452. https://doi.org/http://dx.doi.org/10.1136/bmjresp-2019-000452

Schneller, L. M., Quinones Tavarez, Z., Goniewicz, M. L., Xie, Z., McIntosh, S., Rahman, I., O'Connor, R. J., Ossip, D. J., & Li, D. (2020). Cross-Sectional Association Between Exclusive and Concurrent Use of Cigarettes, ENDS, and Cigars, the Three Most Popular Tobacco Products, and Wheezing Symptoms Among U.S. Adults. *Nicotine & Tobacco Research : Official Journal of the Society for Research on Nicotine and Tobacco*, *22*(1), S76–S84. https://doi.org/http://dx.doi.org/10.1093/ntr/ntaa199

Shields, P. G., Song, M. A., Freudenheim, J. L., Brasky, T. M., McElroy, J. P., Reisinger, S. A., Weng, D. Y., Ren, R., Eissenberg, T., Wewers, M. D., & Shilo, K. (2020). Lipid laden macrophages and electronic cigarettes in healthy adults. *EBioMedicine*, *60*(Journal Article), 102982. https://doi.org/http://dx.doi.org/10.1016/j.ebiom.2020.102982

Singh, K. P., Lawyer, G., Muthumalage, T., Maremanda, K. P., Khan, N. A., McDonough, S. R., Ye, D., McIntosh, S., & Rahman, I. (2019). Systemic biomarkers in electronic cigarette users: Implications for noninvasive assessment of vaping-associated pulmonary injuries. *ERJ Open Research*, *5*(4), 182–2019. https://doi.org/http://dx.doi.org/10.1183/23120541.00182-2019

Sinha, D. K., Vishal, Kumar, A., Khan, M., Kumari, R., & Kesari, M. (2021). Evaluation of tumor necrosis factor-alpha (TNF-alpha) and interleukin (IL)-1beta levels among subjects vaping e-cigarettes and nonsmokers. *Journal of Family Medicine and Primary Care*, *9*(2 PG-1072–1075), 1072–1075. https://doi.org/https://dx.doi.org/10.4103/jfmpc.jfmpc_902_19

Song, M. A., Freudenheim, J. L., Brasky, T. M., Mathe, E. A., McElroy, J. P., Nickerson, Q. A., Reisinger, S. A., Smiraglia, D. J., Weng, D. Y., Ying, K. L., Wewers, M. D., & Shields, P. G. (2020). Biomarkers of exposure and effect in the lungs of smokers, nonsmokers, and electronic cigarette users A C. *Cancer Epidemiology Biomarkers and Prevention*, *29*(2), 443–451. https://doi.org/http://dx.doi.org/10.1158/1055-9965.EPI-19-1245

Song, M. A., Reisinger, S. A., Freudenheim, J. L., Brasky, T. M., Mathe, E. A., McElroy, J. P., Nickerson, Q. A., Weng, D. Y., Wewers, M. D., & Shields, P. G. (2020). Effects of electronic cigarette constituents on the human lung: A pilot clinical trial. *Cancer Prevention Research*, *13*(2), 145–151. https://doi.org/http://dx.doi.org/10.1158/1940-6207.CAPR-19-0400

Stokes, A. C., Xie, W., Wilson, A. E., Yang, H., Orimoloye, O. A., Harlow, A. F., Fetterman, J. L., DeFilippis, A. P., Benjamin, E. J., Robertson, R. M., Bhatnagar, A., Hamburg, N. M., & Blaha, M. J. (2021). Association of Cigarette and Electronic Cigarette Use Patterns With Levels of Inflammatory and Oxidative Stress Biomarkers Among US Adults: Population Assessment of Tobacco and Health Study. *Circulation*, *Journal Article*. https://doi.org/http://dx.doi.org/10.1161/CIRCULATIONAHA.120.051551

Tackett, A. P., Keller-Hamilton, B., Smith, C. E., Hebert, E. T., Metcalf, J. P., Queimado, L., Stevens, E. M., Wallace, S. W., McQuaid, E. L., & Wagener, T. L. (2020). Evaluation of Respiratory Symptoms among Youth e-Cigarette Users. *JAMA Network Open*, *Journal Article*, e2020671. https://doi.org/http://dx.doi.org/10.1001/jamanetworkopen.2020.20671

Vindhyal, M. R., Okut, H., Ablah, E., Ndunda, P. M., Kallail, K. J., & Choi, W. S. (2020). Cardiovascular Outcomes Associated With Adult Electronic Cigarette Use. *Cureus*, *12*(8 PG-e9618), e9618. https://doi.org/https://dx.doi.org/10.7759/cureus.9618

Vohra, F., Bukhari, I. A., Sheikh, S. A., Albaijan, R., & Naseem, M. (2020). Comparison of self-rated oral symptoms and periodontal status among cigarette smokers and individuals using electronic nicotine delivery systems. *Journal of American College Health : J of ACH*, *68*(7), 788–793. https://doi.org/http://dx.doi.org/10.1080/07448481.2019.1709476

Vora, M. V, & Chaffee, B. W. (2019). Tobacco-use patterns and self-reported oral health outcomes: A cross-sectional assessment of the Population Assessment of Tobacco and Health study, 2013-2014. *Journal of the American Dental Association (1939)*, *150*(5), 332–344. https://doi.org/http://dx.doi.org/10.1016/j.adaj.2018.12.004

Wang, J. B., Olgin, J. E., Nah, G., Vittinghoff, E., Cataldo, J. K., Pletcher, M. J., & Marcus, G. M. (2018). Cigarette and e-cigarette dual use and risk of cardiopulmonary symptoms in the Health eHeart Study. *PLoS ONE*, *13*(7), e0198681. https://doi.org/http://dx.doi.org/10.1371/journal.pone.0198681

Xie, W., Kathuria, H., Galiatsatos, P., Blaha, M. J., Hamburg, N. M., Robertson, R. M., Bhatnagar, A., Benjamin, E. J., & Stokes, A. C. (2020). Association of Electronic Cigarette Use with Incident Respiratory Conditions among US Adults from 2013 to 2018. *JAMA Network Open*, *3*(11), e2020816. https://doi.org/http://dx.doi.org/10.1001/jamanetworkopen.2020.20816

Ye, D., Gajendra, S., Lawyer, G., Jadeja, N., Pishey, D., Pathagunti, S., Lyons, J., Veazie, P., Watson, G., McIntosh, S., & Rahman, I. (2020). Inflammatory biomarkers and growth factors in saliva and gingival crevicular fluid of e-cigarette users, cigarette smokers, and dual smokers: A pilot study. *Journal of Periodontology*, *91*(10), 1274–1283. https://doi.org/http://dx.doi.org/10.1002/JPER.19-0457
