# Supplementary material for: Chronic health effects associated with electronic cigarette use: A systematic review
Source: Front Public Health. 2022 Oct 6;10:959622. doi: 10.3389/fpubh.2022.959622 (PMC9584749; doi:10.3389/fpubh.2022.959622)
Supplement: Supplementary file 3 [file Table_3.pdf]

Supplement S3: Study findings

Color code and definitions of e-cigarette users (ECU) subgroups based on exposure:

ECU sub-group/s	Study final ECU exposure category	Exclusive ECU status	
		NO	YES
If study includes:<br>• "Daily users"<br>• "Daily AND occasional users" each in a separate group.	Daily e-cigarette use (ECU)	ECU could be former smokers or smoking status was unclear/ unspecified in the study	ECU were never smokers
If study includes:<br>• "Occasional users"<br>• "Daily OR occasional users" combined together in one group.	Occasional e-cigarette use (ECU)	ECU could be former smokers or it was unclear/ unspecified in the study	ECU were never smokers
If study includes:<br>• Exposure is not specified /unclear.<br>• Ever users.<br>• Combined daily or occasional users with an unclear exposure.	Unclear e-cigarette use (ECU)	ECU could be former smokers or it was unclear/ unspecified in the study	ECU were never smokers

Table S3.1: Cardiovascular health

First author (year)	Findings
**Cardiovascular health (n=26)**	
**Randomized controlled trials (n=6)**	
(George et al. 2019)	• Compared to TS, ECU did not display a significant difference in systolic blood pressure (Difference: -2.16 (-4.79-0.47); p=0.10), diastolic blood pressure (Difference: -1.13 (-2.62-0.37); p=0.14), heart rate (Difference: -1.19 (-3.05-0.67); p=0.21), augmentation index score (Difference: 0.11 (-1.83-2.06); p=0.91), pulse wave velocity (difference: 0.167 (0.402 to 0.069); p-value= 0.164) at 1 month follow-up.<br>• Brachial artery flow-mediated dilation (FMD) was assessed in male and female participants separately. Among male participants, ECU and TS did not display a significant difference in brachial artery FMD (nicotine free = 0.448 (0.451 to 1.347), p= 0.315; nicotine = 0.822 (0.067 to 1.710), p= 0.069). Among female participants, ECU displayed significantly improved brachial artery FMD (nicotine free = 2.183 (1.336 to 3.030) <0.0001; nicotine = 1.824 (0.942 to 2.706) <0.0001).

First author (year)	Findings
(Haziza et al. 2020)	• Compared to TS, ECU did not display a significant difference in systolic blood pressure (LS mean difference: -0.7 (-4.5-3.1); p=0.71), diastolic blood pressure (LS mean difference: 0.2 (-3.7-4.0); p=0.93), total cholesterol levels (LS mean difference: -0.4 (-13.3-5.2); p=0.39), HCL-c (LS mean difference: 1.4 (-2.3-5.0); p=0.45), LDL-c (LS mean difference: -3.3 (-12.0-5.4); p=0.45), or triglyceride levels (LS mean difference: 0.9 (-12.8-14.6); p=0.90) at 90-days follow-up.<br>• Compared to NS, ECU did not display a significant difference in systolic blood pressure (LS mean difference: 1.7 (−4.3; 7.7); p=0.58), diastolic blood pressure (LS mean difference: 2.4 (−3.7; 8.4); p=0.43]), total cholesterol levels (LS mean difference: −1.4 (−16.1; 13.2); p=0.85), HCL-c (LS mean difference: 1.3 (−4.4; 7.1); p=0.65), LDL-c (LS mean difference -5.1 (-18.9; 8.6); p=0.46), or triglyceride levels (LS mean difference: 11.6 (−10.1; 33.4); p=0.29) at 90-days follow-up.
(Ikonomidis et al. 2020)[1]	• Compared to TS, ECU were found to have significantly decreased pulse wave velocity levels (p=0.047), but not systolic (p=0.97) or diastolic (p=0.64) blood pressure at 4-months follow-up.
(Ludicke et al. 2018)[2]	• At 90-day follow up, there was no significant difference in levels of LDL cholesterol (p=0.2290), HDL cholesterol (p=0.2944), total cholesterol (p=0.0719), systolic blood pressure (p=0.6607) or diastolic blood pressure (p=0.1800) between ECU and NS. The level of triglycerides was significantly lower in ECU users than NS at 90-day follow up (p=0.0199).<br>• At 90-day follow up, there was no significant difference in levels of LDL cholesterol (p=0.8162), triglycerides (p=0.4095), total cholesterol (p=0.6499), systolic blood pressure (p=0.7157) or diastolic blood pressure (p=0.5705) between ECU users and TS. The level of HDL cholesterol was significantly higher in ECU than TS smokers at 90-day follow up (p=0.0084).
(Ludicke et al. 2019)[3]	• Compared to TS, ECU demonstrated a significantly higher HCL-c level at 6-month follow-up (LS mean difference: 3.09; 95%CI: 1.10-5.09 p<0.001).
(Pulvers et al. 2020)[4]	• Compared to TS, ECU did not demonstrate a significant difference in systolic (RR: 1.01 (0.93-1.09)) or diastolic (RR: 0.99 (0.92-1.06)) blood-pressure at 6-weeks follow-up.<br>• Compared to DU, ECU did not demonstrate a significant difference in systolic (RR: 1.01 (0.96-1.07) or diastolic (RR: 1.00 (0.96-1.05) blood-pressure at 6-weeks follow-up.
**Pre-post (n=1)**	
(Ikonomidis et al. 2018)[5]	• Compared to TS, ECU had a significant decrease in systolic blood pressure (p=0.03) and augmentation index score (p=0.001) at 1-month follow-up, but no significant differences in diastolic blood pressure (p=0.2), heart rate (p=0.4), or peak wave velocity (p=0.4)
**Cross-sectional (n=19)**	
(Aherrera et al. 2020)[6]	• Compared to NS, ECU did not differ in the reporting of hypertension, after controlling for age and sex, and previous cigarette smoking status (p>0.05)
(Arastoo et al. 2020)	• Compared to TS, ECU were not found to have significantly different systolic blood pressure (p=0.62), diastolic blood pressure (p=0.25), mean blood pressure (p=0.26), and heart rate (p=0.37)
(Alzahrani et al. 2018)	• Compared to NS, regular ECU were found to have a significant increase in odds of myocardial infarction (OR: 1.79 (1.20-2.66); p=0.04), but not occasional ECU (OR:1.16 (0.83-1.62); p=0.392)

[1] Ikonomidis et al. (2020): Also check immunological health
[2] Ludicke et al. (2018): Also check immunological and respiratory health
[3] Ludicke et al. (2019): Also check respiratory health
[4] Pulvers et al. (2020) : Also check respiratory health
[5] Ikonomidis et al. (2018): Also check immunological health
[6] Aherrera et al. (2020): Also check immunological health

First author (year)	Findings
(Badea et al. 2019)[7]	• Compared to TS, ECU did not display significantly different levels of total cholesterol, HDL-c, LDL-c, and triglycerides (p>0.05).<br><br>• Compared to NS, ECU did not display significantly different levels of total cholesterol, HDL-c, and triglycerides, (p>0.05), but did display significantly higher levels of LDL-c (p<0.05).
(Farsalinos et al. 2019)	• Compared to NS, ECU were not found to have significantly different odds of having experienced a myocardial infarction (daily aOR 1.35 [0.80–2.27]; p=0.267// occasional (OR 1.22 (0.78–1.91); p= 0.373), coronary heart disease (daily OR 1.31 (0.79–2.17); p=0.286 // occasionally OR 1.13 (0.70–1.83); p=0.624).
(Fetterman et al. 2020)	• Diastolic blood pressure (p=0.14), heart rate (p=0.1), brachial-artery diameter (p=0.63), brachial-artery flow mediated dilation (p=0.68), carotid-femoral flow mediated dilation (p=0.12), carotid-radial flow mediated dilation (p=0.2), shear stress reactive hyperemia (p=0.73), baseline mean flow velocity (p=0.1), and hyperemic mean flow velocity (p=0.79) did not differ significantly across all groups (e.g., ECU, NS, TS and DU).<br>• Systolic blood pressure (p=0.007) and augmentation index (p=0.0008) differ significantly across groups.<br>• Augmentation index did not differ significantly (p>0.05) between other comparisons (ECU vs NS, ECU vs TS, and ECU vs DU).Systolic blood pressure did not differ significantly (p>0.05) between comparisons (ECU vs NS, ECU vs TS, and ECU vs DU).
(Haptonstall et al. 2020)	• Compared to NS, ECU did not display significantly different results for baseline systolic (p=0.29), or diastolic blood pressure (p=0.5), mean blood pressure (p=0.5), heart rate (p=0.1), shear stress reactive hyperemia (p=0.5), velocity reactive hyperemia (p=0.5), peak shear rate (p>0.05), and flow-mediated dilation (p>0.05).<br>• Compared to TS, ECU did not display significantly different results for baseline systolic (p=0.2), or diastolic blood pressure (p=0.2), mean blood pressure (p=0.2), heart rate (p=0.9), shear stress reactive hyperemia (p=0.4), velocity reactive hyperemia (p=0.5), peak shear rate (p>0.05), and flow-mediated dilation (p>0.05).<br>• Artery diameter significantly differed across three groups (p=0.008). When post-hoc test was done, ECU had significantly higher artery diameter than NS (p<0.05) and did not differ significantly from TS (p>0.05).
(Ip et al. 2020)	• Compared to both NS and TS, ECU did not have significantly higher heart-rates (bpm) at baseline (p=0.48).
(T. Kim et al. 2020)	• Compared to NS, ECU did not have a significant difference in odds of having low HDL-cholesterol (p=0.16) or hypertension (p=0.60).<br>• Compared to NS, ECU had significantly higher odds of having high triglycerides (p= 0.01).
(C. Kim et al. 2020)	• Compared to non-smokers, dual users saw a significantly decreased risk of high blood-pressure (aPOR:0.62 (0.41-0.94); p=0.023), and a significantly increased risk of elevated tryglycerides (aPOR: 2.81 (1.90-4.14); p<0.001) and low HDL-c (aPOR: 2.48 (1.66-3.71); p<0.001), but no significant differences in the prevalence of comorbidities such as hyperlipidemia or cardiovascular disease (p>0.05)
(Leavens et al. 2020)[8]	• Compared to NS, TS, and DU, ECU did not report significantly differential rates of hypertension (p>0.050).
(Oliveri, Liang, and Sarkar 2020)[9]	• Compared to TS, ECU did not demonstrate significantly different levels of HDL-c levels (LS mean diff: 1.4 (-3.1-5.9); p=0.54)

[7] Badea et al. (2019): Also check immunological health
[8] Leavens et al. (2020) : Also check respiratory health
[9] Oliveri et al. (2020) : Also check immunological health

First author (year)	Findings
(Osei et al. 2019)	• Compared to NS, regular or occasional ECU did not exhibit significantly different odds of cardiovascular disease (OR:1.35 (0.74-2.46); 0.95 (0.50-1.82)) or premature cardiovascular disease (under the age of 65) (1.16 (0.56-2.41); 0.97 (0.47-2.02))
(Parekh, Pemmasani, and Desai 2020)	• Compared to NS, ECU did not demonstrate significantly different odds of ischemic attack (OR: 0.69 (0.34-1.42))<br>• Compared to TS, ECU demonstrated significantly reduced odds of ischemic attack (OR: 0.43 (0.20-0.93))
(Podzolkov et al. 2020)	• Compared to TS, daily ECU were found to have significantly higher albuminuria levels (160 mg/L [150; 207.5]) vs (115 mg/L [60; 200]) (p<0.05), but no significant difference in augmentation index levels (–5.05 [–13.4; –3.3]) vs (–4 [–6.6; –1.9]) (p>0.05).<br>• Compared to NS, daily ECU were found to have significantly higher albuminuria (160 mg/L [150; 207.5]) vs (20 mg/L [10; 50]) and augmentation index levels (–5.05[–13.4; –3.3]) vs s (–16.2 [–23.9; –7]) (p<0.05), but no significant difference in systolic or diastolic blood pressure, high blood pressure (p>0.05).<br>• High albuminuria and augmentation index are non invasive markers for arterial stiffness.
(Rodu and Plurphanswat 2020)	• Compared to NS, daily ECU did not demonstrate significantly different odds of myocardial infarction (OR: 0.69 (0.22-2.12)), however occasional ECU demonstrated significantly decreased odds (OR: 0.18 (0.05-0.66))
(Sakaguchi et al. 2021)[10]	• Compared to TS, ECU demonstrated a significant increase in HCL-c levels (p<0.0001) and a significant decrease in triglycerides (p=0.0004), but not total cholesterol levels (p=0.66) or LDL-c (p=0.90)<br>• Compared to NS, ECU were not found to demonstrate significant differences in HDL-c (p=0.73), LDL-c (p=0.31), total cholesterol (p=0.16), or triglyceride levels (p=0.62)
(Vindhyal et al. 2020)	• Compared to NS, daily ECU demonstrated significantly increased odds of myocardial infarction (OR: 4.09 (1.29-12.98)), but not ischemic attack (OR: 1.22 (0.36-4.18)) or coronary heart disease (OR: 0.67 (0.18-2.44).
(Wang et al. 2018)[11]	• Compared to NS, more ECU reported arrhythmia 13.8% vs 15.1% p=0.03 when adjusting for congestive heart failure and coronary artery disease. Moreover, ECU were more likely to report coronary artery disease 10.9% vs 9.9% compared to NS. No significant difference in, high blood pressure, high cholesterol, congestive heart failure, myocardial infraction, or ischemic attack.

Table S3.2: Immunological health

First author (year)	Findings
**Immune health (n=45)**	
**Randomized controlled trials (n=7)**	
(George et al. 2019)[12]	• Compared to TS and dual-users, ECU were not found to have any significant differences in the levels of hs-CRP (p=0.8), tissue plasminogen activator (p=0.4), and platelet activation inhibitor-1 (p=0.9).

[10] Sakaguchi et al. (2021) : Also check immunological and respiratory health

[11] Wang et al. (2020): Also check respiratory health.

[12] George et al. (2019): All immunological health indicators were measured as indicator to cardiovascular health. Also see in cardiovascular health.

First author (year)	Findings	
(Haziza et al. 2020)[13]	• Oxidative stress: At 90-day follow-up, ECU users showed significantly lower levels of 8-epi-PGF2a than TS (p=0.0237), but was not significantly different from NS (p=0.5744).<br>• Platelet Activation: At 90-day follow up, there was no significant difference in levels of 11-DTX-B2 between ECU and TS (p=0.7701) or abstinent participants. (p=0.8461).<br>• At 90-day follow up, ECU had significantly higher levels of WBC counts than non-users (p=0.0364). WBC counts did not significantly differ between ECU and TS (p=0.5954).<br>• Inflammation: At 90-day follow up, there was no significant difference in levels of hs-CRP or fibrinogen between ECU and TS (p=0.3482 and 0.6783, respectively) or abstinent participants (p=0.9296 and 0.3482, respectively).<br>• ECU had significantly lower levels of sICAM-1 compared to cigarettes smokers (p=0.0023) but did not differ significantly from abstinent participants (p=0.8929).	
(Ikonomidis et al. 2020)[14]	• Compared to TS, ECU were found to have significantly decreased Malondialdehyde levels (1.39 (0.1) vs 1.09 (0.10)) at 4-months follow-up (p=0.03).	
(Ludicke et al. 2018)[15]	• Oxidative Stress: At 90-day follow up, ECU users showed significantly lower levels of 8-epi-PGF2a than TS smokers (p=0.0159), but was not significantly different from NS (p=0.2).<br>• Platelet Activation: At 90-day follow up, there was no significant difference in levels of 11-DTX-B2 between ECU and TS (p=0.1) or abstinent participants (NS) (p=0.06).<br>• Inflammation: At 90-day follow up, there was no significant difference in levels of hs-CRP or fibrinogen between ECU and TS or abstinent participants (NS) (p>0.05).<br>• At 90-day follow up, there was a significantly lower level of white blood cells between ECU and TS (p=0.02) but not abstinent participants (p=0.5).	
(Ludicke et al. 2019)[16]	• Compared to TS who continued smoking at 6 months, ECU had significantly lower white blood cell levels (p= 0.001), and favourable 8-epi-PGF2a (p=0.02) and sICAM-1 (p=0.03), but not significant 11-DTX-B2 (p=0.2).	
(Song, Reisinger, et al. 2020)	• There was no significant difference between NS and ECU in changes in immune cells (macrophages, lymphocytes, neutrophils, and eosinophils) or cytokines (INF-γ, IL-1β, IL-2, IL-4, IL-6, IL-8, IL-10, IL-12p70, IL-13, and TNF-α) between baseline and follow up (p>0.05).	
(Rebuli et al. 2021)	• Compared to NS, ECU had significantly decreased levels in immune response indicators (IgA) and inflammation biomarkers (MIP-1α, Il-6, Il-12P40 and INF-γ) .<br>• Compared to NS, ECU had significantly increased levels in inflammation biomarkers, (IL-1α, IL-2, and VEGF) but no difference in MCP-1 and MIP-1β.	
**Pre-post studies (n=1)**		
(Ikonomidis et al. 2018)[17]	• Compared to TS, ECU did not result in any significant changes in platelet function assay results (p=0.454) at 1-month follow-up.<br>• Compared to TS, ECU resulted in a significant reduction in MDA (oxidative stress) (p=0.03) at 1-month follow-up.	
**Case-control studies** (n=2)		

[13] Haziza et al. (2020): All immunological health outcomes were measured in context of cardiovascular health. Also see cardiovascular health

[14] Ikonomidis et al. (2020): Also check cardiovascular health

[15] Ludicke et al. (2018): All immunological health outcomes were measured in context of cardiovascular health. Also see in respiratory, and cardiovascular health.

[16] Ludickeet al. (2019): All immunological health outcomes were measured in context of cardiovascular health. Also check in respiratory health

[17] Ikonomidis et al. (2020): Also check cardiovascular health

First author (year)	Findings
(Ibraheem et al. 2020)[18]	• Compared to NS (0.21 ± 0.007 µl), the GCF volume was significantly higher among ECU (ENDS-users) (0.62 ± 0.03 µl) (P < 0.01) than non-smokers (0.21 ± 0.007 µl).
(Karaaslan, Dikilitas, and Yigit 2020)[19]	• ECU had significantly higher GCF volume, IL-8, and TNF-α than NS (p=0.001).<br>• ECU had significantly lower GCF volume, IL-8, and TNF-α than TS (p=0.001).<br>• Compared to TS, ECU did not have a statistically different mean GsH-Px level (P > 0.05).<br>• Compared to NS (former smokers), ECU had significantly higher mean GsH-Px levels (p=0.001).<br>• Compared to both TS and NS (former smokers), there was no significant difference among the three groups for mean 8-OHdG level (one-way ANOVA test, P > 0.05).
**Cross-sectional studies** (n=35)	
(Al Deeb et al. 2020)[20]	• Compared to NS, at baseline ECU had significantly higher levels of MMP-8 (p<0.0001) and TNF-α (p<0.0001).<br>• Compared to TS, at baseline ECU had significantly lower levels of MMP-8 (p<0.002) and TNF-α (p<0.0001).
(Al-Aali et al. 2018)	• Compared to NS, ECU produced significantly more PISF volume on average (1.5 [0.5] vs 3.1 [0.6]; p<0.01).<br>• Compared to NS, ECU had significantly higher average concentrations of TNF-α (6.7 [8.1] vs 24.3 [32.4]; p<0.001) and IL-1β (19.7 [22.3] vs 205.2 [230.7]; p<0.01]).
(Al-Hamoudi et al. 2020)	• Compared to NS, ECU did not display any differences in GCF volume at baseline (1.65 [0.37] vs 1.74 [0.41]; p>0.05).<br>• Compared to NS, ECU did not display any significant difference on IL-4 (pg/µL) (0.15 [0.13] vs 0.16 [0.12]), IL-9 (pg/µL) (0.5 [0.12] vs 0.33 [0.2]), IL-10 (pg/µL) (1.34 [0.15] vs 1.11 [0.08]), nor IL-13 (pg/µL) (0.85 [0.16] vs. 0.74 [0.12]) at baseline.
(AlQahtani et al. 2018)[21]	• Compared to NS, ECU had significantly higher mean volumes of PISF (1.5 [0.5] vs 3.2 [0.5]; p<0.05) (as did TS).<br>• Compared to NS, ECU had significantly higher mean concentrations of TNF-α (24.3 [32.4] vs 6.7 [8.1]), IL-6 (1792.8 [94.1] vs 1259.7 [91.4]), and IL-1β (205.2 [230.7] vs 19.7 [22.3]).
(F. Alqahtani et al. 2019)	• Compared to NS, ECU had significantly higher mean levels of PISF volume (0.16 [0.03] vs 0.36 [0.05]; p<0.05) (as did cigarette and water-pipe smokers).
(S. Alqahtani et al. 2020)[22]	• Compared to NS, ECU did not display a significantly different saliva levels of IL-6 or IL-8.<br>• Compared to NS, ECU had elevated saliva levels of IL-1β, TNF-α, prostaglandins, leukotrienes, glutathionyl spermine (3 folds); gangliosides (2 folds or more); Sulfogalactosylceramide (3 folds); angiotensin II (1.71 fold); phosphotrosyl-angiotensin II (2.98 fold); and other metabolies such as sphingolipids, glycerophospholipids, ceramides, and pentosidine (p<0.05).
(ArRejaie 2019)[23]	• Compared to NS, ECU had a significantly higher mean volume of PISF (1.52 [0.5] vs 3.16 [0.6]; p<0.01).<br>• Compared to TS, ECU did not have a significantly different mean volume of PISF (3.31 [0.4] vs 3.16 [0.6]; p>0.05).<br>• Compared to NS, mean levels of IL-1β were higher in ECU, (36.91 (22.3) vs 196.23 (90.8)); as were mean levels of MMP-9 [27.72 (18.3) vs 88.34 (32.4)].

[18] (Ibraheem et al. 2020): Also check oral health
[19] Karaaslan et al.(2020): Also check oral health
[20] Al Deeb et al. (2020): All immunological health outcomes were collected from peri-implant sulcular fluid (PISF) for periodontal outcomes. Also check oral health
[21] AlQahtani et al. (2018): Immunological indicators were measured as indicators of oral health. Also check oral health
[22] AlQahtani et al. (2020): Immunological indicators were measured as indicators of oral health. Also check oral health
[23] ArRejaie et al. (2019): Immunological indicators were measured as indicators of peri-implants clinical assessments. Also check oral health.

First author (year)	Findings
	• The mean levels of IL-1β in TS (246.53 (115.2) (P<0.001) as well as mean levels of MMP-9 in TS [113.59 (29.6)] were higher than those observed in ECU.
(Ashford et al. 2020)[24]	• Compared to NS, ECU did not display significantly different levels of IL-6 (p=0.56), IL-8 (p=0.47), IL-10 (p=0.9), IL12p70 (p=0.078), IL-13 (p=0.75), and TNF-α (p=0.14).<br>• Compared to NS, ECU displayed significantly elevated levels of IL-2 (p=0.023) and INF-γ (p=0.008), but significantly depressed levels of IL-4 (p=0.034).
(Badea et al. 2019)[25]	• Compared to NS, there was no statistically significant differences in the biomarkers of oxidative stress (vitamins A and B, albumin, antioxidants, or immune response indicators (p>0.05), with the exception of monocyte counts, which was found to be significantly higher amongst ECU (p<0.05).<br>• Compared to TS, there was no statistically significant differences in the biomarkers of oxidative stress (vitamins A and B, albumin, antioxidants), or immune cells (p>0.05).
(BinShabaib et al. 2019)[26]	• Compared to NS, ECU did not display significantly different mean GCF volume (0.4 [0.1] vs 0.6 [0.2]).<br>• Compared to TS, ECU had significantly lower mean GCF volume (1.3 [0.3] vs 0.6 [0.2]; p<0.05).<br>• Compared to NS, there was no statistically significant difference in pro-inflammatory cytokines amongst ECU.<br>• Compared to TS, pro-inflammatory cytokines were statistically significant and lower amongst ECU, IL-1β (pg/ml) = 139.2 (25.7) vs 69.5 (14.5) , IL-6 (pg/ml)= 26.6 (8.2) vs 7.4(3.6), IFN-γ(pg/ml)= 5.2 ± 2.5 vs 1.4 ± 0.8 , TNF-α (pg/ml) = 108.8 ± 26.4 va 38.2 ± 14.2, and MMP-8 (pg/ml) = 2053.6 ± 247.1 vs 967.4 ± 108.7.
(Cichonska et al. 2019)[27]	• Compared to NS, ECU had significantly lower levels of IgA and lysozyme (p<0.05), but not lactoferrin (p>0.05).<br>• Compared to TS, ECU had significantly higher levels of lactoferrin (p<0.05), but not significant differences in lysozyme and IgA (p>0.05).
(Faridoun et al. 2021)	• Compared to NS, ECU had significantly higher levels of TNF-α (p<0.05).<br>• Compared to NS, ECU did not have significantly different levels of IL-6, IL-8, IL-1β, IL-10, IL-1RA, or c-reactive protein (p>0.05).<br>• Compared to TS, ECU did not have significantly different levels of IL-6, IL-8, IL-1β, TNF-α, IL-10, IL-1RA, or c-reactive protein (p>0.05).<br>• Compared to DU, ECU did not have any significantly different levels of IL-6, IL-8, IL-10, IL-1RA, or c-reactive protein (p>0.05).<br>• Compared to DU, ECU had significantly higher levels of IL-1β (p=0.028),
(Ganesan et al. 2020)[28]	• Compared to NS, ECU demonstrated significantly higher levels of the pro-inflammatory cytokines IL-2, IL-6, GM-CSF, TNF-α, and INF-γ and lower levels of the anti-inflammatory cytokine IL-10.
(Gavrilin et al. 2020)	• There were no significant differences between ECU and TS, NS, or HIV smokers in ASC levels (p>0.05).
(Ghosh et al. 2019)	• NE, MMP-2 and MMP-9 protein levels were significantly elevated in both TS and ECU compared to NS.<br>• Compared to NS, no significant difference in protease levels (A1AT, SLPI, TIMP-1, and TIMP-2) among ECU.
(Ikonomidis et al. 2018)[29]	• Compared to TS, there was no significant differences in levels of MDA amongst ECU, after 30 days of use (p>0.05).

[24] Ashford et al. (2020): Also see in respiratory health
[25] Badea et al. (2019): Also check cardiovascular health
[26] BinShabaib et al. (2019): Immunological indicators were measured as indicators of oral health. Also check oral health.
[27] Cichonska et al. (2019): Immunological indicators were measured from saliva samples for oral antibacterial health outcomes.
[28] Ganesan et al. (2021): Immunological indicators were measured as indicators of oral health. Also check oral Health.
[29] Ikonomidis et al. (2018): Also check cardiovascular health

First author (year)	Findings
(Jackson et al. 2020)	• Compared to NS, ECU had a significant increase in plasma IgE levels (p<0.001), but no significant difference in IgG levels (p>0.05).
(Kelesidis et al. 2020)	• Compared to NS, ECU did not display any significant differences in classical monocytes, intermediate monocytes, non-classical monocytes, inflammatory monocytes, CD3/CD4/ CD8 t-cells, or b-cells (p>0.05), but significantly higher levels of neutrophils (p<0.001), and NK cells (p<0.05).<br>• Compared to TS, ECU did not display any significant differences in neutrophils, classical monocytes, intermediate monocytes, non-classical monocytes, inflammatory monocytes, CD3 or CD4 t-cells, NK cells, or b-cells, but significantly lower levels of CD8 t-cells (p=0.05).<br>• A consistent dose-response result was found, showing an increase in pro-inflammatory monocytes and lymphocytes, lowest in the NS, intermediate in ECU, and highest in TS.
(Lee et al. 2020)	• Compared to NS, exclusive ECU was not significantly associated with increased immune cell type infiltration (naïve B-cells, memory B-cells, plasma cells, CD8 T-cells, CD4 naïve T-cells, CD4 memory resting T-cells, CD4 memory activated T-cells, follicular helper T-cells, regulatory T-cells, gamma-delta T-cells, resting NK cells, activated NK cells, monocytes, M0-M2macrophages, resting dendritic cells, activated dendritic cells, resting mast cells, activated mast cells, eosinophils, neutrophils).
(Mainous et al. 2020)	• Compared to NS, ECU did not have significantly different levels of c-reactive protein (p=0.39).
(Menicagli, Marotta, and Serra 2020)	• Compared to NS (2.77 nM/ml), both ECU groups (e-nicotine and e-vapor groups) had significantly higher mean malondialdehyde (MDA) values (3.14nM.ml p=0.004; 3.11nM/ml p=0.01).<br>• Compared to NS, e-nicotine users had significantly different salivary mucin (SM) levels (p<0.00001), but not e-vapor users (p=0.5).
(Mokeem et al. 2018)[30]	• Compared to NS or TS, there was no significant difference in whole salivary flow rate amongst ECU (0.52ml/min [0.46-0.55]/ 0.53ml/min [0.48-0.55] vs 0.53ml/min [0.5-0.55]) respectively.<br>• Compared to TS, IL-1β (P < 0.01) and IL-6 (P < 0.01) levels were significantly lower among ECU.<br>• Compared to NS, there was no difference in IL-1β and IL-6 levels among ECU.
(Moon et al. 2020)	• Compared to the NS (1.25 (2.50) mg/L) and TS (1.37 (2.46) mg/L), ECU had the highest values for mean hs-CRP level (2.10 (4.14) mg/L), though not statistically significant; p = 0.053).<br>• Compared to the NS (5.94 (1.19) mg/dL) and TS (5.91 (1.32) mg/dL), ECU had the highest values for mean mean uric acid level (6.35 (1.32) mg/dL), though not statistically significant, p = 0.079).<br>• Compared to NS: ECU were at a significantly increased risk of hyperuricemia (aOR 2.67 [1.27-5.58]), hyperuricemia = uric acid of ≥7.0 mg/dL.
(Oliveri, Liang, and Sarkar 2020)[31]	• Compared to TS, ECU didn't have significantly different levels of white blood cells (p=0.06).<br>• Compared to TS, ECU did have significantly lower levels of 11-dehydrothromboxane (p=0.04), 8-epi-prostaglandin (p=0.02) and sICAM-1 (p=0.02).
(Perez et al. 2020)[32]	• Compared to NS, ECU did not demonstrate significantly different levels of white blood cells, neutrophils, lymphocytes, eosinophils, monocytes, or basophils (p>0.05) in the blood sample.<br>• Compared to TS, ECU had significantly less white blood cells and neutrophils present (p<0.05), but not lymphocytes, eosinophils, monocytes, and basophils (p>0.05) in the blood sample.<br>• Compared to both TS and NS, ECU did not demonstrate any significant differences in any immunological outcome in the sputum sample (p>0.05).<br>• Compared to NS, ECU did not have any significantly different levels of TNF-α, IL-6, IL-33, IL-8, IL-10, IL-13, and IL-33 in either serum or sputum samples.<br>• Compared to TS, ECU did not have any significant differences in cytokines in in the serum or sputum samples.

[30] Mokeem et al. (2018): All immunological indicators measured as indicators of oral health. Also check oral health
[31] Oliveri et al. (2020): Also see in cardiovascular health
[32] Perez et al. (2020): Also see in respiratory health

First author (year)	Findings
	• Compared to NS, ECU had significantly high level of YKL-40 ($p<0.05$).
(Pushalkar et al. 2020)[33]	• ECU showed a slightly higher, but not significant difference in TNF-α levels as compared to TS ($p<0.1$).<br>• Compared to both TS and NS, ECU did not exhibit any significant differences in levels of INF-γ, IL-10, IL-12p70, IL-13, IL-1β, IL-2, IL-4, IL-6, IL-8, or TNF-α.
(Reidel et al. 2018)[34]	• Compared to both TS and NS, ECU demonstrated significantly higher primary neutrophil granule proteins, such as NE, proteinase 3, azurocidin, and coronin 1, Neutrophil cell count, however, was not significantly different.<br>• ECU had significantly higher protein arginine deminase 4 compared to NS ($p<0.05$), but not compared to TS ($p>0.05$).<br>• Compared to both TS and NS, ECU demonstrated significantly lower levels of Lysozyme C and DMBT1. However, lactotransferrin and trefoil factor 3 did not significantly differ.<br>• Compared to NS, ECU demonstrated significantly higher levels of MMP-9, S100A8, and S100A9 ($p<0.05$).<br>• Compared to TS, ECU demonstrated significantly higher level of S100A8 ($p<0.05$), However, MMP-9 and S100A9 did not significantly differ ($p>0.05$).<br>• Compared to NS, ECU did not display any differences in mucin MUC5B sputum levels, but did display significantly increased concentrations of MUC5AC ($p=0.05$). As a result, ECU have significantly higher MUC5AC/MUC5B ratios ($p=0.05$) compared to NS.<br>• Compared to TS, ECU did not display any significant differences in Mucin proteins and MUC5AC/MUC5B ratios ($p>0.05$).<br>• Compared to NS, MPO, aldehyde dehydrogenase, thioredoxin, and glutathion S-transferase were significantly higher in ECU ($p<0.05$).<br>• Compared to both TS and NS, ECU did not demonstrate significant differences in nucleobindin 1 and betamicroseminoprotein ($p>0.05$).<br>• Compared to TS, ECU had a significantly higher level of MPO ($p<0.05$). However, aldehyde dehydrogenase, thioredoxin, and glutathione S-transferase did not differ significantly ($p>0.05$).
(Sakaguchi et al. 2021)[35]	• Compared to TS, ECU had significantly lower levels of white blood cells, 11-DHTXB2, 2,3-d-TXB2, sICAM-1 and 8-epi-PGF2α ($p<0.001$).<br>• Compared to NS, ECU users did not have any significantly different levels in white blood cells ($p=0.7$), 11-DHTXB2 ($p=0.09$), sICAM-1 ($p=0.632$), and 8-epi-PGF2α ($p=0.06$). Levels of 2,3-d-TXB2, however, were significantly higher amongst NTV users as compared to NS ($p=0.03$).
(Sakamaki-Ching et al. 2020)	• Compared to NS, ECU had significantly higher levels of metallothionein ($p=0.05$), 8-OHdG ($p=0.01$), and 8-isoprostane ($p=0.03$).<br>• Compared to TS, ECU did not have significantly different levels of metallothionein, 8-OHdG, or 8-isoprostane.
(Shields et al. 2020)	• Compared to NS, ECU had significantly higher proportions of lipid-laden macrophages (LLM) ($p=0.028$).<br>• Compared to TS, ECU had significantly lower proportions of LLM ($p=0.005$).
(Singh et al. 2019)[36]	• Compared to NS, ECU had significantly increased levels of IL-6, IL-8, IL-13, and ICAM-1, but no differences in levels of IL-1β, IL-6, IL-10, IL-33, TNF-α, INF-γ, or GM-CSF.<br>• Compared to NS, ECU displayed significantly increased levels of EGF, VEGF, NGF, PDGF, SCF, HGF, PIGF, but not BDNF, BMP-2, TGC-α, of βFGF/FGF2.<br>• Compared to NS, ECU displayed significantly higher levels of MMP-9, and significantly lower levels of RAGE, but no significant differences in S100A8, S100A9, C10, Galectin-3, EN-RAGE, or CC-16.<br>• Compared to NS, ECU showed significantly lower levels of CXCL1, but not MCP-1, MIP-1α, MIP-1β, RANTES, Eotaxin, CXCL2, G-CSF, GCSF, leukotriene E4, desmosine, and PAI-1/Serpine-1.

[33] Pushalkar et al. (2020): Immunological indicators were measured as indicators of oral health.

[34] Reidel et al. (2018): All indicators were collected via sputum and measured as an indicator of respiratory/immunological health

[35] Sakaguchi et al. (2021): Also check respiratory and cardiovascular health

[36] Singh et al. (2019): Also see in Respiratory Health

First author (year)	Findings
	Compared to NS, ECU displayed significantly lower levels of Resolvin D1, and resolving D2, and significantly higher levels of 8-isoprostrane (in urine, not-sig in plasma), and 8-oxo-dG, but no significantly differences, in 4-hydroxynonenal, resolvin E1, malondialdehyde, or MPO.
(Sinha et al. 2021)[37]	• Compared to NS, ECU had significantly higher mean levels of PISF volume (1.5 [0.5] vs 3.17 [0.6]; p<0.01).<br>• Compared to NS, exclusive ECU displayed significantly higher levels of TNF-α (p<0.001) and IL-1β (p<0.01) in the peri-implant region fluid.
(Song, Freudenheim , et al. 2020)[38]	• Compared to NS, ECU did not display significant differences in IL-2, IL-8, IL-12, TNF-α, IL-4, IL-10, IL-13, macrophages, lymphocytes, neutrophils, or eosinophils (p>0.05).<br>• Compared to NS, ECU saw significantly higher levels of IL-1β (p<0.005), and IL-6 (p<0.02), and significantly lower levels of INF-γ (p<0.02).<br>• Compared to TS, ECU did not display significant differences in IL-2, IL-6 IL-8, IL-12, TNF-α, INF-γ, IL-4, IL-10, IL-13, neutrophils, or eosinophils.<br>• Compared to TS, ECU displayed significantly lower levels of IL-1β (p<0.0001), macrophages (p<0.02), and lymphocytes (p<0.03).
(Stokes et al. 2021)[39]	• Compared to NS, exclusive ECU were not at a significantly differential odds of having hs-CRP levels (aOR 1.08 [0.92-1.27]), IL-6 (aOR 1.00 [0.89-1.12]), sICAM-1 (aOR 1.05 [0.99-1.11]), fibrinogen (aOR 1.00 [0.96-1.04]), or urinary 8-isprostane (aOR 1.02 [0.89-1.17]).<br>• Compared to TS, exclusive ECU were not at a significantly differential odds of having hs-CRP levels (aOR 0.91 [0.79-1.07]), but did display significantly lower levels of IL-6 (aOR 0.87 [0.78-0.98]), sICAM-1 (aOR 0.88 [0.83-0.93]), fibrinogen (aOR 0.96 [0.92-0.99]), and urinary 8-isprostane (aOR 0.82 [0.72-0.93]).
(Ye et al. 2020)[40]	• Compared to NS, ECU did not demonstrate any significant differences in IL-1β (p=0.36), PGE2 (p=0.90), EN-RAGE (p=0.64), RAGE (p=0.33), S100A8 (p=0.09), S100A9 (p=0.55), galectin-3 (p-0.64), uteroglobin/CC-10 (p=0.22), serpinne1/PAI- (p=0.41), BDNF (p=0.14), Basic-FGF (p=0.36), Beta-NGF (p=0.38), SCF (p=0.50), BMP-2 (p=0.60), HGF (p=0.58), PDGF-AA (p=0.14), TGF- α (p=0.45), EGF (p=0.14), PIGF (p=0.41), VEGF (p=0.48).<br>• Compared to NS, ECU had significantly lower levels of MMP-9 (p=0.04) and MPO (p=0.002).<br>• Compared to TS, ECU did not demonstrated any significant differences in IL-1β (p=0.11), EN-RAGE (p=0.50), RAGE (p=0.73), MMP-9 (p=0.73), MPO (p=0.57), S100A8 (p=0.99), S100A9 (p=0.94), galectin-3 (p=0.93), uteroglobin/CC-10 (p=0.55), serpine1/PAI-1 (p=0.87), BDNF (p=0.69), Basic-FGF (p=0.77), Beta-NGF (p=0.78), SCF (p=0.53), BMP-2 (p=0.42), HGF (p=0.63), PDGF-AA (p=0.57), TGF-α (p=0.78), EGF (p=0.31), PIGF (p=0.75), VEGF (p=0.55).<br>• Compared to TS, ECU did have significantly lower levels of PGE2 (p=0.006).<br>• Compared to DU, ECU did not demonstrate any significant differences in IL-1β (p=0.89), PGE2 (p=0.84), EN-RAGE (p=0.14), MMP-9 (p=0.11), S100A8 (p=0.59), S100A9 (p=0.19), galectin-3 (p=0.09), serpine1/PAI-1 (p=0.13), BDNF (p=0.52), Basic-FGF (p=0.34), Beta-NGF (p=0.40), SCF (p=0.26), BMP-2 (p=0.42), HGF (p=0.60), PDGF-AA (p=0.41), TGF- α (p=0.39), EGF (p=0.40), PIGF (p=0.23), VEGF (p=0.32).<br>• Compared to DU, ECU had significantly lower levels of RAGE (p=0.04), MPO (p=0.03), and uteroglobin/CC-10 (p=0.03).

[37] Sinha et al. (2021): All immunological biomarkers were measured as a indicator of oral health/inflammation via PISF. Also check oral health

[38] Song et al. (2020): All immunological indicators were measured as an indicator of respiratory health/injury/inflammation

[39] Stokes et al. (2021): All immunological indicators were measured in the context of cardiovascular health

[40] Ye et al. (2020) all immunological indicators were measured as indicators of oral health

Table S3.3: Oral health

First author (year)	Findings
**Oral health (n=23)**	
**Cohort studies (n=3)**	
(ALHarthi et al. 2019)	• Compared to NS, ECU displayed no significant difference in PI, BOP, and PD at 3 months' (P > 0.05) and 6-months' (P > 0.05) follow-up. No pockets with PD ≥ 4 mm at 3 and 6 months' follow-up.<br>• No significant difference in numberof missing teeth and none of the individuals in the study exhibited CAL.<br>• At baseline, no statistically significant difference between TS and ECU in mean PI (cs= 49.4 ± 7.3, v= 43.5 ± 5.6) ((P > 0.05)), BOP (cs= 17.2 ± 3.3 , v= 11.6 ± 4.5) (P > 0.05), PD (cs= 5.2 ± 0.4, v=4.6 ± 0.2) (P > 0.05), and number of sites with PD ≥ 4 mm (ns= 14.2 ± 1.5, v= 10.6 ± 1.2) (P > 0.05).<br>• At 3 and 6 months' follow-up, PI (P < 0.05) and PD (P < 0.05) were significantly higher in TS (PI_3= 34.5 ± 4.6, PD_3 = 4.4 ± 0.4) compared to ECU (PI_3= 43.5 ± 5.6, PD_3 = 21.4 ± 2.8).<br>• TS had 7.1 ± 0.6 and 7.4 ± 0.5 sites with PD ≥ 4 mm at 3 and 6 months' follow-up, whereas, ECU had no sites with PD ≥ 4 mm at 3 and 6 months' follow-up.<br>• No statistically significant difference in BOP between TS and ECU at all time intervals.<br>• Between ECU and NS, no significant difference were observed in the mean PI (P > 0.05), PD (P > 0.05), and number of sites with PD ≥ 4 mm (P > 0.05) at baseline.<br>• BOP was significantly higher in NS (38.2 ± 6.5) compared with ECU (11.6 ± 4.5) (P < 0.01).
(Atuegwu et al. 2019)	• ECU had increased odds of being diagnosed with gum disease (OR 1.76, 95% CI 1.12–2.76), bone loss around the teeth (OR 1.67, 95% CI 1.06–2.63) and any periodontal disease (OR 1.58, 95% CI 1.06–2.34) compared to NS (never ECU) after adjusting for longitudinal cigarette use and other confounding factors.
(Ghazali, Ismail, and Daud 2019)	• No significant difference in the mean DMFT index between TS = 4.09 (95% CI, 3.14, 5.04), NS= 3.51 (2.56, 4.463) and ECU= 3.13 (2.18, 4.09) at baseline (p = 0.370) nor at the 6-months follow-up (TS = 4.68 (3.00, 5.71), NS= 4.36 (3.00, 5.71) and ECU= 3.54 (2.17, 4.91) (p =0.480)
**Case-control studies (n=2)**	
(Ibraheem et al. 2020)[41]	• Compared to NS ECU had significantly higher RANKL levels (3.5 ± 0.7 pg/ml) vs. (11.5 ± 8.4 pg/ml)), and OPG levels (21.5 ± 10.7 pg/ml) vs. (77.5 ± 3.4 pg/ml) in their GCF fluid (p<0.01).<br>• Compared to TS, there was no difference in the RANKL or OPG levels amongst ECU (p>0.05).<br>• Compared to NS, ECU had significantly higher PI: mean (SD) 15.2 (2.1%) vs 40.1 (2.2%) (P < 0.01), PD: 1.8 (0.08) vs 4.2 ± 0.2% (P < 0.01), CAL: 0.4 (0.05) vs 2.5 (0.2) (P < 0.01) and MBL on the mesial 1.5 (0.1) vs 4.4 (0.4) (P < 0.01) and distal 1.4 (0.08) vs 4.4 (0.5) (P < 0.01) surfaces of teeth.<br>• No significant difference in BOP among individuals in all groups.
(Karaaslan, Dikilitas, and Yigit 2020)[42]	• No significant differences between TS and ECU for mean CAL, PD and PI, p >0.05. Mean GI score of TS (1.53 (SD 0.29)) was significantly lower than ECU (1.81 (SD 0.30)) p=0.001.
**Cross sectional studies (n=18)**	
(Aherrera et al. 2020)[43]	• Compared to NS, ECU did not differ in the number who self reported dental discolouration or gingival inflammation.

[41] Ibraheem et al. (2020) : Also check immunological health
[42] Karaaslan et al. (2020) : Also check immunological health
[43] Aherrera et al. (2020): Also see in respiratory, cardiovascular, and immunological health

First author (year)	Findings
(Akinkugbe 2019)	• Compared to NS, the odds of ECU being r diagnosed with dental problems in the past- year is 1.11 (95% CI. 0.79 to 1.55) for current users, and 1.12 (95% CI. 0.9 to 1.38) for ECU ever users.<br><br>• Compared to NS, the odds of ECU being ever diagnosed with dental problems is 1.27 (95% CI. 0.95 to 1.70) for current users, and 1.28 (95% CI. 1.07 to 1.54) for ECU ever users.
(Al Deeb et al. 2020)[44]	• Compared to TS plaque index (PI) was significantly lower in ECU (45.3 (12.6) vs.37.6 (8.0) p=0.01), but PI did not differ when comparing ECU to NS (32.4 (14.7), p=0.15)<br>• Compared to TS, bleeding on probing (BOP) was significantly higher in ECU (13.8 (2.4) vs 18.6 (3.7), p=0.001, but significantly lower when compared to NS 37.4 (6.5) p=0.001<br>• Compared to NS, probing depth (PD) (in millimetres) was significantly higher in ECU (3.5 (0.4) vs. 3.8 (0.6)) p = 0.0491 but there no significant difference compared to TS (4.1 (0.8)).
(Al-Aali et al. 2018)[45]	• Compared to NS, PI (percentage of sites) was significantly higher in ECU (47.6 (9.6) vs. 52.6 (11.9))<br>• Compared to NS, BOP (percentage of sites) was significantly lower in ECU (39.8 (18.1) vs 24.7 (5.3)).<br>• Compared to NS, PD (in millimetres) was significantly higher in ECU (4.5 (0.7) vs.5.9 (1.4)).<br>• ECU results for peri-implant bone loss (PIBL) (in millimetres) mesial 1.6 (0.7) and distal 2.1 (1.0) were significantly higher compared to NS 0.8(0.2), 1.1 (0.5), respectively.
(Aldakheel et al. 2020)	• PI (P < 0.001), GI (P < 0.001), CAL (P < 0.001), PD (P < 0.001) and mesial (P < 0.001) and distal (P < 0.001) MBL were significantly higher among TS, ECU, and NS with periodontitis compared with NS without periodontitis. No statistically significant difference in these measures were observed among TS, ECU, and NS with periodontitis.<br>o TS: PI= 2.2 ± 0.3, GI= 1.4 ± 0.2, CAL= 3.5 ± 0.2, PD= 4.5 ± 0.4, MBL (mesial= 4.2 ± 0.7, MBL distal= 4.1 ± 0.5),<br>o ECU: PI= 2.6 ± 0.6, GI= 1.2 ± 0.3, CAL= 3.1 ± 0.06, PD= 4.1 ± 0.07, MBL (mesial= 4 ± 0.08), MBL distal= 3.9 ± 0.07),<br>o NS without periodontitis: PI= 0.4 ± 0.06, GI= 0.3 ± 0.02, CAL= 0.3 ± 0.02, PD= 1.1 ± 0.3, MBL (mesial= 0.4 ± 0.05, MBL distal= 0.4 ± 0.03)<br>o NS with periodontitis: (PI= 2.3 ± 0.4, GI= 1.7 ± 0.3, CAL= 3.2 ± 0.04, PD= 4.5 ± 0.2, MBL (mesial= 4.2 ± 0.2, MBL distal= 4.2 ± 0.2).<br><br>• No statistically significant difference in the overall full mouth periodontal parameters and mandibular 1st molar sties among the four groups.
(Al-Hamoudi et al. 2020)[46]	• At baseline GI was significantly lower in ECU 0.36 (0.08), compared to NS 2.55 (0.08), but no significance difference in all other indicators between the two groups (ECU vs NS):<br>o PD(mm) : 5.2 (0.87) vs. 5 (0.06)<br>o CAL (mm): 3.4 (0.2) vs. 3.1 (0.2)<br>o MBL: Mesial 4.8 (0.3) vs. 4.7 (0.2) and distal 4.6 (0.2) vs 4.6 (0.1).
(AlQahtani et al. 2018)[47]	• Mean PI (P < 0.05), PD ≥ 4 mm (P < 0.05), and total radiographic bone loss (RBL) (P < 0.01) was significantly higher among **TS** (PI =67.4 (SD 7.5), PD= 7.8 (1.2), RBL = 3.6 (0.5)), **ECU** (PI =51.9 (10.2), PD= 5.3(1.5), RBL = 1.0 (0.9)) compared with **NS** (PI = 34.1 (14.7), PD= 4.4 (0.6), RBL = 0.9 (0.3)).<br>• BOP were significantly lower in TS 16.7 (3.9), and ECU 23.3 (5.1) compared to NS 38.9 (19.6) (P < 0.01).<br>• PD ≥ 4 mm, 7.8 (1.2) and RBL 3.6 (0.5) were significantly higher in TS compared to ECU PD=5.3 (1.5), and RBL= 1.9 (0.9) (P <0.05).<br>• No significant differences between TS and ECU for PI and BOP measures.

[44]Al Deeb et al. (2020): Also check immunological health
[45] Al-Aali et al. 2018: Also check immunological health
[46] Al-Hamoudi et al. (2020): Also check immunological health
[47] AlQahtani et al. (2018): Also checkI immunological health

First author (year)	Findings
(F. Alqahtani et al. 2019)[48]	• Compared with NS, PI ($P < 0.05$) and PD ($P < 0.05$) were significantly higher among ECU (PI= 12.6 (1.1) vs. 27.2 (2.4) and PD= 0.8 (0.1) vs. 3.2 (0.3).<br>• Compared with TS, PI ($P < 0.05$) and PD ($P < 0.05$) were significantly lower among ECU (PI= 38.6 (3.9), vs. 27.2 (2.4) and PD= 4.3 (0.2) vs. 3.2 (0.3).<br>• Peri-implant BOP was more often manifested in NS (19.8 (1.3) compared with TS (6.8 (1.2)) ($P < 0.05$), and ECU (6.6 (1.3)) ($P < 0.05$).
(ArRejaie 2019)[49]	• ECU results for PI, 43.5 (8.1) and MBL, 1.4 (0.9) were significantly higher compared to NS, PI=29.7 (5.2), MBL=0.9 (0.3), but significantly lower than TS for both PI=56.4 (12.3), and MBL=2.3 (1.2) ($P<0.01$).<br>• ECU results for BOP, 14.7 (5.3) and PD, 15.9 (1.4) were lower compared to NS (BOP=39.8 (18.1), PD=4.5 (0.7), but insignificant when compared to TS, BOP, 18.4 (4.8), PD, 23.8 (3.7).
(BinShabaib et al. 2019)[50]	• BOP was significantly lower in ECU 12.2% (range: 14.4% – 20.5%) compared to NS 28.4% (26.3%–33.4%) ($p<0.05$).<br>• No statistically significant difference in PD, PI, MBL, and missing teeth in ECU compared to NS
(Huilgol et al. 2019)	• ECU, was independently associated with a 78% higher odds of poor oral health (adjusted OR = 1.78, 95% CI: 1.39–2.30; $P < 0.001$) compared to NS.
(Javed et al. 2017)	• BOP was significantly lower in the ECU (4.6 (2.9) compared to NS 27.5(3.2) ($p<0.01$)<br>• ECU results for PI=23.3 (3.4) and PD=5.1 (1.2) were significantly lower than TS for both PI =52.1 (6.6) and PD =29.3 (1.7) ($p<0.01$).<br>• Compared to ECU, TS had significantly higher self perceived gingival pain (OR 21.4 (19.6 to 25.5), $p <0.01$) and swelling (OR = 5.2 (4.7 to 5.6), $p<0.01$); but TS had lower gingival bleeding (OR = 0.6 (0.4 to 0.8), $p<0.05$).<br>• Compared to NS, ECU had significantly lower self perceived gingival pain (OR 0.6 (0.3 to 0.8), $p <0.05$) and swelling (OR = 0.6 (0.5 to 0.8), $p<0.01$) but higher gingival bleeding (OR = 3.1 (2.7 to 3.3), $p<0.01$).
(Jeong et al. 2019)	• ECU males, but not females, demonstrated a higher risk (2.34 (1.52-3.59) of periodontal diseases compared to NS (reference).
(Mokeem et al. 2018)	• There was no significance difference in PI, PD, CAL, mesial and distal MBL among ECU compared to NS.<br>• BOP was significantly lower in ECU compared to NS.<br>• No significance difference in unstimulated whole salivary rate between ECU and NS was observed.
(Mokeem et al. 2019)	• C. albicans carriage was significantly higher in TS 64.7%, 95% CI (64, 65.2) ($p < 0.05$), and 50% , 95% CI (49.6, 50.2) ($p < 0.05$) compared to NS 31.2%, 95% CI (30.9, 31.4).<br>• ECU had significantly lower average number of missing teeth 4.8(0.5) compared to NS 5.2(0.5) p= 0.002 and TS 5.8(0.6) p=0.001<br>• No significant difference in unstimulated whole salivary flow rate (ml/min) for ECU 0.42 (0.2) compared to NS 0.43 (0.3) p= 0.87 nor TS 0.42 (0.3) p=1.00
(Sinha et al. 2021)[51]	• Mean bleeding on probing (BOP) was significantly lower in ECU 23.7 (5.3), compared to NS 37.82 (17.8) $P<0.001$<br>• PD was significantly higher in ECU 5.87(1.4) compared to NS 4.65(0.71)<br>• PIBL was significantly higher in ECU including total 1.78 (0.9), mesial 1.56 (0.7) and distal 2.09 (1.0), compared to NS 0.93(0.3), 0.98±0.2, 1.12±0.5), respectively, $P<0.05$

48 AlQahtani et al. (2019): Also check immunological health
49 ArRejaie et al. (2019): Also check in immunological health
50 BinShabaib et al. (2019): Also check in immunological health
51 Sinha et al. (2021): Also check immunological health

First author (year)	Findings
	• No significant difference in PI between ECU and NS.
(Vohra et al. 2020)	• Compared to ENDS (reference group), the odds of reporting pain in gums (OR=4.8 (2.64–5.57), p <.001) was higher among TS, however, there was no significant difference in the odds of pain in teeth (OR= 0.7(0.43–0.81) p=0.12) and bleeding gums (OR=0.59 (0.47-0.62), p=0.16) between ENDS and TS.<br>• Compared to NS, the odds of reporting pain in teeth (OR=2.8 (1.73–3.14), p<0.01) and bleeding gums (OR=1.54 (1.2–2) (p<0.05) were higher among ENDS, however, there was no significant difference in the odds of pain in gums between ENDS and NS (OR=0.35 (0.08-0.41), p=0.26).<br>• No statistically significant difference in self-rated pain in teeth, bleeding gums, and pain in gums between neither JUUL users and NS nor JUUL users and ENDS.<br>• Groups estimates for PI (p<0.05) and PD (p<0.05) were significantly higher among TS (PI= 39.3(8.2), PD= 4.2 (0.5)) compared to ENDS (PI=25.6 (6.2), PD=1.5 (0.3)) and JUUL-users (PI=26.5(5.8), PD=1.6 (0.2)).<br>• Compared to NS, ENDS had significantly lower missing teeth (4.2 (0.5) vs. 3.6 (0.5), p<0.001), BOP (22.1(3.3) vs. 11.5 (0.8) p<0.001), and MBL (mesial 1.2 (0.3) vs (0.8(0.06) and distal (1.2 (0.2) vs 0.7 (0.05)), but higher PI (16.6 (2.1) vs. 25.6 (6.2), p<0.001).<br>• Compared to NS, JUUL-users had significantly lower missing teeth (4.2 (0.5) vs. 3.3 (0.4), p<0.001), and BOP (22.1(3.3) vs. 10.5 (2.2) p<0.001), and MBL (mesial 1.2 (0.3) vs (1(0.08) and distal (1.2 (0.2) vs 1.1 (0.1)), but higher PI (16.6 (2.1) vs. 26.5 (5.8), p<0.001). No statistically significant difference in bleeding gums between TS and ENDS.<br>• No statistically significant difference in CAL between the study groups.
(Vora and Chaffee 2019)	• Compared to NS, ECU had a higher odds of diagnosis of gingivitis (AOR=2.9; 95% CI, 1.9 - 4.5) and gingivitis disease treatment (AOR=2.3; 95% CI, 1.3 - 2.4) similarly to TS (AOR=2.2; 95% CI, 1.9 - 2.6) when compared to NS.<br>• Compared to NS, ECU had a higher odds of reporting of gingival disease treatment (AOR=2.3; 95% CI, 1.3 - 2.4), similarly to TS (AOR=1.5; 95% CI, 1.3 - 1.7) when compared to NS.

Table S3.4: Respiratory health

First author (year)	Findings
**Respiratory health (n=32)**	
**Randomized controlled trials (n=4)**	
(Haziza et al. 2020)[52]	• There was no significant differences in FEV1 measured between ECU and TS (p=0.7) and ECU and NS (p=0.6) after 90 days post-randomization.
(Ludicke et al. 2018)[53]	• The study found no significant difference in lung function, as measured by FEV1, between ECU, and the NS (p=0.9848) and TS (p=0.0669).
(Ludicke et al. 2019)[54]	• Compared to continuing TS, switching to ECU (HNB) resulted in a significantly better FEV1 outcomes at 6 month follow-up (p= 0.008).

[52] Haziza et al. (2020): Also check cardiovascular, and immunological.
[53] Ludick et al. (2020): Also check immunological health.
[54] Ludick et al. (2019): Also check immunological and cardiovascular health

First author (year)	Findings
	• For DU (who did not switch totally to ECU), a dose-response gradient was seen in the improvement of FEV1 amongst predominant ECU at month 6 follow-up, demonstrating that the higher the cigarette exposure, the lower the FEV1% values (worse outcome) among 4 quartiles of exposure measured through biomarker of exposure (2-cyanoethylmercapturic acid (CEMA quartiles)) (Q1 ((bottom): 95.7; Q2:95.2; Q3: 94.0; Q4 (top): 93.1). Higher CEMA levels are indicative of higher levels of cigarette smoking.<br>• A significantly decreased difference in regular cough was found at 6 months among ECU compared to TS (OR=0.6 [0.4–0.9]).
(Pulvers et al. 2020)[55]	• Compared to TS, ECU did not show significant different risks of respiratory symptoms (RR 0.61 [0.34-1.09]) or lung function (FEF25-75) (RR 1.01 [0.76-1.34]) at week 6.<br>• Compared to the DU, ECU did not show significantly different risks of respiratory symptoms (RR 0.84 [0.56-1.26]) or lung function (FEF25-75) (RR 1.07 [0.88-1.31]) at week 6.
**Cohort studies (n=5)**	
(Dai and Khan 2020)	• The prevalence of respiratory symptoms at Wave 2 by baseline e-cigarette use status showed higher prevalence among ECU (33.6% [95% CI 26.7% to 41.4%] and DU (50.8% [47.4% to 54.2%]) compared to NS (21.7% [19.2% to 24.4%]).<br>• Compared to NS, wheezing or whistling in the chest, having chest wheeziness during or after exercise, and having a dry cough at night in the past 12 months were also found to be significantly worse amongst ECU and DU compared to NS.
(Polosa et al. 2020)	• Compared to their baseline TS status, participants that were ECU at 60 months follow-up showed significant within-group improvement in FEV1 (p=0.003) and FVC (p=0.003) measurements. They did not show a significant improvement in CAT score (p=0.065) or FEV1/FVC ratio (p=0.063).
(Polosa et al. 2018)	• Compared to their baseline TS status, participants that were ECU showed no significant changes in FEV1 (p=0.254) and FVC (p=0.277) at 36 month follow up.
(Polosa et al. 2017)	• Compared to NS,, no significant changes could be detected over the observation period amongst ECU in FEV1 (p=0.3), FVC (p=0.61), FEV1/FVC (p=0.09), FEF25-75 (p=0.4), CO (p=0.21), or FeNO (p=0.9)<br>• None of the participants in the study reported any respiratory symptoms in either group, therefore no significant differences were seen.
(Tackett et al. 2020)	• Compared to adolescents NS, there was no significantly increased odds of wheezing among those that reported e-cigarette use in the past 12 months (aOR 1.37 [0.91-2.05]), past 30 day (aOR 1.35 [0.63-2.88]) or past 7 day users (aOR 0.74 [0.28-1.97]) at Wave 4.
**Cross-sectional studies (n=22)**	
(AboElNaga 2018)	• Compared to NS, significant differences in lung function were seen amongst both TS and ECU. (FVC p=0.02; (FEV1, FEV1/FVC ratio, MMEF, PEFR, ACT p<0.001).<br>• No significant difference in outcomes between TS and ECU were observed (p>0.05).
(Aherrera et al. 2020)[56]	• Compared to NS, ECU had significant higher rates of wheezing (p=0.02)<br>• There were no significant differences in cough, phlegm, difficulties breathing, between ECU and NS.

[55] Pulvers et al. (2020): Also check in cardiovascular health
[56] Aherrera et al. (2020): Also check oral, cardiovascular, and immunological health

First author (year)	Findings
(Alnajem et al. 2020)	• Compared to NS, both ECU (aPR 1.54 (1.01–2.45); 1.52 (1.05–2.21)), and DU (aPR: 1.87 (1.44–2.42)) were at an increased odds of currently wheezing.
(Ashford et al. 2020)[57]	• Compared to NS, ECU were not more likely to report persistent cough (aOR 8.0; p=0.07)
(Boddu et al. 2019)	• Reporting past 30 day chronic cough increased from 39.7% among NS, to 50.6% among ECU, 60.0% among TS and 63.6% among DU.<br>• Amongst individuals who reported a cough in the past 30-days, DU had lower cough-symptom scores (LCQ scores) compared with ECU (mean difference = -1.65 [-3.00—0.30]; p=0.017) and NS (-1.31 [-2.63-0.01]; p=0.05)<br>• Compared to NS, ECU use was not associated with coughing in the past 30 days.
(Bowler et al. 2017)	• Compared to never users, there was no significant difference in FEV1 measurements amongst current e-cigarette users (P>0.05) in neither COPDGene cohort or SPIROMICS cohort
(Braymiller et al. 2020)	• Compared to NS, there was no significant differences in odds of any respiratory symptoms amongst occasional ECU (bronchitic symptoms: 1-2 days/month: 1.13 (0.71-1.81); >3 days/month: 0.96 (0.63-1.46); wheeze: 1-2 days/month 0.99 (0.59-1.65); >3 days/month 0.85 (0.54-1.35); shortness of breath: 1-2 days/month 1.31 (0.86-1.99); >3 days/month 0.96 (0.64-1.42)) or lifetime ECU (bronchitic symptoms: past 6 months 0.82 (0.50-1.32); >6 months: 1.21 (0.76-1.93); wheeze: past 6 months 1.06 (0.66-1.71); > 6 months 0.96 (0.58-1.59); shortness of breath: past 6 months 1.10 (0.73-1.67); >6 months 1.03 (0.68-1.57).
(Brozek, Jankowski, and Zejda 2019)	• Compared to NS, no significant differences were seen amongst ECU or DU, in regards to FEV1, FVC, FEV1/FVC, PEF, MFEF245-75, or O2 saturation (p>0.05).
(Cassidy, Tidey, and Colby 2020)	• Compared to DU, ECU were significantly less likely to experience coughing (p<0.001), wheezing (p=0.003), shortness of breath (p=0.004), phlegm production (p<0.001), and tightness in chest (p=0.05).
(Diamantopoulou et al. 2019)	• Compared to DU, no significant differences in respiratory symptoms (cough, dyspnea) were observed in ECU (p>0.05).
(Ghosh et al. 2019)[58]	• Compared to both NS and TS, there was no significant differences in FEV1 and FVC amongst ECU (p>0.05)<br>• NE, MMP-2 and MMP-9 protein levels were significantly elevated in both TS and ECU compared to NS.<br>• No significant difference in protease levels between ECU vs TS was observed.
(Giovanni et al. 2020)	• Compared to NS, ECU were at significantly increased odds of having any respiratory symptoms in the youngest age group (18-34) (PR 1.36 [1.08–1.70]), but not amongst older age groups. This association is thought to be driven by a higher independent prevalence of cough (PR 1.60 [1.11–2.31])
(Hedman et al. 2018)	• Compared to NS, having any respiratory symptom was significantly associated with DU (aOR, 4.03; 95% CI, 3.23-5.02), but not ECU (aOR, 1.46; [0.93-2.29])<br>• Compared to NS, ECU were significantly more likely to have a cough (aOR: longstanding cough 1.60 [0.92-2.80]/ productive cough 0.62 [0.15-2.51]) regardless of former smoking status.<br>• Compared to NS, ECU were significantly more likely to report wheezing if they were a former smokers (aOR 1.73 [1.01-2.97]), but not amongst ECU that had never smoked (aOR: 0.87 [0.46-1.64])

[57] Ashford et al. (2020): Also see in immunological health
[58] Ghosh A et al. (2019): Also check in immunological heath

First author (year)	Findings
	• Compared to NS, ECU were significantly more likely to have sputum production if they had never smoked (aOR 1.96 [1.18-3.24]), but not amongst ECU who were also former smokers (aOR 1.12 [0.59-2.12])
(Kizhakke Puliyakote et al. 2020)	• There was no significant difference in mean alveolar ventilation between NS and ECU at baseline (P = 0.21)<br>• Mean alveolar perfusion between NS and ECU at baseline (P = 0.43) was not significantly different<br>• Perfusion heterogeneity (Relative dispersion) was significantly elevated in ECU at baseline compared with NS (P = 0.04)<br>• ECU had significantly greater ventilation-perfusion heterogeneity relative to NS, (P = 0.01), which indicated significantly worse outcomes<br>• Significant differences were not seen between SpO2, peripheral oxygen saturation (p=0.9) and forced vital capacity (FVC, L) p= 0.20<br>• Significantly larger differences were seen amongst forced expiratory volume (FEV1, L) (p= 0.05) and FEV1/FVC (p=0.006), which indicated better outcomes for ECU
(Li and Xie 2020)	• Compared to NS, ever-wheezing was not significant amongst 1 time vape-users (aOR 1.1 [0.9 - 1.4]) and 51-99 time users 1.4 [0.9 - 2.2]) but was significantly higher amongst individuals who vaped 2-10 times (1.4 [1.1- 1.6]), 11-20 times (1.9 [1.4 - 2.5]), 21-50 times (1.4 [1.1 - 1.7), and 100 times + users (1.4 [1.0 to 2.0]).<br>• Compared to NS, individuals who ever used an e-cigarette at any frequency did not have a significantly increased odds of coughing in the past 12 months (1 time: 1.0 [0.8 to 1.2]; 2-10 times: 1.1 [0.9 to 1.4]; 11-20 times: 1.1 [0.8 to 1.4]; 21-50 times: 1.0 [0.8 to 1.3]; 51-99 times: 1.0 [0.6 to 1.7]; 100+ times: 1.1 [0.8 to 1.5]).
(Li et al. 2019)	• Compared with NS, the odds of wheezing and related respiratory symptoms were significantly increased in ECU (aOR 1.67 [1.23 to 2.15]).<br>• Compared with TS, ECU had significantly lower risk in wheezing and related respiratory symptoms (aOR 0.68 [0.53 to 0.87]).
(Meo et al. 2019)	• Compared to NS, ECU has significantly worse lung function test parameters FEV1; FEV1/FVC ratio; FEF25%; FEF50%; FEF75%; FEF25%–75%; FEF75%–85% (p<0.05).<br>• There was no significant difference in FVC and PEF between NS vs ECU (p>0.05).
(Perez et al. 2020)[59]	• No significant difference at baseline between NS, ECU and TS for produced sputum, FEV1, FVC, FEV1/FVC ratio, and FEV1 change after bronchodilator (p >0.05).<br>• Serum YKL-40 was elevated in ECU (21.2 [12.1-24.0]) in comparison with the NS (12.2 [8.7-18.1]; p=0.04).<br>• There were no significant differences in the sputum YKL-40 levels between all groups (p=0.552).
(Sakaguchi et al. 2021)[60]	• Compared to TS, cough (p<0.001), FEV1 (p=0.04), and FEF25- 75 (p=0.03), were significantly better amongst ECU<br>• Compared to TS, ECU did not demonstrate significantly different levels in terms for FVC (p = 0.169), FEV1/FEV ratio (p=0.2), and PEF (p=0.07)<br>• Compared to NS, ECU did not differ significantly in terms of cough (p=0.3), FVC (p=0.07), FEF25-75 (p=0.2), FEV1/FVC (p=0.5) PEF (p=0.6) or FEV1 (L) (p=0.09).
(Schneller et al. 2020)	• Compared to NS, ECU who never smoked were not at a significantly different odds of ever wheezing (aOR 0.73 [0.34-1.54]) or wheezing in the past 12 months (aOR 0.74 [0.28-1.96])

[59] Perez et al. (2020): Also check immunological health
[60] Sakaguchi et al. (2021): Also check immunological, and cardiovascular

First author (year)	Findings
	• Compared to NS, ECU who were former smokers had significantly increased odds of ever wheezing (aOR 1.99 [1.47-2.69]) or wheezing in the past 12 months (aOR 1.94 [1.29-2.90])<br>• Compared to exclusive ECU, TS had significantly higher odds of ever wheezing (aOR 1.75 [1.33-2.32]).
(Singh et al. 2019)[61]	• Compared to NS, there were no significant differences in measures of lung function (FEV1, FVC, FEV1/FVC, and PEF) amongst ECU (p>0.05).
(Wang et al. 2018)[62]	• Compared to NS, ECU was associated higher breathing difficulty scores (typically and past month) (p< .0001), and greater proportions of those who responded 'yes' to having chest pain (p< .0001).
(Xie et al. 2020)	• Compared to NS, ever-users of e-cigarettes were not significantly more likely to wheeze (aOR 1.04 [0.92- 1.16]) or cough (aOR 0.98 [0.89 - 1.08])<br>• Compared to NS, ever-users of e-cigarettes were not significantly more likely to have a lifetime history of any respiratory condition (aOR 0.95 [0.86 -1.04])

[61] Singh et al. (2019): Also check Immunological health
[62] Wang JB et al. (2019): Also check cardiovascular
