# Supplementary material for: Chronic health effects associated with electronic cigarette use: A systematic review
Source: Front Public Health. 2022 Oct 6;10:959622. doi: 10.3389/fpubh.2022.959622 (PMC9584749; doi:10.3389/fpubh.2022.959622)
Supplement: Supplementary file 4 [file Table_4.pdf]

Supplement S4: Risk of bias assessment for daily e-cigarette use studies

Item description of the modified critical appraisals skills programme (CASP) checklist:

Randomized controlled trials and pre-post studies:

- **Are the results valid?** 1.Did the study address a clearly focused issue? 2. Was the assignment of patients/ participants to treatments randomised? 3. Were all of the patients/participants who entered the trial properly accounted for at its conclusion? 4. Were patients, health workers and study personnel 'blind' to treatment? 5. Were the groups similar at the start of the trial 6. Aside from the experimental intervention, were the groups treated equally?
- **What are the results?** 7a. was the treatment effect large? 7b. how large was the treatment effect? 8a. the estimate of the treatment effect was precise. 8b. how precise was the estimate of the treatment effect?
- **Will the results help locally?** 9. Can the results be applied to the local population?

Cross-sectional and cohort studies:

- **Are the results valid?** 1.Did the study address a clearly focused issue? 2 .Was the (sample/cohort) recruited in an acceptable way? 3. Was the exposure accurately measured to minimise bias? 4. Was the outcome accurately measured to minimise bias? 5a. Have the authors identified all-important confounding factors? 5b. Have they taken account of the confounding factors in the design and/or analysis? # If cohort study 6a. Was the follow up of subjects complete enough? # If cohort study: 6b. Was the follow up of subjects long enough?
- **What are the results?**
  7. What are the results of this study? 8a. Are the results precise. 8b. How precise are the results? 9. Do you believe the results?
- **Will the results help locally?**
  10. Can the results be applied to the local population?

Case-control studies:

- **Are the results valid?**
  1.Did the study address a clearly focused issue? 2. Did the authors use an appropriate method to answer their question? 2 .Was the (sample/cohort) recruited in an acceptable way? 3. Were the cases recruited in an acceptable way? 4. Were the controls recruited in an acceptable way? 5. Was the exposure accurately measured to minimise bias? 6a. Aside from the experimental intervention, were the groups treated equally? 6b. Have the authors taken account of the potential confounding factors in the design and/or in their analysis?
- **What are the results?**
  7. How large was the treatment effect? 8. How precise was the estimate of the treatment effect? 9. Do you believe the results?
- **Will the results help locally?**
  10. Can the results be applied to the local population?

Notes: There is no overall score for the CASP tool. We used items from the CASP RoB appraisal to assess certainty of evidence in the Grading of Recommendations Assessment, Development and Evaluation (GRADE) - For more details check **Supplement 5**.

Questions related to validity of results: A study is considered at Low ROB if it answered YES to all questions assessing the validity of the results item (questions "Q2, Q4, Q5, Q6 & Q6, Q6a and Q6b), excluding the accuracy of exposure question (Q3), since we only assessed certainty of evidence using GRADE for daily ECU exposure only.

For results (q7- to q9) - Please check tables in **Supplement 3** for more details.

Will results help locally (q10): reflect generalizability and is used in GRADE- check Supplement 5 for more details.

RoB assessments below are for studies with **daily ECU** exposure only. Questions that can be answered through either Yes (Y), No (N), or unclear (U) are reported.

Tables S4.1: Cardiovascular health (n=19)

Table S4.1a: Randomized controlled trials RoB assessment for cardiovascular health

First author (year)	Study critical appraisal assessment items										
	Are the results of the study valid?						What are the results?				Will the results help locally?
	#1	#2	#3	#4	#5	#6	#7a	#7b	#8a	#8b	#9
**Randomized controlled trials (n=5)**											
(Haziza et al., 2020)	Y	U	Y	U	Y	U	N	-	N	-	U
(Ikonomidis et al., 2020)[1]	Y	N	U	U	Y	U	U	-	N	-	N
(Ludicke et al., 2018)[2]	Y	Y	N	N	U	Y	Y	-	N	-	N
(Ludicke et al., 2019)[3]	Y	U	Y	N	U	Y	N	-	Y	-	U
(Pulvers et al., 2020)[4]	Y	Y	Y	N	N	Y	Y	-	Y	-	U

Table S4.1b: Cross-sectional studies RoB assessment for cardiovascular health

First author (year)	Study critical appraisal assessment items										
	Are the results of the study valid?							What are the results?			Will the results help locally?
	#1	#2	#3	#4	#5a	#5b	#7	#8a	#8b	#9	#10
**Cross-sectional studies (n=14)**											
(Aherrera et al., 2020)[5]	Y	U	Y	U	N	N	-	U	-	U	N
(Arastoo et al., 2020)	Y	U	U	Y	Y	N	-	Y	-	U	U
(Alzahrani et al., 2018)	Y	Y	Y	Y	Y	Y	-	Y	-	Y	Y
(Badea et al., 2019)[6]	Y	N	Y	Y	N	N	-	N	-	Y	N
(Farsalinos et al., 2019)	Y	Y	U	Y	N	U	-	Y	-	Y	U
(Fetterman et al., 2020)	Y	U	Y	Y	N	N	-	U	-	U	N
(Haptonstall et al., 2020)	Y	U	Y	Y	Y	Y	-	Y	-	U	N
(Ip et al., 2020)	Y	U	U	U	N	U	-	Y	-	N	N
(Oliveri et al., 2020)[7]	Y	Y	Y	Y	U	U	-	Y	-	Y	U
(Osei et al., 2019)	Y	Y	Y	Y	Y	Y	-	Y	-	Y	Y
(Podzolkov et al., 2020)	Y	Y	U	Y	Y	Y	-	Y	-	Y	Y
(Rodu & Plurphanswat, 2020)	Y	Y	Y	Y	N	N	-	Y	-	Y	Y
(Sakaguchi et al., 2021)[8]	Y	U	Y	Y	N	N	-	Y	-	U	N
(Vindhyal et al., 2020)	Y	Y	U	U	N	N	-	U	-	Y	Y

[1] Ikonomidis (2020) : Also check immune health
[2] Ludicke 2018: Also check immune and respiratory health
[3] Ludicke 2019: Also check respiratory health
[4] Pulvers (2020): Also check cardiovascular health
[5] Aherrera 2020 : Also check immune health
[6] Badea 2019 : Also check immune health
[7] Oliveri (2020) : Also check immune health
[8] Sakaguchi (2021) : Also check immune and respiratory health

Table S4.2: Immune health (n=33)

Table S4.2a: Randomized controlled trials RoB assessment for immune health

First author (year)	Study critical appraisal assessment items										
	Are the results of the study valid?						What are the results?				Will the results help locally?
	#1	#2	#3	#4	#5	#6	#7a	#7b	#8a	#8b	#9
**Randomized controlled trials** (n=6)											
(Haziza et al., 2020)[9]	Y	U	Y	U	Y	U	N	-	N	-	U
(Ikonomidis et al., 2020)[10]	Y	N	U	U	Y	U	U	-	N	-	N
(Ludicke et al., 2018)[11]	Y	Y	N	N	U	Y	Modest	-	N	-	N
(Ludicke et al., 2019)[12]	Y	U	Y	N	U	Y	Modest	-	Y	-	U
(Song, Reisinger, et al., 2020)	Y	U	Y	N	N	U	N	-	N	-	N
(Rebuli et al., 2021)	Y	N	Y	N	N	Y	U	-	U	-	U

Table S4.2b: Case-control studies RoB assessment for immune health

First author (year)	Study critical appraisal assessment items									
	Are the results of the study valid?						What are the results?		Will the results help locally?	
	#1	#2	#3	#4	#5	#6a	#6b	#7	#8	#9	#10 / (#9 RCT)
**Case-control (n=2)**											
(Ibraheem et al., 2020)[13]	Y	Y	U	Y	Y	Y	N	-	Y	Y	N
Karaaslan[14] (2020)	Y	Y	U	Y	N	Y	N	-	Y	Y	N

Table S4.2c: Cross-sectional studies RoB assessment for immune health

First author (year)	Study critical appraisal assessment items										
	Are the results of the study valid?						What are the results?				Will the results help locally?
	#1	#2	#3	#4	#5a	#5b	#7	#8a	#8b	#9	#10
**Cross-sectional (n=25)**											
(Al Deeb et al., 2020)[15]	Y	U	Y	Y	N	N	-	U	-	U	N

[9] Haziza (2020): All immune health outcomes were measured in context of cardiovascular health. Also see cardiovascular health-

[10] Ikonomidis (2020): Also check cardiovascular health.

[11] Ludicke (2018): All immune health outcomes were measured in context of cardiovascular health. Also see in respiratory, and cardiovascular health.

[12] Ludicke (2019): All immune health outcomes were measured in context of cardiovascular health. Also check in respiratory health

[13] Ibraheem et al. (2020): Also check immune health.

[14] Karaaslan (2020): Also see in oral health.

[15] Al Deeb (2020): All immune health outcomes were collected from peri-implant sulcular fluid (PISF) for periodontal outcomes. Also check oral health.

First author (year)	Study critical appraisal assessment items										
	Are the results of the study valid?						What are the results?				Will the results help locally?
	#1	#2	#3	#4	#5a	#5b	#7	#8a	#8b	#9	#10
**Cross-sectional (n=25)**											
(Al-Aali et al., 2018)	Y	N	Y	Y	N	N	-	U	-	N	N
(Al-Hamoudi et al., 2020)	Y	N	Y	Y	N	N	-	Y	-	U	N
(AlQahtani et al., 2018)[16]	Y	U	Y	Y	N	N	-	N	-	Y	N
(F. Alqahtani et al., 2019)	Y	N	Y	U	N	N	-	Y	-	U	N
(S. Alqahtani et al., 2020)[17]	Y	U	Y	Y	N	N	-	U	-	Y	N
(ArRejaie, 2019)[18]	Y	N	Y	Y	N	N	-	U	-	U	N
(Badea et al., 2019)[19]	Y	N	Y	Y	N	N	-	N	-	Y	N
(BinShabaib et al., 2019)[20]	Y	U	Y	Y	Y	N	-	U	-	U	N
(Cichonska et al., 2019)[21]	Y	N	Y	U	N	N	-	N	-	U	N
(Ganesan et al., 2020)[22]	Y	U	Y	Y	N	N	-	U	-	Y	N
(Gavrilin et al., 2020)	Y	U	Y	Y	U	U	-	U	-	U	N
(Ghosh et al., 2019)	Y	U	Y	Y	N	N	-	U	-	Y	U
(Jackson et al., 2020)	N	U	U	Y	N	N	-	N	-	U	N
(Kelesidis et al., 2020)	Y	U	Y	Y	N	N	-	U	-	N	N
(Lee et al., 2020)	Y	U	Y	Y	N	N	-	U	-	Y	N
(Mokeem et al., 2018)[23]	Y	U	Y	Y	Y	N	-	U	-	U	N
(Oliveri et al., 2020)[24]	Y	Y	Y	Y	U	U	-	N	-	U	N
(Perez et al., 2020)[25]	Y	N	Y	Y	N	N	-	N	-	Y	N
(Pushalkar et al., 2020)[26]	Y	U	Y	Y	N	N	-	U	-	N	N
(Reidel et al., 2018)[27]	Y	U	Y	Y	N	N	-	U	-	U	U
(Sakaguchi et al., 2021)[28]	Y	U	Y	Y	N	N	-	Y	-	U	N
(Shields et al., 2020)	Y	Y	Y	Y	N	N	-	Y	-	Y	Y
(Sinha et al., 2021)[29]	Y	U	Y	Y	N	N	-	U	-	U	N
(Song, Freudenheim, et al., 2020)[30]	Y	N	Y	Y	N	N	-	N	-	Y	N

[16] AlQahtani (2018): Immune indicators were measured as indicators of oral health. Also check oral health

[17] AlQahtani (2020): Immune indicators were measured as indicators of oral health.
[18] ArRejaie (2019): Immune indicators were measured as indicators of peri-implants clinical assessments. Also check oral health.
[19] Badea (2019): Also check cardiovascular health
[20] BinShabaib (2019): Immune indicators were measured as indicators of oral health. Also check oral health.
[21] Cichonska (2019): Immune indicators were measured from saliva samples for oral antibacterial health outcomes.
[22] Ganesan (2021): Immune indicators were measured as indicators of oral health. Also check oral Health.
[23] Mokeem (2018): All immune indicators measured as indicators of oral health. Also check oral Health
[24] Oliveri (2020): Also check cardiovascular health.
[25] Perez (2021): All immune indicators were measured via sputum and serum as a biomarker of pulmonary health. Also check respiratory health.
[26] Pushalkar (2020): Immune indicators were measured as indicators of oral health.
[27] Reidel (2018): All indicators were collected via sputum and measured as an indicator of respiratory/immune health
[28] Sakaguchi (2021): Also check respiratory and cardiovascular health
[29] Sinha (2021): Also check oral health
[30] Song (2020): All immune indicators were measured as an indicator of respiratory health/injury/inflammation

Table S4.3: Oral health (n=19)

Table S4.3a: Cohort studies RoB assessment for oral health

First author (year)	Study critical appraisal assessment items												
	Are the results of the study valid?								What are the results?				Will the results help locally?
	#1	#2	#3	#4	#5a	#5b	#6a	#6b	#7	#8a	#8b	#9	#10
**Cohort studies (n=2)**													
(ALHarthi et al., 2019)	Y	N	Y	Y	N	N	Y	Y	-	U	-	U	N
(Atuegwu et al., 2019)	Y	Y	U	N	N	N	Y	Y	-	Y	-	Y	Y

Table S4.3b: Case-control studies RoB assessment for oral health

First author (year)	Study critical appraisal assessment items										
	Are the results of the study valid?							What are the results?			Will the results help locally?
	#1	#2	#3	#4	#5a	#6a	#6b	#7	#8	#9	#10
**Case-control studies (n=2)**											
(Ibraheem et al., 2020)	Y	Y	U	U	Y	Y	N	-	Y	Y	N
(Karaaslan et al., 2020)[31]	Y	Y	U	U	N	Y	N	-	Y	U	N

Table S4.3c: Cross-sectional studies RoB assessment for oral health

First author (year)	Study critical appraisal assessment items										
	Are the results of the study valid?						What are the results				Will the results help locally?
	#1	#2	#3	#4	#5a	#5b	#7	#8a	#8b	#9	#10
**Cross sectional studies (n=15)**											
(Aherrera et al., 2020)[32]	Y	U	Y	U	N	N	-	U	-	U	N
(Al Deeb et al., 2020)[33]	Y	U	Y	Y	N	N	-	U	-	U	N
(Al-Aali et al., 2018)[34]	Y	N	Y	Y	N	N	-	U	-	N	N
(Aldakheel et al., 2020)	Y	U	Y	Y	N	N	-	Y	-	U	N
(Al-Hamoudi et al., 2020)[35]	Y	N	Y	Y	N	N	-	Y	-	U	N
(AlQahtani et al., 2018)[36]	Y	U	Y	Y	N	N	-	N	-	Y	N
(F. Alqahtani et al., 2019)[37]	Y	N	Y	U	N	N	-	Y	-	U	N

[31] Karaaslan (2020) : Also check immune health
[32] Aherrera (2020): Also see in respiratory, cardiovascular, and immune health
[33] Al Deeb (2020): Also check immune health
[34] Al-Aali 2018: Also check immune health
[35] Al-Hamoudi (2020): Also check immune health
[36] AlQahtani (2018): Also check immune health
[37] AlQahtani (2019): Also check immune health

First author (year)	Study critical appraisal assessment items									
	Are the results of the study valid?						What are the results			Will the results help locally?
	#1	#2	#3	#4	#5a	#5b	#7	#8a	#8b	#9	#10
(ArRejaie, 2019)[38]	Y	N	Y	Y	N	N	-	U	-	U	N
(BinShabaib et al., 2019)[39]	Y	U	Y	Y	Y	N	-	U	-	U	N
(Huilgol et al., 2019)	Y	Y	Y	N	Y	Y	-	Y	-	Y	Y
(Javed et al., 2017)	Y	N	Y	Y	N	N	-	Y	-	N	N
(Mokeem et al., 2018)	Y	U	Y	Y	Y	N	-	U	-	U	N
(Mokeem et al., 2019)	Y	N	Y	Y	Y	N	-	U	-	U	N
(Sinha et al., 2021)[40]	Y	U	Y	Y	N	N	-	U	-	U	N
(Vohra et al., 2020)	Y	N	Y	Y	N	N	-	U	-	N	N

Table S4.4: Respiratory health (n=15)

Table S4.4a: Randomized controlled trials RoB assessment for respiratory health

First author (year)	Study critical appraisal assessment items										
	Are the results of the study valid?						What are the results?				Will the results help locally?
	#1	#2	#3	#4	#5	#6	#7a	#7b	#8a	#8b	#9
**Randomized controlled trials (n=4)**											
(Haziza et al., 2020)[41]	Y	U	Y	U	N	U	N	-	N	-	U
(Ludicke et al., 2018)[42]	Y	Y	N	N	U	Y	Modest	-	N	-	N
(Ludicke et al., 2019)[43]	Y	U	Y	N	U	Y	Modest	-	Y	-	Y
(Pulvers et al., 2020)[44]	Y	Y	Y	N	N	Y	Y	-	Y	-	U

Table S4.4b: Cohort studies RoB assessment for respiratory health

First author (year)	Study critical appraisal assessment items											
	Are the results of the study valid?								What are the results?			Will the results help locally?
	#1	#2	#3	#4	#5a	#5b	#6a	#6b	#7	#8a	#8b	#9	#10
**Cohort studies (n=3)**													
(Polosa et al., 2020)	Y	N	Y	Y	N	N	Y	Y	-	U	-	Y	N
(Polosa et al., 2018)	Y	Y	Y	Y	N	N	Y	Y	-	Y	-	Y	N
(Polosa et al., 2017)	Y	N	Y	Y	N	N	N	Y	-	Y	-	U	N

[38] ArRejaie (2019): Also check immune health
[39] BinShabaib (2019): Also check immune health
[40] Sinha (2021): Also check immune health
[41] Haziza (2020): Also check cardiovascular, and immune health
[42] Ludick (2020): Also check immune health
[43] Ludick (2019): Also check immune and cardiovascular health
[44] Pulvers (2020): Also check cardiovascular health

Table S4.4c: Cross-sectional studies RoB assessment for respiratory health

First author (year)	Study critical appraisal assessment items										
	Are the results of the study valid?						What are the results?				Will the results help locally?
	#1	#2	#3	#4	#5a	#5b	#7	#8a	#8b	#9	#10
**Cross sectional studies (n=8)**											
(AboElNaga, 2018)	Y	Y	Y	Y	N	N	-	U	-	Y	N
(Aherrera et al., 2020)[45]	Y	U	Y	U	N	N	-	U	-	U	N
(Cassidy et al., 2020)	Y	U	Y	Y	N	N	-	Y	-	N	N
(Ghosh et al., 2019)[46]	Y	U	Y	Y	N	N	-	U	-	Y	U
(Kizhakke Puliyakote et al., 2020)	Y	U	Y	U	N	N	-	Y	-	N	N
(Meo et al., 2019)	Y	U	Y	Y	Y	Y	-	Y	-	Y	N
(Perez et al., 2020)[47]	Y	N	Y	Y	N	N	-	N	-	Y	N
(Sakaguchi et al., 2021)[48]	Y	U	Y	Y	N	N	-	Y	-	U	N

References of studies with daily ECU (n=59)

AboElNaga, H. H. (2018). Electronic cigarettes: Not an advantageous alternative to conventional smoking in asthma. *Egyptian Journal of Chest Diseases and Tuberculosis*, *67*(4), 427–432. https://doi.org/http://dx.doi.org/10.4103/ejcdt.ejcdt_83_18

Aherrera, A., Aravindakshan, A., Jarmul, S., Olmedo, P., Chen, R., Cohen, J. E., Navas-Acien, A., & Rule, A. M. (2020). E-cigarette use behaviors and device characteristics of daily exclusive e-cigarette users in Maryland: Implications for product toxicity. *Tobacco Induced Diseases*, *18*(Journal Article PG-93), 93. https://doi.org/https://dx.doi.org/10.18332/tid/128319

Al-Aali, K. ., ArRejaie, A., Abduljabbar, T., Vohra, F., & Akram, Z. (2018). Peri-implant parameters, tumor necrosis factor-alpha, and interleukin-1 beta levels in vaping individuals. *Clinical Implant Dentistry and Related Research*, *20*(3), 410–415. https://doi.org/http://dx.doi.org/10.1111/cid.12597

Al-Hamoudi, N., Alsahhaf, A., Al Deeb, M., Alrabiah, M., Vohra, F., & Abduljabbar, T. (2020). Effect of scaling and root planing on the expression of anti-inflammatory cytokines (IL-4, IL-9, IL-10, and IL-13) in the gingival crevicular fluid of electronic cigarette users and non-smokers with moderate chronic periodontitis. *Journal of Periodontal & Implant Science*, *50*(2 PG-74–82), 74–82. https://doi.org/https://dx.doi.org/10.5051/jpis.2020.50.2.74

Al Deeb, M., Alresayes, S., Mokeem, S. A., Alhenaki, A., AlHelal, A., Shafqat, S., Vohra, F., & Abduljabbar, T. (2020). Clinical and immunological peri-implant parameters among cigarette and electronic smoking patients treated with photochemotherapy: A randomized controlled clinical trial. *Photodiagnosis and Photodynamic Therapy*, *31*(Journal Article), 101800. https://doi.org/http://dx.doi.org/10.1016/j.pdpdt.2020.101800

Aldakheel, F. M., Alduraywish, S. A., Jhugroo, P., Jhugroo, C., & Divakar, D. D. (2020). Quantification of pathogenic bacteria in the subgingival oral biofilm samples collected from cigarette-smokers, individuals using electronic nicotine delivery systems and non-smokers with and without periodontitis. *Archives of Oral Biology*, *117*(Journal

[45] Aherrera (2020): Also check oral, cardiovascular, and immune health
[46] Ghosh A (2019): Also check immune heath
[47] Perez (2021): Also check immune health
[48] Sakaguchi (2021): Also see in immune and cardiovascular health

Article), 104793. https://doi.org/http://dx.doi.org/10.1016/j.archoralbio.2020.104793

ALHarthi, S. S., BinShabaib, M., Akram, Z., Rahman, I., Romanos, G. E., & Javed, F. (2019). Impact of cigarette smoking and vaping on the outcome of full-mouth ultrasonic scaling among patients with gingival inflammation: a prospective study. *Clinical Oral Investigations*, *23*(6 PG-2751–2758), 2751–2758. https://doi.org/https://dx.doi.org/10.1007/s00784-018-2725-2

Alqahtani, F., Alqahtani, M., Albaqawi, A. H., Al-Kheraif, A., & Javed, F. (2019). Comparison of cotinine levels in the peri-implant sulcular fluid among cigarette and waterpipe smokers, electronic-cigarette users, and nonsmokers. *Clinical Implant Dentistry and Related Research*, *21*(4 PG-702–707), 702–707. https://doi.org/https://dx.doi.org/10.1111/cid.12813

AlQahtani, M. A., Alayad, A. S., Alshihri, A., Correa, F. O. B., & Akram, Z. (2018). Clinical peri-implant parameters and inflammatory cytokine profile among smokers of cigarette, e-cigarette, and waterpipe. *Clinical Implant Dentistry and Related Research*, *20*(6), 1016–1021. https://doi.org/http://dx.doi.org/10.1111/cid.12664

Alqahtani, S., Cooper, B., Spears, C. A., Wright, C., & Shannahan, J. (2020). Electronic nicotine delivery system-induced alterations in oral health via saliva assessment. *Experimental Biology and Medicine*, *245*(15), 1319–1325. https://doi.org/http://dx.doi.org/10.1177/1535370220941258

Alzahrani, T., Pena, I., Temesgen, N., & Glantz, S. A. (2018). Association Between Electronic Cigarette Use and Myocardial Infarction. *American Journal of Preventive Medicine*, *55*(4), 455–461. https://doi.org/http://dx.doi.org/10.1016/j.amepre.2018.05.004

Arastoo, S., Haptonstall, K. P., Choroomi, Y., Moheimani, R., Nguyen, K., Tran, E., Gornbein, J., & Middlekauff, H. R. (2020). Acute and chronic sympathomimetic effects of e-cigarette and tobacco cigarette smoking: Role of nicotine and non-nicotine constituents. *American Journal of Physiology - Heart and Circulatory Physiology*, *319*(2), H262–H270. https://doi.org/http://dx.doi.org/10.1152/ajpheart.00192.2020

ArRejaie, A. S. (2019). Proinflammatory cytokine levels and peri-implant parameters among cigarette smokers, individuals vaping electronic cigarettes, and non-smokers. *Journal of Periodontology*, *90*(4), 367–374. https://doi.org/http://dx.doi.org/10.1002/JPER.18-0045

Atuegwu, N. C., Perez, M. F., Oncken, C., Thacker, S., Mead, E. L., & Mortensen, E. M. (2019). Association between regular electronic nicotine product use and self-reported periodontal disease status: Population assessment of tobacco and health survey. *International Journal of Environmental Research and Public Health*, *16*(7), 1263. https://doi.org/http://dx.doi.org/10.3390/ijerph16071263

Badea, M., Gaman, L., Delia, C., Ilea, A., Leasu, F., Henriquez-Hernandez, L. A., Luzardo, O. P., Radoi, M., & Rogozea, L. (2019). Trends of lipophilic, antioxidant and hematological parameters associated with conventional and electronic smoking habits in middle-age Romanians. *Journal of Clinical Medicine*, *8*(5), 665. https://doi.org/http://dx.doi.org/10.3390/jcm8050665

BinShabaib, M., ALHarthi, S. S., Akram, Z., Khan, J., Rahman, I., Romanos, G. E., & Javed, F. (2019). Clinical periodontal status and gingival crevicular fluid cytokine profile among cigarette-smokers, electronic-cigarette users and never-smokers. *Archives of Oral Biology*, *102*(Journal Article PG-212-217), 212–217. https://doi.org/https://dx.doi.org/10.1016/j.archoralbio.2019.05.001

Cichonska, D., Kusiak, A., Kochanska, B., Ochocinska, J., & Swietlik, D. (2019). Influence of electronic cigarettes on selected antibacterial properties of saliva. *International Journal of Environmental Research and Public Health*, *16*(22), 4433. https://doi.org/http://dx.doi.org/10.3390/ijerph16224433

Farsalinos, K. E., Polosa, R., Cibella, F., & Niaura, R. (2019). Is e-cigarette use associated with coronary heart disease and myocardial infarction? Insights from the 2016 and 2017 National Health Interview Surveys. *Therapeutic Advances in Chronic Disease*, *10*(Journal Article). https://doi.org/http://dx.doi.org/10.1177/2040622319877741

Fetterman, J. L., Keith, R. J., Palmisano, J. N., McGlasson, K. L., Weisbrod, R. M., Majid, S., Bastin, R., Stathos, M. M., Stokes, A. C., Robertson, R. M., Bhatnagar, A., & Hamburg, N. M. (2020). Alterations in Vascular Function Associated With the Use of Combustible and Electronic Cigarettes. *Journal of the American Heart Association*, *9*(9),

e014570. https://doi.org/http://dx.doi.org/10.1161/JAHA.119.014570

Ganesan, S. M., Dabdoub, S. M., Nagaraja, H. N., Scott, M. L., Pamulapati, S., Berman, M. L., Shields, P. G., Wewers, M. E., & Kumar, P. S. (2020). Adverse effects of electronic cigarettes on the disease-naive oral microbiome. *Science Advances*, *6*(22). https://doi.org/http://dx.doi.org/10.1126/sciadv.aaz0108

Gavrilin, M. A., McAndrew, C. C., Prather, E. R., Tsai, M., Spitzer, C. R., Song, M. A., Mitra, S., Sarkar, A., Shields, P. G., Diaz, P. T., & Wewers, M. D. (2020). Inflammasome Adaptor ASC Is Highly Elevated in Lung Over Plasma and Relates to Inflammation and Lung Diffusion in the Absence of Speck Formation. *Frontiers in Immunology*, *11*(Journal Article), 461. https://doi.org/http://dx.doi.org/10.3389/fimmu.2020.00461

Ghosh, A., Coakley, R. D., Ghio, A. J., Muhlebach, M. S., Esther, C. R., Alexis, N. E., & Tarran, R. (2019). Chronic E-Cigarette Use Increases Neutrophil Elastase and Matrix Metalloprotease Levels in the Lung. *American Journal of Respiratory and Critical Care Medicine*, *Journal Article*. https://doi.org/http://dx.doi.org/10.1164/rccm.201903-0615OC

Haptonstall, K. P., Choroomi, Y., Moheimani, R., Nguyen, K., Tran, E., Lakhani, K., Ruedisueli, I., Gornbein, J., & Middlekauff, H. R. (2020). Differential effects of tobacco cigarettes and electronic cigarettes on endothelial function in healthy young people. *American Journal of Physiology - Heart and Circulatory Physiology*, *319*(3), H547–H556. https://doi.org/http://dx.doi.org/10.1152/ajpheart.00307.2020

Haziza, C., Bourdonnaye, de La, Donelli, A., Skiada, D., Poux, V., Weitkunat, R., Baker, G., Picavet, P., & Ludicke, F. (2020). Favorable Changes in Biomarkers of Potential Harm to Reduce the Adverse Health Effects of Smoking in Smokers Switching to the Menthol Tobacco Heating System 2.2 for Three Months (Part 2). *Nicotine & Tobacco Research : Official Journal of the Society for Research on Nicotine and Tobacco*, *Journal Article*. https://doi.org/http://dx.doi.org/10.1093/ntr/ntz084

Huilgol, P., Bhatt, S. P., Biligowda, N., Wright, N. C., & Wells, J. M. (2019). Association of e-cigarette use with oral health: a population-based cross-sectional questionnaire study. *Journal of Public Health (Oxford, England)*, *41*(2), 354–361. https://doi.org/http://dx.doi.org/10.1093/pubmed/fdy082

Ibraheem, W. I., Fageeh, H. I., Preethanath, R. S., Alzahrani, F. A., Al-Zawawi, A. S., Divakar, D. D., & Al-Kheraif, A. A. (2020). Comparison of RANKL and osteoprotegerin levels in the gingival crevicular fluid of young cigarette- and waterpipe-smokers and individuals using electronic nicotine delivery systems. *Archives of Oral Biology*, *115*(Journal Article), 104714. https://doi.org/http://dx.doi.org/10.1016/j.archoralbio.2020.104714

Ikonomidis, I., Katogiannis, K., Kostelli, G., Kourea, K., Kyriakou, E., Kypraiou, A., Tsoumani, M., Andreadou, I., Lambadiari, V., Plotas, P., Thymis, I., & Tsantes, A. E. (2020). Effects of electronic cigarette on platelet and vascular function after four months of use. *Food and Chemical Toxicology*, *141*(Journal Article), 111389. https://doi.org/http://dx.doi.org/10.1016/j.fct.2020.111389

Ip, M., Diamantakos, E., Haptonstall, K., Choroomi, Y., Moheimani, R. S., Nguyen, K. H., Tran, E., Gornbein, J., & Middlekauff, H. R. (2020). Tobacco and electronic cigarettes adversely impact ECG indexes of ventricular repolarization: implication for sudden death risk. *American Journal of Physiology.Heart and Circulatory Physiology*, *318*(5), H1176–H1184. https://doi.org/http://dx.doi.org/10.1152/ajpheart.00738.2019

Jackson, M., Singh, K. P., Lamb, T., McIntosh, S., & Rahman, I. (2020). Flavor preference and systemic immunoglobulin responses in e-cigarette users and waterpipe and tobacco smokers: A pilot study. *International Journal of Environmental Research and Public Health*, *17*(2), 640. https://doi.org/http://dx.doi.org/10.3390/ijerph17020640

Javed, F., Abduljabbar, T., Vohra, F., Malmstrom, H., Rahman, I., & Romanos, G. E. (2017). Comparison of Periodontal Parameters and Self-Perceived Oral Symptoms Among Cigarette Smokers, Individuals Vaping Electronic Cigarettes, and Never-Smokers. *Journal of Periodontology*, *88*(10), 1059–1065. https://doi.org/http://dx.doi.org/10.1902/jop.2017.170197

Karaaslan, F., Dikilitas, A., & Yigit, U. (2020). The effects of vaping electronic cigarettes on periodontitis. *Australian Dental Journal*, *65*(2), 143–149. https://doi.org/http://dx.doi.org/10.1111/adj.12747

Kelesidis, T., Tran, E., Arastoo, S., Lakhani, K., Heymans, R., Gornbein, J., & Middlekauff, H. R. (2020). Elevated

Cellular Oxidative Stress in Circulating Immune Cells in Otherwise Healthy Young People Who Use Electronic Cigarettes in a Cross-Sectional Single-Center Study: Implications for Future Cardiovascular Risk. *Journal of the American Heart Association*, *9*(18), e016983. https://doi.org/http://dx.doi.org/10.1161/JAHA.120.016983

Kizhakke Puliyakote, A. S., Elliott, A. R., Sa, R. C., Anderson, K. M., Crotty Alexander, L. E., & Hopkins, S. R. (2020). Vaping Disrupts Ventilation-Perfusion Matching in Asymptomatic Users. *Journal of Applied Physiology (Bethesda, Md.: 1985)*, *Journal Article*. https://doi.org/http://dx.doi.org/10.1152/japplphysiol.00709.2020

Lee, A. C., Chakladar, J., Li, W. T., Chen, C., Chang, E. Y., Wang-Rodriguez, J., & Ongkeko, W. M. (2020). Tobacco, but not nicotine and flavor-less electronic cigarettes, induces ace2 and immune dysregulation. *International Journal of Molecular Sciences*, *21*(15), 1–16. https://doi.org/http://dx.doi.org/10.3390/ijms21155513

Ludicke, F., Ansari, S. M., Lama, N., Blanc, N., Bosilkovska, M., Donelli, A., Picavet, P., Baker, G., Haziza, C., Peitsch, M., & Weitkunat, R. (2019). Effects of Switching to a Heat-Not-Burn Tobacco Product on Biologically-Relevant Biomarkers to assess a Candidate Modified Risk Tobacco Product: A Randomized Trial. *Cancer Epidemiology, Biomarkers & Prevention : A Publication of the American Association for Cancer Research, Cosponsored by the American Society of Preventive Oncology*, *Journal Article*. https://doi.org/http://dx.doi.org/10.1158/1055-9965.EPI-18-0915

Ludicke, F., Picavet, P., Baker, G., Haziza, C., Poux, V., Lama, N., & Weitkunat, R. (2018). Effects of switching to the menthol tobacco heating system 2.2, smoking abstinence, or continued cigarette smoking on clinically relevant risk markers: A randomized, controlled, open-label, multicenter study in sequential confinement and ambulatory settin. *Nicotine and Tobacco Research*, *20*(2), 173–182. https://doi.org/http://dx.doi.org/10.1093/ntr/ntx028

Meo, S. A., Ansary, M. A., Barayan, F. R., Almusallam, A. S., Almehaid, A. M., Alarifi, N. S., Alsohaibani, T. A., & Zia, I. (2019). Electronic Cigarettes: Impact on Lung Function and Fractional Exhaled Nitric Oxide Among Healthy Adults. *American Journal of Men's Health*, *13*(1), 1557988318806073. https://doi.org/http://dx.doi.org/10.1177/1557988318806073

Mokeem, S. A., Abduljabbar, T., Al-Kheraif, A. A., Alasqah, M. N., Michelogiannakis, D., Samaranayake, L. P., & Javed, F. (2019). Oral Candida carriage among cigarette- and waterpipe-smokers, and electronic cigarette users. *Oral Diseases*, *25*(1), 319–326. https://doi.org/http://dx.doi.org/10.1111/odi.12902

Mokeem, S. A., Alasqah, M. N., Michelogiannakis, D., Al-Kheraif, A. A., Romanos, G. E., & Javed, F. (2018). Clinical and radiographic periodontal status and whole salivary cotinine, IL-1beta and IL-6 levels in cigarette- and waterpipe-smokers and E-cig users. *Environmental Toxicology and Pharmacology*, *61*(Journal Article), 38–43. https://doi.org/http://dx.doi.org/10.1016/j.etap.2018.05.016

Oliveri, D., Liang, Q., & Sarkar, M. (2020). Real-World Evidence of Differences in Biomarkers of Exposure to Select Harmful and Potentially Harmful Constituents and Biomarkers of Potential Harm Between Adult E-Vapor Users and Adult Cigarette Smokers. *Nicotine & Tobacco Research : Official Journal of the Society for Research on Nicotine and Tobacco*, *22*(7 PG-1114–1122), 1114–1122. https://doi.org/https://dx.doi.org/10.1093/ntr/ntz185

Osei, A. D., Mirbolouk, M., Orimoloye, O. A., Dzaye, O., Uddin, S. M. I., Benjamin, E. J., Hall, M. E., DeFilippis, A. P., Stokes, A., Bhatnagar, A., Nasir, K., & Blaha, M. J. (2019). Association Between E-Cigarette Use and Cardiovascular Disease Among Never and Current Combustible-Cigarette Smokers. *American Journal of Medicine*, *132*(8), 949. https://doi.org/http://dx.doi.org/10.1016/j.amjmed.2019.02.016

Perez, M. F., Atuegwu, N. C., Mortensen, E. M., & Oncken, C. (2020). The inflammatory biomarker YKL-40 is elevated in the serum, but not the sputum, of E-cigarette users. *Experimental Lung Research*, *Journal Article*. https://doi.org/http://dx.doi.org/10.1080/01902148.2020.1847216

Podzolkov, V. I., Bragina, A. E., Druzhinina, N. A., Vasil'eva, L. V, Osadchiy, K. K., Dubchak, A. E., & Khvalin, E. I. (2020). Relation between Tobacco Smoking/Electronic Smoking and Albuminuria/Vascular Stiffness in Young People without Cardiovascular Diseases. *Kidney and Blood Pressure Research*, *45*(3), 467–476. https://doi.org/http://dx.doi.org/10.1159/000507510

Polosa, R., Cibella, F., Caponnetto, P., Maglia, M., Prosperini, U., Russo, C., & Tashkin, D. (2017). Health impact of E-cigarettes: a prospective 3.5-year study of regular daily users who have never smoked. *Scientific Reports*, *7*(1),

13825. https://doi.org/http://dx.doi.org/10.1038/s41598-017-14043-2

Polosa, R., Morjaria, J. B., Prosperini, U., Busa, B., Pennisi, A., Malerba, M., Maglia, M., & Caponnetto, P. (2020). COPD smokers who switched to e-cigarettes: health outcomes at 5-year follow up. *Therapeutic Advances in Chronic Disease*, *11*(Journal Article). https://doi.org/http://dx.doi.org/10.1177/2040622320961617

Polosa, R., Morjaria, J. B., Prosperini, U., Russo, C., Pennisi, A., Puleo, R., Caruso, M., & Caponnetto, P. (2018). Health effects in COPD smokers who switch to electronic cigarettes: A retrospective-prospective 3-year follow-up. *International Journal of COPD*, *13*(Journal Article), 2533–2542. https://doi.org/http://dx.doi.org/10.2147/COPD.S161138

Pulvers, K., Nollen, N. L., Rice, M., Schmid, C. H., Qu, K., Benowitz, N. L., & Ahluwalia, J. S. (2020). Effect of Pod e-Cigarettes vs Cigarettes on Carcinogen Exposure among African American and Latinx Smokers: A Randomized Clinical Trial. *JAMA Network Open*, *3*(11), 26324. https://doi.org/http://dx.doi.org/10.1001/jamanetworkopen.2020.26324

Pushalkar, S., Paul, B., Li, Q., Yang, J., Vasconcelos, R., Makwana, S., Gonzalez, J. M., Shah, S., Xie, C., Janal, M. N., Queiroz, E., Bederoff, M., Leinwand, J., Solarewicz, J., Xu, F., Aboseria, E., Guo, Y., Aguallo, D., Gomez, C., … Saxena, D. (2020). Electronic Cigarette Aerosol Modulates the Oral Microbiome and Increases Risk of Infection. *IScience*, *23*(3 PG-100884), 100884. https://doi.org/https://dx.doi.org/10.1016/j.isci.2020.100884

Rebuli, M. E., Glista-Baker, E., Hoffman, J. R., Duffney, P. F., Robinette, C., Speen, A. M., Pawlak, E. A., Dhingra, R., Noah, T. L., & Jaspers, I. (2021). Electronic-cigarette use alters nasal mucosal immune response to live-attenuated influenza virus: A clinical trial. *American Journal of Respiratory Cell and Molecular Biology*, *64*(1), 126–137. https://doi.org/http://dx.doi.org/10.1165/rcmb.2020-0164OC

Reidel, B., Radicioni, G., Clapp, P. W., Ford, A. A., Abdelwahab, S., Rebuli, M. E., Haridass, P., Alexis, N. E., Jaspers, I., & Kesimer, M. (2018). E-cigarette use causes a unique innate immune response in the lung, involving increased neutrophilic activation and altered mucin secretion. *American Journal of Respiratory and Critical Care Medicine*, *197*(4), 492–501. https://doi.org/http://dx.doi.org/10.1164/rccm.201708-1590OC

Rodu, B., & Plurphanswat, N. (2020). A re-analysis of e-cigarette use and heart attacks in PATH wave 1 data. *Addiction (Abingdon, England)*, *115*(11), 2176–2179. https://doi.org/http://dx.doi.org/10.1111/add.15067

Sakaguchi, C., Nagata, Y., Kikuchi, A., Takeshige, Y., & Minami, N. (2021). Differences in Levels of Biomarkers of Potential Harm among Users of a Heat-not-burn Tobacco Product, Cigarette Smokers, and Never-Smokers in Japan: A Post-Marketing Observational Study. *Nicotine & Tobacco Research : Official Journal of the Society for Research on Nicotine and Tobacco*, *Journal Article PG-*. https://doi.org/https://dx.doi.org/10.1093/ntr/ntab014

Shields, P. G., Song, M. A., Freudenheim, J. L., Brasky, T. M., McElroy, J. P., Reisinger, S. A., Weng, D. Y., Ren, R., Eissenberg, T., Wewers, M. D., & Shilo, K. (2020). Lipid laden macrophages and electronic cigarettes in healthy adults. *EBioMedicine*, *60*(Journal Article), 102982. https://doi.org/http://dx.doi.org/10.1016/j.ebiom.2020.102982

Sinha, D. K., Vishal, Kumar, A., Khan, M., Kumari, R., & Kesari, M. (2021). Evaluation of tumor necrosis factor-alpha (TNF-alpha) and interleukin (IL)-1beta levels among subjects vaping e-cigarettes and nonsmokers. *Journal of Family Medicine and Primary Care*, *9*(2 PG-1072–1075), 1072–1075. https://doi.org/https://dx.doi.org/10.4103/jfmpc.jfmpc_902_19

Song, M. A., Freudenheim, J. L., Brasky, T. M., Mathe, E. A., McElroy, J. P., Nickerson, Q. A., Reisinger, S. A., Smiraglia, D. J., Weng, D. Y., Ying, K. L., Wewers, M. D., & Shields, P. G. (2020). Biomarkers of exposure and effect in the lungs of smokers, nonsmokers, and electronic cigarette users A C. *Cancer Epidemiology Biomarkers and Prevention*, *29*(2), 443–451. https://doi.org/http://dx.doi.org/10.1158/1055-9965.EPI-19-1245

Song, M. A., Reisinger, S. A., Freudenheim, J. L., Brasky, T. M., Mathe, E. A., McElroy, J. P., Nickerson, Q. A., Weng, D. Y., Wewers, M. D., & Shields, P. G. (2020). Effects of electronic cigarette constituents on the human lung: A pilot clinical trial. *Cancer Prevention Research*, *13*(2), 145–151. https://doi.org/http://dx.doi.org/10.1158/1940-6207.CAPR-19-0400

Vindhyal, M. R., Okut, H., Ablah, E., Ndunda, P. M., Kallail, K. J., & Choi, W. S. (2020). Cardiovascular Outcomes

Associated With Adult Electronic Cigarette Use. *Cureus*, *12*(8 PG-e9618), e9618. https://doi.org/https://dx.doi.org/10.7759/cureus.9618

Vohra, F., Bukhari, I. A., Sheikh, S. A., Albaijan, R., & Naseem, M. (2020). Comparison of self-rated oral symptoms and periodontal status among cigarette smokers and individuals using electronic nicotine delivery systems. *Journal of American College Health : J of ACH*, *68*(7), 788–793. https://doi.org/http://dx.doi.org/10.1080/07448481.2019.1709476
