# Supplementary material for: Chronic health effects associated with electronic cigarette use: A systematic review
Source: Front Public Health. 2022 Oct 6;10:959622. doi: 10.3389/fpubh.2022.959622 (PMC9584749; doi:10.3389/fpubh.2022.959622)
Supplement: Supplementary file 5 [file Table_5.pdf]

**Supplement S5:** Summary of decision rules for applying GRADE to assess certainty of evidence on the chronic health effects associated with e-cigarette use (ECU)

- Apply GRADE to assess the certainty of evidence **per health outcome.**
- Since we will assess certainty of evidence per outcome, we will revise the outcome accuracy question in the CASP RoB assessment, to evaluate each outcome, not the study as a whole (item Q4 for observational studies, Q5 for case-controls). For randomized control trials (RCTs), there is no direct question to assess outcomes accuracy in the CASP RoB checklist; we will assess that from the question we have asked in the google Form.
- For each health outcome, certainty of evidence (using GRADE), will be applied only for studies that assess **"daily ECU".** We will not apply GRADE, on studies where e-e-cigarette exposure is occasional or unclear.
- We will assess the certainty of evidence for RCT, quasi-experimental and cohort studies separate from other observational designs (i.e. 2 rows of certainty). This will allow us to see if lower quality designs support findings from higher designs.

**To assess level of certainty please go through these three steps:**

1. **Establish initial level of certainty**
   The design of the studies will indicate the entry point for the level of certainty. Rate certainty of evidence for high and low quality study designs separately (i.e. 1 row per certainty level).
   - Start from "**High Certainty**" If there are **RCT, quasi-experimental, prospective cohort studies** that evaluate the impact of ECU on the health outcome.
   - Start from "**Low Certainty"** If observational studies (cross sectional, retrospective cohort studies, or case-controls)

2. **Consider lowering or raising level of certainty**
   - Lower certainty based on the criteria in **Table 1 below** (Risk of Bias, Inconsistency, Indirectness, Imprecision, and Publication bias). Subtract points when needed
   - Higher (raise) certainty:
     - if there is no reason to lower the certainty of evidence and
     - if one of the three domains that can increase certainty in a body of evidence is noted, which are **large effects**, **dose response gradient**, or **adjusting for plausible confounders** or other **biases**

   Consider rating up the level of certainty, particularly if the reasons to upgrade are noted in the majority of studies. Upgrading (raising the level of certainty according to GRADE Guidelines, can be trough adding 1 to 2 points to the level of certainty final score. (We will add from 1 to 1.5 point, to be more on the conservative side).

3. **Final score for the level of certainty**
   After going through Table 1, add the discounted points, half-points are combined across domains to yield a total score (from 0 to -2.5 or more); if the final scoring included a half-point, we conservatively round it up (i.e. discount more)

   - If the initial level of certainty was high and there was no reason to lower the certainty of evidence then the level of certainty will remain **high.**

**High level of certainty** means that we are confident that the true direction of association between ECU and the health outcome lies close to the association that we have estimated and further research is very unlikely to change our confidence in the effect.

- If the initial level of certainty was high, but was discounted by 0.5 or 1 point for any reason based on Table 1, or the initial level of certainty was low, but was eligible for an upgrade (higher the level of certainty) then the final rating will be **moderate level of certainty**.
- If the initial level of certainty was high, but was discounted by 1.5 to 2 points, or the initial level of certainty was low and there was no reason to downgrade it, then the final rating will be **low level of certainty**.

- If the initial level of certainty was high, but was discounted by 2.5 points or more, or the initial level of certainty was low and was discounted by 0.5 points or more, then the final rating will be **very low level of certainty.**

For certainty of evidence from moderate to very low, include an interpretation for the reason you discounted the points for, discuss the limitation in the evidence that made you downgrade the certainty of evidence based on Table 1.

**Table S5-1:** Decision rules to apply GRADE assessments for certainty of evidence on chronic health effects associated with ECU- using the CASP RoB checklist, vote counting table, and potential conflict of interest related to direct or indirect funding.

| Domain | Judgment | Scoring | Criteria to follow to apply GRADE | Tools used in our systematic Review: CASP questions, vote counting tables and publication conflict of interest | Equivalent criteria in GRADE- for reference |
|---|---|---|---|---|---|
| **Risk of Bias** | No serious risk of bias | 0 | All studies were considered at low ROB[1] | **RoB in validity of results:**<br><br>**if RCT**<br><br>1) Were clinically important outcomes considered (Not assessed in CASP))<br><br>2) Were assignment of patients/ participants treatment randomized *(Q2- in RCT RoB tool)* | **In general:** Make a judgement on the risk of bias across studies for an individual outcome. A sensitivity analysis is not possible to determine if the effect changes when studies at high risk of bias are excluded. It is possible to consider the size of a study, its risk of bias and |
| | Low to moderate risk of bias | –0.5 points | > 75% of the studies were considered at low ROB | | |
| | | –1 point | >50% but < 75% of the studies were considered at low RoB. | | |

- [1] **A study is considered at Low ROB if** it answered YES to all questions assessing the **validity of the results in the ROB tool** (questions "Q2, Q4, Q5, Q6 & Q6, Q6a and Q6b, excluding the accuracy of exposure question, since we will only apply GRADE on Regular exposures"

| Domain | Judgment | Scoring | Criteria to follow to apply GRADE | Tools used in our systematic Review: CASP questions, vote counting tables and publication conflict of interest | Equivalent criteria in GRADE- for reference |
|---|---|---|---|---|---|
| | Very serious risk of bias | –1.5 points | *If data synthesized narratively:*<br>• ≥1 study were at high ROB[3], and a study at high ROB *<u>was responsible</u>* for either the highest or lowest prevalence estimate in the range of estimates for the outcome<br><br>*If data synthesized using regression models*<br>• **<u>50%</u>** or more of studies contributing to an outcome were at high ROB | 3) Were all patients who entered the trial accounted for in the conclusion *(Q3- in RCT RoB tool)*<br><br>*4)* Were patients, health workers and study personnel 'blind' to treatment? *(Q4- in RCT RoB tool)*<br><br>Aside from the experimental intervention, were the groups treated equally? *(Q6- in RCT RoB tool)*<br><br>*5)* Were the groups similar at the start of the trial? *(Q5- in RCT RoB tool)*<br><br>Question: Is the outcome measured accurately? (yes, no). Please refer to the google sheets where we extracted this information.<br><br>**If cross sectional or cohort studies (Q2, Q4 to 6)**<br><br>*Q2*: Population recruited in an acceptable way, Q4: Accuracy of the outcome**,** (Q5a, Q5b) : confounders[2] identified and adjusted for and Q6a | the impact it would have on the summarised effect (Murad, 2017)<br><br>RoB in RCT look at<br><br>1) Selective outcomes reported<br><br>2) Lack of allocation concealment<br><br>3) Incomplete accounting of patients and outcome events.<br><br>4) Lack of blinding<br><br>5) Other limitations<br><br><u>which are equivalent to RCT validity of results section in the CASP (Q2 to Q6)</u> **<u>(Purssell, 2019).</u>**<br><br>Use corresponding questions that measure validity of results for the appropriate study design |

- [3] **A study is considered at <u>High RoB</u> if** it answered No to any of the questions assessing the **<u>validity of the results in the ROB tool</u>** ("Q2 to Q6 in RCT, Q2 to Q6 in cross sectional and cohort studies, and Q2, Q4 and Q6 in case control studies, excluding the accuracy of exposure question, since GRADE will be applied only on daily ECU"

- [2] For the question of confounders in the RoB assessment (Q5), individual studies should have <u>at least</u> adjusted for age, gender, SES measures, and exposure to ECU and smoking (through adjusted models using covariates or through stratified models).

| Domain | Judgment | Scoring | Criteria to follow to apply GRADE | Tools used in our systematic **Review:** CASP questions, vote counting tables and publication conflict of interest | Equivalent criteria in GRADE- for reference |
|---|---|---|---|---|---|
| | | | | and Q6b: Follow up of subjects complete and long enough<br><br>**If case control (Q2 to Q4, and Q6)**<br>Q2: Appropriate method used to answer their question, Q4a Q4b: Cases and controls recruited in an acceptable way, Q6: Aside from the experimental intervention, groups are treated equally. Q6b: confounders taken into account. | CASP RoB tool, excluding the exposure to ECU accuracy measure, since we only assessed daily exposures. |
| **Inconsistency** | No serious inconsistency | 0 | Look at the direction of effect.<br><br>• If the judgment of heterogeneity is considered expected or acceptable (≥75% are in the same direction (e.g., positive). | Use the vote counting tables (Table 3 in manuscript) to evaluate consistency of the direction.<br><br>The magnitude of effect is not used in this systematic review. We only consider direction, and statistical significant differences for each outcome comparing ECU to traditional smokers (TS, non-smokers (NS), or dual users (DU). | Judge inconsistency by evaluating the consistency of the direction and primarily the difference in the magnitude of effects across studies (since statistical measures of heterogeneity are not available). Widely differing estimates of the effects indicate inconsistency (Murad, 2017). |
| | Serious inconsistency | –1 point | If only one study contributed to an outcome<br><br>*OR*<br><br>Heterogeneity (if < 75% are in the same direction) | | |
| **Indirectness** | No serious indirectness | 0 | The majority of the studies can be generalizable to the population of interest for our review. **<u>(> 75%</u>** of the studies answered **<u>Yes to Q9</u>** in the RoB (can the results be applied to the local population, or in your context)). | *Can the results be applied to the local population, or in your context?* Q9 in CASP ROB tool for cohort, case-control, and cross sectional studies and Q10 for quasi-experimental studies and RCTs. | Make a global judgement on how dissimilar the research evidence is to the clinical question at hand (in terms of population, interventions and outcomes across studies) (Murad, 2017). |
| | Serious indirectness | –0.5 points | The study/studies contributing to an outcome included only a subset of the target population of interest for our review. (>**<u>25%</u>** or more of the studies answered **<u>No to Q9</u>** in the ROB | | |

| Domain | Judgment | Scoring | Criteria to follow to apply GRADE | Tools used in our systematic Review: CASP questions, vote counting tables and publication conflict of interest | Equivalent criteria in GRADE- for reference |
|---|---|---|---|---|---|
| **Imprecision** | No serious imprecision | 0 | • A threshold of 1250 or more for clinical trails will be used as an OIS threshold, if this OIS is met, do not rate down the imprecision (Guyatt GH, et al, 2011) [4]<br>• For observational studies, we will set an OIS at 10,000, which is equivalent to the minimum sample size for national surveys (Daniel, 2012). | Add the studies sample sizes for each comparison group and make the judgement relying only on the OIS.<br><br>We will not evaluate impression based on confident intervals. We will only reply on the OIS in this item. Because of the wide range of outcomes, it is not feasible to set threshold per outcome. | Consider the optimal information size (OIS) (or the total number of events for binary outcomes and the number of participants in continuous outcomes) across all studies.<br><br>Results may also be imprecise when the CIs of all the studies or of the largest studies include no effect and clinically meaningful benefits or harms (Murad, 2017). |
| | Serious imprecision | -1 points | If OIS is less than 1,250 then rate down for serious imprecision<br>A threshold to evaluate 95% CI imprecision will not be used in this systematic review. We will only evaluate impression based on a fixed OIS across domains. | | |
| **Publication Bias** | No serious risk of publication bias suspected | 0 | • No concerns related to sample size (i.e., with the acceptable range[5]) of positive studies or potential for conflicts of interest relative to funding. | Also consider the funding sources of the studies, if there is a potential conflict of interest, whether declared or not by the authors of each manuscript.<br><br>Conflict of interest is assessed by the extractors (co-authors of this systematic review), and added in Supplement 2 Tables. | Publication bias can be suspected when the body of evidence consists of only small positive studies or when studies are reported in trial registries but not published. Statistical evaluation of publication bias is not possible in this case. Publication bias is more likely if the search of the systematic review is not |
| | Serious risk of publication bias suspected | -1 | • 75% of the studies with positive results papers are small studies:<br>o ≤15 participants per group for experimental research (this includes any clinical study, and RCTs,)<br>o ≤400 participants for community surveys (observational studies), or | | |

[4] If the total number of participants/ patients included in the rapid review < the number of patients generated by a conventional sample size calculation for a single adequately powered trial consider downgrading evidence. We will consider the same OIS threshold across all health domains, assuming $\alpha$ of 0.05, and $\beta$ of 0.2 for relative risk ratio (RRR) of 20%, and the best estimate of control event rate was 0.2, the OIS threshold will be approximately 1, 250 participants (Guyatt GH, et al, 2011).

- [5] Typical sample size for different research designs include: (sample size = 15 to 30 participants per group for experimental research, 400 to 2,500 participants for community surveys, and 10,000 to 15,000 for national surveys (Daniel, 2012).

| Domain | Judgment | Scoring | Criteria to follow to apply GRADE | Tools used in our systematic Review: CASP questions, vote counting tables and publication conflict of interest | Equivalent criteria in GRADE- for reference |
|---|---|---|---|---|---|
| | | | ○ ≤10,000 for national surveys (observational studies).<br><br>OR<br>• 75% of the studies contributing to the positive association of ECU with an outcome have a potential conflict of interest related to funding.<br>OR<br>• Only observational studies are showing positive results (compare the study design to see whether the direction of association is different by study design). Summarize the findings, but donot forget that the final direction of association should come from better-designed studies i.e. RCTs or cohort studies.<br><br>• For studies only assessing prevalence (i.e. injuries), if a study have a potential conflict of interest related to funding, and were responsible for either the highest or lowest prevalence estimate in the range of estimates for the outcome | | comprehensive (Murad, 2017). |

CASP = Critical Appraisal Skills Programme, CI = confidence interval; GRADE = Grading of Recommendations Assessment, Development and Evaluation; OIS = optimal information size; RoB = risk of bias.

## References:


Purssell, E. (2020). Can the Critical Appraisal Skills Programme check-lists be used alongside Grading of Recommendations Assessment, Development and Evaluation to improve transparency and decision-making?. Journal of advanced nursing, 76(4), 1082-1089. DOI: 10.1111/jan.14303

Murad, M. H., Mustafa, R. A., Schünemann, H. J., Sultan, S., & Santesso, N. (2017). Rating the certainty in evidence in the absence of a single estimate of effect. BMJ Evidence-Based Medicine, 22(3), 85-87.

Schünemann, et al. (2013). Introduction to GRADE Handbook https://gdt.gradepro.org/app/handbook/handbook.html#h.xivvyiu1pr3v

Guyatt GH, et al (2011). GRADE guidelines 6. Rating the quality of evidence--imprecision. Journal of clinical epidemiology, 64(12), 1283–1293. https://doi.org/10.1016/j.jclinepi.2011.01.012

Daniel, J. (2012). Choosing the size of the sample. In *Sampling essentials: Practical guidelines for making sampling choices* (pp. 236-253). SAGE Publications, Inc.
