# Supplementary material for: Chronic health effects associated with electronic cigarette use: A systematic review
Source: Front Public Health. 2022 Oct 6;10:959622. doi: 10.3389/fpubh.2022.959622 (PMC9584749; doi:10.3389/fpubh.2022.959622)
Supplement: Supplementary file 6 [file Table_6.pdf]

Supplement S6: Outcome measures

Table S6-1 Summary of outcomes table:

Health domain section	Outcomes	# of papers	Total # of papers
Cardiovascular health	Cardiovascular disease	8	25
	Cardiovascular risk factors	25	
	- Blood pressure	14	
	- Biomarkers for lipid metabolism	8	
	- Cardiovascular function	8	
Immunological health	Inflammation	34	45
	Immune response	22	
	Oxidative stress	15	
Oral health	Periodontal health (Perio)	18	23
	Peri-implant oral health	6	
Respiratory health	Respiratory Symptoms	27	32
	Lung Function	16	
Total			92

Notes: The number of papers are different from the number of studies (e.g., two papers can present findings from 1 study). This was adjusted for in the vote counting Tables, to avoid over counting of outcomes. Also the total number of papers in each domain will add to 124, but they come from 91 papers, so a number of papers examined more than one health outcome.

Outcomes:

Outcome in each health domain is highlighted in "**BLUE** (for e.g. Respiratory Health outcome 1= Res-O1, outcome2 = Res-O2, etc.). The outcome level chosen varies between health domains.

Cardiovascular health (n= 25)

CV-O1: Cardiovascular diseases (n=9)

- Cardiovascular disease/ cardiovascular disease history
- Coronary heart disease/ coronary artery disease
- Myocardial infraction
- Congestive heart failure
- Premature cardiovascular disease
- Peripheral artery disease (PAD)- blocked leg arteries
- Ischemic attack (stroke)

Cardiovascular risk factors (n=21)

- **CV-O2: Blood pressure (n=14)**
    - High blood pressure
    - Systolic blood pressure
    - Diastolic blood pressure
    - Mean blood pressure

- **CV-O3: Biomarkers for lipid metabolism (n=8)**
    - Total cholesterol/ high cholesterol
    - Low HDL-c

    - High LDL-c
    - Triglycerides

- **CV-O4: Cardiovascular function: (n=8)**
    - Vascular function (n=5)
        - Heart rate
        - Brachial-artery diameter
        - Brachial-artery flow mediated dilation (FMD)
        - Velocity reactive hyperemia
        - Shear stress reactive hyperemia

    - Circulation disorders (n=5)
        - Albuminuria level (AU)
        - Augmentation index (ALP)
        - Mean flow velocity
        - Flow-mediated dilation
        - Carotid-radial pulse
        - Mean arterial pressure
        - Arterial elasticity
        - Peak shear rate
        - Artery diameter
        - Arterial stiffness (pulse wave velocity/ peak wave velocity)

Immunological Health: (n=45)

Imm-O1: Inflammation (n=34)

Cytokines and proteins biomarkers of inflammation

- Cytokines which has both pro-inflammatory and anti-inflammatory function:
    - Interleukin IL-6 (pg/μL) : including measured in crevicular fluid cytokine : periodental anti-inflammatory
    - Interleukin IL-11 (pg/μL)
    - Interleukin IL-33 (pg/μL): can function both as a traditional cytokine and as a nuclear factor regulating gene transcription. It is also function as an alarm, released following cell necrosis to alert the immune system to tissue damage or stress

- Pro-inflammatory cytokines:
    - Interleukin IL-2 (pg/μL) : Peri-implant pro-inflammatory cytokines
    - Interleukin IL-6 (pg/μL) : Peri-implant pro-inflammatory cytokines
    - Interleukin IL-8 (pg/μL)
    - InterleukinIL-12 (pg/μL)
    - Interleukin IL-1β : in the crevicular fluid cytokine (periodontal)
    - TGFB –γ ; in the crevicular fluid cytokine (periodontal)
    - IFN-γ / IFN-G (cytokine critical for adaptive immunity against viral infections)
    - INF-y : Peri-implant pro-inflammatory cytokines
    - MMP-8 (pg/ml)
    - CSF: Peri-implant pro-inflammatory cytokines
    - Granulocyte-macrophage colony-simulating factor (GM-CSF)
    - TNF-alpha

- Anti-inflammatory cytokines:
  - Interleukin-1 IL-1RA
  - Interleukin IL-4 (pg/µL) – important in allergic response /periodental anti-inflammatory
  - Interleukin IL-10 (pg/µL)- inhibits cytokines production (IFN-γ, TNF-β, IL-2)/ periodental and peri-implant anti-inflammatory
  - IL-11/ periodental anti-inflammatory
  - Interleukin IL-13 (pg/µL) / periodental anti-inflammatory

- Chemokines**:** direct the migration of white blood cells to infected or damaged tissues
- Non-specific inflammatory proteins:
  - C-reactive protein
  - High-sensitivity C-reactive protein (hs-CRP)- anti-inflammatory

- Antibody
  - IgA: Immunoglobulin A (increases with the Allergy, or other microbial activities)

<u>Other biomarkers of inflammation</u>

- sICAM-1: Soluble intercellular adhesion molecule-1
- GCF volume (µL) : gingival crevicular fluid volume
- PISF
- YKL-40
- Fibrinogen: a glycoprotein complex, during tissue and vascular injury, it is converted enzymatically by thrombin to fibrin and then to a fibrin-based blood clot.

<u>Inflammatory mediators</u>

- Prostaglandins: indicating arachidonic acid metabolites
  - PGE2- : Prostaglandin E2
  - 16-phenoxy tetranor prostaglandin E2 – their elevation suggest periodontal disease 17-phenyl trinor-13 prostaglandin: their elevation suggest periodontal disease
  - 14-dihydro prostaglandin A2: their elevation suggest periodontal disease

- Leukotrienes: (D4 methyl ester and leukotriene E4): inflammatory mediator caused by oxidation of arachidonic acid: related to asthma
- Pentosidine: a marker of advance glycation end products (AGE)- High levels of AGE can cause inflammation and oxidative stress
- Uric acid (high uric acid: hyperuricemia, can cause gout, renal stones, or renal failure)
- CXCL1
- MCP-1
- MIP-1alpha
- MIP-1beta
- RANTES
- Eotaxin
- CXCL2
- Granulocyte colony-stimulating factor (G-CSF)Pycard/ASC

<u>Anti-inflammatory lipid mediators</u>

- EN-RAGE : extracellular newly identified RAGE binding protein
- RAGE : receptor for advanced glycation end products

- MMP-9 : matrix metallopeptidase-9
- S100A8 : S100 calcium-binding protein
- S100A9 : S100 calcium-binding protein
- Galectin-3:
- Uteroglobin/CC-10
- Resolvin D1
- Resolvin D2
- CC16

<u>Factors inhibiting inflammation</u>

- Glutathionyl spermine (inhibits inflammation)
- Gangliosides
- Ceramide
- Angiotensin (angiotensin II, phosphotrosyl-angiotensin II)
  Coenzyme F420 marker of methanogenic Archaea: Its reduction correlates with severity of periodontal disease

Imm-O2: Immune response (n=22)

- Immune infiltration
  - naïve B-cells, memory B-cells, CD8 T-cells, CD4 naïve T-cells, CD4 memory resting T-cells, CD4 memory activated T-cells, follicular helper T-cells, regulatory T-cells, gamma-delta T-cells, resting NK cells, activated NK cells, M0-M2 macrophages, resting dendritic cells, activated dendritic cells, resting mast cells, activated mast cells, monocytes, plasma cells, eosinophils, neutrophils).

- White blood cells
  - Neutrophils
    - Neutrophils counts
    - Neutrophils elastase
    - protein arginine deminase 4
    - Proteinase 3
    -
  - Monocytes
  - Lymphocytes
    - T-cells
    - B-cells
    - NK cells
  - Basophils
  - Eosinophils

- Antimicrobial enzymes
  - Lysozyme : antimicrobial agent (found in tears and saliva)
  - Lactoferrin : transport iron in blood serum - found in milk, (e.g) colostrum
- Protease
  - MMP-9, MMP-2

- Protease inhibitors
  - A1AT, SLPI, TIMP-1, TIMP-2

- Platelet activity
    - Serpine1/PAI-1 : SERPINE1 gene provides instructions for making a protein called plasminogen activator inhibitor -1
    - Tissue plasminogen activator
    - Platelet function: platelet activation by PFA-100
    - Platelet activation (n=4)
        - 11-Dehydro thromboxane B2
        - 2,3-dinor thromboxane B2 (2,3-d-TXB2)

- Growth factors
    - BDNF: brain-derived neurotrophic factor
    - Basic-FGF: fibroblast growth factors
    - Beta-NGF: nerve growth factor
    - SCF: stem cell factor
    - BMP-2: bone morphogenetic protein
    - HGF: hepatocyte growth factor
    - PDGF-AA: platelet-derived growth factor
    - TGF-a : transforming growth factor
    - TGF-β : transforming growth factor
    - EGF: epidermal growth factor
    - PIGF: placenta growth factor
    - VEGF : vascular endothelial growth factor
    - Endothelial growth factor
    - TGC-alpha
    - Basic FGF/FGF2
    - PGF

- Systematic immune response
    - Immunoglobulin G (IgG)
    - Immunoglobulin E (IgE)

- Other protective proteins
    - Mucins: lubricates the epithelial surfaces, and protects from chemical and physical impacts and microbial damage.
        - MUC5AC, MUC4, and MUC5B, MUC5B/MUC5AC ratio
    - Azurocidin: enhance cytokine release.

- Airway epithelial defense protein (immune function/response)

    - dmbt1
    - lactotransferrin
    - trefoil factor 3
    - lysozyme c

- Extracellular matrix breakdown indicator
    - Desmosine: Plasma desmosine is a biomarker of elastin degradation

Imm-O3: Biomarkers of oxidative stress (n=15)

- Salivary malondialdehyde (MDA)/ Malondialdehyde in blood (oxidant)

- Salivary mucins (SM) : responsible for non-immune protection of the oral cavity
- Urinary 8-isoprostane
- Myeloperoxidase (MPO) : peroxidase enzyme
- 8-epi-prostaglandin F2α
- 8 hydroxydeoxyguanosine (8-OHdG)
- Glutathione Peroxidase :(GsH-Px)
- Vitamin A
- Vitamin E
- Albumin
- Antioxidant status by EDEL system
- Metallothinonein
- Resolvin E1
- Aldehyde dehydrogenase 3A1
- Nucleobindin 1
- Thioredoxin
- Glutathion stransferase
- Betamicroseminoprotein

Oral Health (n=23)

OH-O1: Periodontal health (Perio) n= (19)

<u>Hard tissue:</u>

Bones:

- Radiographic periodontal parameters:
  - Total marginal bone loss (MBL)
  - Mesial and distal MBL
- Biomarker of bone loss:
  - Receptor activator of nuclear factor-kB (RANK): osteoprotegerin (OPG): regulates the regulate osteoclasts, a type of bone cell that breaks down bone tissues.

Teeth: (n=3)

- Self-reported teeth issues:
  - Cavities
  - Toothache
  - Sensitive teeth

- Discolouration and staining:
  - Self reported dental discolouration/ stains

- Clinical periodontal parameter:
  - Plaque index (PI)
- General oral hygiene assessment:
  - Number of missing teeth (indication of poor oral health)
  - Caries status (DMFT index)
    - Number of decayed teeth
    - Number of missing teeth
    - Number of filled teeth

<u>Soft tissue:</u>

- Clinical periodontal parameters:
  - Probing depth (PD)

  - Bleeding on probing (BOP)
  - Periodontal inflammation: if ≥ 6 sites with a (PD) of 4 to 8 mm in the upper and lower jaws.
  - Gingival Index (GI)
  - Clinical attachment loss (CAL)

- Self reported gingival issue:
  - Gingival inflammation
  - Gingival gum disease
  - Gingival bleeding/ bleeding gums
  - Pain in gums
  - Swelling gums

Combined measures:
Self reported dental health complains (including cavities, gum disease or dental stains)

- Periodontal index: community periodontal index

Para-dental health

OH-O2: Peri-implant oral health: (n=6)

- Clinical peri-implant parameters (n=6)
  - Plaque index (PI)
  - BOP
  - PD

- Radiographic peri-implant parameters (n=3)
  - Peri-implant bone loss (PIBL)
  - CAL

Respiratory Health (n= 32)

ResHO1: Lung Function (n=16)

- PEF
- FEV1/FVC
- FVC
- expiratory flow—25%, 50%, 75%
- FEV1
- FET

ResHO2: Respiratory Symptoms (n=26)

- Wheezing
- Difficulty breathing/ Shortness of breath
- Chest tightness
- Chronic cough
- Phlegm
- Respiratory symptoms grouped (CAT Score, bronchitic symptoms, asthma symptoms, asthma attack)
